# Modeling nonbreeding distributions of shorebirds and waterfowl in response to climate change

**DOI:** 10.1002/ece3.2755

**Published:** 2017-02-07

**Authors:** Gordon C. Reese, Susan K. Skagen

**Affiliations:** ^1^ U.S. Geological SurveyFort Collins Science CenterFort CollinsCOUSA

**Keywords:** climate change, conservation design, migratory shorebirds, species distribution models, wintering waterfowl

## Abstract

To identify areas on the landscape that may contribute to a robust network of conservation areas, we modeled the probabilities of occurrence of several *en route* migratory shorebirds and wintering waterfowl in the southern Great Plains of North America, including responses to changing climate. We predominantly used data from the eBird citizen‐science project to model probabilities of occurrence relative to land‐use patterns, spatial distribution of wetlands, and climate. We projected models to potential future climate conditions using five representative general circulation models of the Coupled Model Intercomparison Project 5 (CMIP5). We used Random Forests to model probabilities of occurrence and compared the time periods 1981–2010 (hindcast) and 2041–2070 (forecast) in “model space.” Projected changes in shorebird probabilities of occurrence varied with species‐specific general distribution pattern, migration distance, and spatial extent. Species using the western and northern portion of the study area exhibited the greatest likelihoods of decline, whereas species with more easterly occurrences, mostly long‐distance migrants, had the greatest projected increases in probability of occurrence. At an ecoregional extent, differences in probabilities of shorebird occurrence ranged from −0.015 to 0.045 when averaged across climate models, with the largest increases occurring early in migration. Spatial shifts are predicted for several shorebird species. Probabilities of occurrence of wintering Mallards and Northern Pintail are predicted to increase by 0.046 and 0.061, respectively, with northward shifts projected for both species. When incorporated into partner land management decision tools, results at ecoregional extents can be used to identify wetland complexes with the greatest potential to support birds in the nonbreeding season under a wide range of future climate scenarios.

## 1 Introduction

Protecting species that require resources across expansive, spatially heterogeneous, and temporally dynamic regions necessitates an advanced understanding of full life cycle ecology (Small‐Lorenz, Culp, Ryder, Will, & Marra, 2013). Many shorebirds and waterfowl depend on finding ample wintering, migratory, and breeding habitats, and knowing the circumstances under which particular habitat locations are selected could inform land protection, mitigation, and conservation efforts. Migration is inherently risky, in part because of challenges posed by the natural spatial and temporal variations in the condition of wetland habitats. Impending climate change may alter inundation patterns and the function of critical wetland habitats via, for example, increased evapotranspiration associated with higher temperatures (Johnson, Werner, & Guntenspergen, 2015; Johnson et al., 2010; Niemuth, Fleming, & Reynolds, 2014; Sofaer et al., 2016). In addition, humans continue to alter the landscape, including the conversion of land to uses that produce food and fuel (Hurlbert & Liang, 2012; Thomas, Lanctot, & Székely, 2006). The combined effects of changing climatic conditions and additional habitat loss could pose a substantial future threat to the persistence of waterfowl and migratory shorebirds.

Wetland availability across the North American Great Plains (hereafter Great Plains) is affected by both factors, as inundation state depends on rainfall and runoff in combination with evapotranspiration rates and land cover (Bartuszevige, Pavlacky, Burris, & Herbener, 2012; Cariveau, Pavlacky, Bishop, & LaGrange, 2011). Weather can vary considerably across the large regions crossed by long‐distance migrants (Millett, Johnson, & Guntenspergen, 2009; Tøttrup et al., 2008). The latest global climate models forecast more frequent extreme precipitation events, yet the southern Great Plains are expected to become drier as a result of increasing temperatures and evapotranspiration rates, leading to longer droughts (Swain & Hayhoe, 2014). Such changes can have large negative effects on freshwater wetlands (Kundzewicz et al., 2008), increasing erosion and wetland sedimentation during extreme events, reducing hydroperiods and the diversity of water regimes (Johnson et al., 2010), and changing landscape connectivity (McIntyre et al., 2014).

Playas and other wetlands within the Great Plains provide essential habitat for many wetland‐dependent vertebrate species and are especially important as migration and wintering areas for shorebirds and waterfowl (Cariveau & Pavlacky, 2009; Haukos & Smith, 1994; Skagen, Sharpe, Waltermire, & Dillon, 1999). The density of wetlands holding water positively affects the abundance and richness of waterfowl and shorebirds (Albanese & Davis, 2013; Webb, Smith, Vrtiska, & LaGrange, 2010), potentially representing the most important habitat features on the landscape. A large proportion of the wetlands in the Great Plains are ephemeral, resulting in a temporally dynamic hydrology that can have large implications to bird migration and wintering success (Albanese, Davis, & Compton, 2012; McIntyre et al., 2014; Naugle, Johnson, Estey, & Higgins, 2001).

The Great Plains are relatively flat, interspersed with wetlands, and fragmented with cropland and ranchland. A large proportion of the remaining wetland habitat is privately owned and therefore vulnerable to the societal dynamics that drive land‐use and land‐cover decisions, including agricultural activities (Detenbeck et al., 2002). Wetlands within the Great Plains are threatened by sediment accumulation (Burris & Skagen, 2013), increased summer temperatures, changing precipitation patterns, and declining hydroperiods due in part to agricultural intensification.

Among the many applications, species distribution models have been used to determine environmental relationships and potential impacts of climate change (Elith, Kearney, & Phillips, 2010; Heinanen & von Numbers, 2009). Our objective was to model current and future distributions of several *en route* migratory shorebirds and wintering waterfowl relative to land‐use patterns, the spatial distribution and composition of wetlands, and data from a wide range of global climate models. In general, our models estimated contemporary probabilities of occurrence and responses to climate change. We assumed that bird encounters can indicate the presence of suitable habitat and, therefore, predicted probabilities of occurrence could inform future conservation efforts.

## 2 Methods

### 2.1 Study location

**Figure 1:**
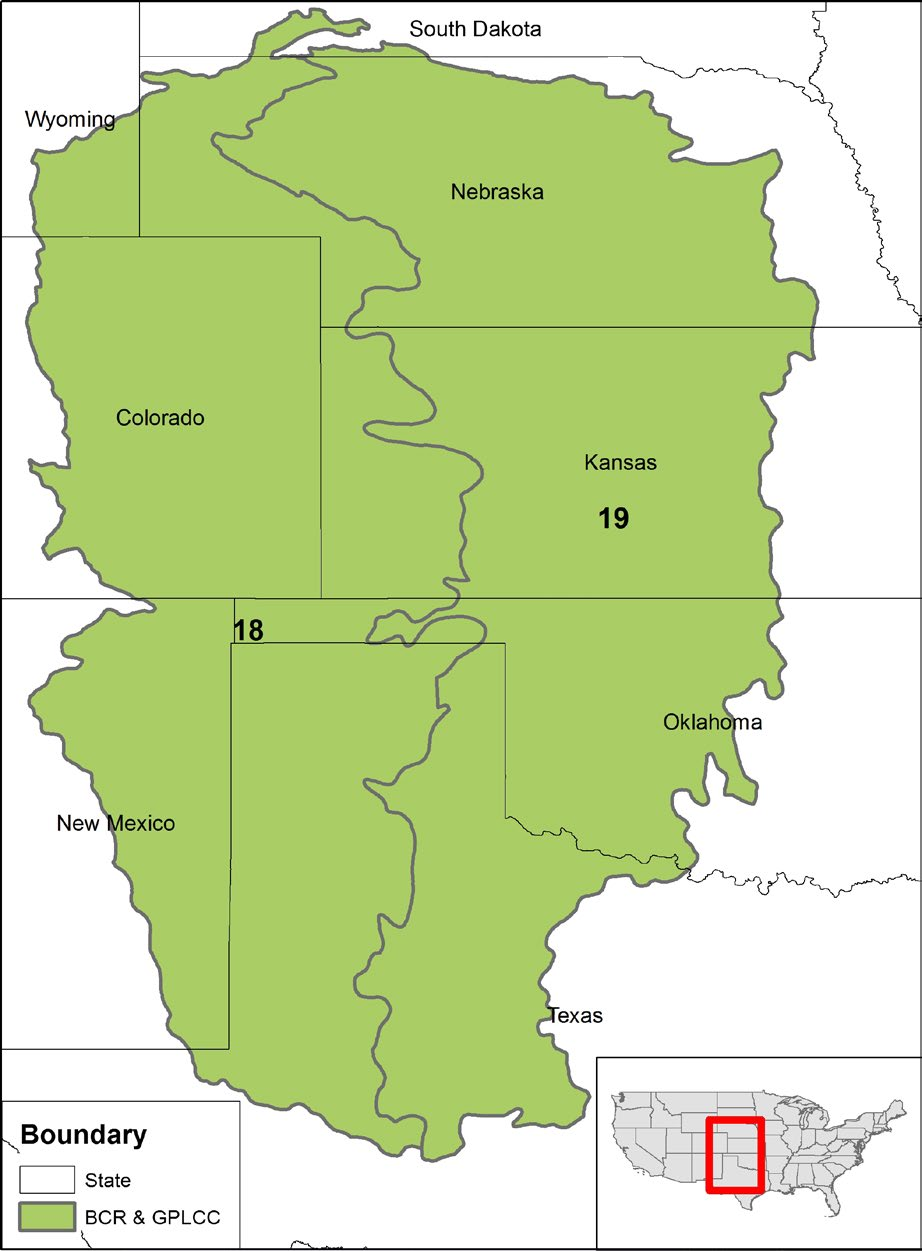
The Great Plains Landscape Conservation Cooperative (GPLCC) corresponds with the area delineated by Bird Conservation Regions (BCR) 18 and 19 in the south‐central Great Plains of North America

We modeled the distributions of several migratory shorebirds throughout spring migration, that is, stopover locations, as well as the winter, that is, December and January, distributions of two ducks (Table 1), within the boundary of the Great Plains Landscape Conservation Cooperative (GPLCC) (Figure 1), a public–private partnership that provides science assistance to natural resource managers within Bird Conservation Regions 18 and 19 (http://www.nabci-us.org/bcrs.htm). Relatively flat and once predominantly mixed‐grass and shortgrass prairie, the GPLCC encompasses a large portion (>782,000 km^2^) of the south‐central Great Plains (Figure 1). Approximately one‐half of the study area has been converted to agriculture, largely in the eastern regions.

**Table 1:** Focal waterfowl and shorebird species‐modeled, migration distance index, range of water depths used for foraging, and numbers of presences and absences in observation data

Common name	Scientific name	Alpha code	Migration distance (index)^a^	Range of water depths	Presences	Absences
Family Anatidae						
Mallard	Anas platyrhynchos	MALL	S	Wet–deep	2,804	3,319
Northern Pintail	Anas acuta	NOPI	S	Wet–deep	487	4,308
Family Charadriidae						
Mountain Plover	Charadrius montanus	MOPL	S (2.4)	Dry–2 cm	179	8,038
Family Recurvirostridae						
American Avocet	Recurvirostra americana	AMAV	S (2.1)	Dry–12 cm	2,385	7,226
Family Scolopacidae						
Willet	Tringa semipalmata	WILL	S (3.6)	Dry–10 cm	668	5,490
Lesser Yellowlegs	Tringa flavipes	LEYE	I (9.7)	Dry–10 cm	953	6,481
Whimbrel	Numenius phaeopus	WHIM	I (10.0)	Dry–12 cm	107	3,584
Long‐billed Curlew	Numenius americanus	LBCU	S (1.7)	Dry–9 cm	561	8,057
Marbled Godwit	Limosa fedoa	MAGO	S (3.5)	Dry–10 cm	329	4,280
Stilt Sandpiper	Calidris himantopus	STSA	L (15.0)	Wet–8 cm	414	5,322
Baird's Sandpiper	Calidris bairdii	BASA	L (16.7)	Wet–5 cm	655	7,063
Least Sandpiper	Calidris minutilla	LESA	I (9.1)	Wet–4 cm	737	7,153
White‐rumped Sandpiper	Calidris fuscicollis	WRSA	L (17.2)	Wet–5 cm	327	4,262
Semipalmated Sandpiper	Calidris pusilla	SESA	I (9.5)	Wet–4 cm	513	6,053
Long‐billed Dowitcher	Limnodromus scolopaceus	LBDO	I (8.9)	Wet–10 cm	698	6,821
Wilson's Phalarope	Phalaropus tricolor	WIPH	I (10.1)	Wet–deep	1,307	8,757

a Migration distance indices (× 1,000 km) for shorebirds, based on distances between breeding and wintering areas, are average of shortest distance, distance between midpoints, and distances between extreme edges of ranges (from Skagen & Knopf, 1993).

### 2.2 Observation data

We downloaded observation data from the eBird citizen‐science database (Sullivan et al., 2009) for the states intersected by the GPLCC boundary for 2002–2010, years that coincide with the beginning of the eBird program and the most recent year in the empirical climate data that we used, respectively. We clipped data to the GPLCC boundary and applied several filters including one based on the scale at which shorebirds optimally respond to habitat patterns, that is, 1.25–2 km (Albanese et al., 2012; Cunningham & Johnson, 2006. We maximized the number of observations available by applying the 2‐km threshold at several steps (see Fink et al., 2010; Sohl, 2014). We used all incidental observations, that is, from surveys with neither spatial nor temporal measures, and stationary counts, that is, from surveys with a known duration and across an area <30 m in diameter. For traveling and area counts, from surveys across known distances and areas, respectively, which also report to single point locations, we assumed a positive relationship between survey size and habitat heterogeneity and therefore only included traveling counts ≤4.023 km and area counts ≤1,257 ha (2‐km circular radius).

An important component of eBird data is a field for birders to specify whether or not all species were recorded, information useful to the assumption of absences. We eliminated absences from incidental surveys, considering them less reliable than those from the other survey types. As with the presences, we also eliminated those from traveling counts >4.023 km and those from area counts >1,257 ha. Additionally, we eliminated duplicate absences, that is, surveys on the same date and at the same location as another absence or presence.

We added data from two surveys conducted between mid‐March and mid‐May in 2008, accounting for approximately 5% of the observations used to build models. One survey included roadsides within a random selection of townships first stratified on wetland and cropland areas. Roadsides were surveyed twice, and all shorebirds within 400 m were recorded. Surveys of 25 sites with known shorebird usage were also included. On the ground, these data were collected similar to those classified as stationary counts in eBird; data from all sources, eBird and supplemental, were converted to presence and absence categories.

We mapped observation locations by date, compared them to frequency histograms (Skagen et al., 1999), and selected March 1 to June 15 as the range for spring migration. The largest number of migrants occurs toward the middle of a season (Dunn, Hussell, & Adams, 1997). To reduce sampling effects, we sorted data for each species by the survey date and clipped the smallest number of records that removed ≥5% from each tail, that is, the earliest and latest encounters within the spring migration window (see Farmer, Hussell, & Mizrahi, 2007).

### 2.3 Predictor variables

Migration is an interaction between space and time; therefore, we modeled spring stopover locations in the GPLCC with variables of each type, including latitude and day‐of‐year, that is, 1–365; 60–166 thus represents spring migration, March 1 to June 15 (see Gowan & Ortega‐Ortiz, 2014). Higher‐order date variables have been important predictors elsewhere (see Dunn et al., 1997; Farmer et al., 2007), so we also included the square of the day‐of‐year term. Geographic coordinate variables can account for small‐scale processes and address issues with spatial autocorrelation (Bailey & Gatrell, 1996; Sohl & Sayler, 2008).

Individual birds likely make decisions during migration reflecting both intrinsic and extrinsic factors such as body condition and proximal weather (Marra, Francis, Mulvihill, & Moore, 2005; Richardson, 1978). We elected to use weather data from Maurer, Wood, Adam, Lettenmaier, and Nijssen (2002) because they overlapped a large portion of the eBird data, 2002–2010, and were used to bias‐correct the global climate model data. Avian migration studies have found important predictors in temperature (Huin & Sparks, 2000) and wind (Green & Piersma, 2003; Richardson, 1978). Additionally, precipitation is often key to wetland condition. For example, Bartuszevige et al. (2012) found that playa inundation is largely a result of the precipitation events in the preceding 2 weeks.

We time‐matched observations to five month‐long weather variables including total precipitation and averages of the daily wind speed and minimum, maximum, and average temperature (Table 2). We matched observations that occurred in the first 15 days of a month to weather data from the previous month and remaining observations to the matching month. We also matched each record to the precipitation total from the previous calendar year, for example, all 2010 records, regardless of month, were matched to the total precipitation in 2009 for that location (see Johnson, Rice, Haukos, & Thorpe, 2011). In addition to phenological effects, weather variables can predict available wetland habitat because availability can vary as a function of precipitation, temperature, solar radiation, and wind speed (Albanese et al., 2012; Farmer & Wiens, 1998).

**Table 2:** Resolutions and sources of the predictor variables and global climate models used to model shorebird and waterfowl probabilities of occurrence during spring and winter, respectively, across the region delineated by the Great Plains Landscape Conservation Cooperative

Independent variable	Resolution	Source
Day‐of‐year, Day‐of‐year^2^	Point	Sullivan et al. (2009)
Latitude	Point	Sullivan et al. (2009)
Precipitation^a^, Temperature^b^, Wind	1/8°	Maurer et al. (2002)
Playas	Vector	U.S. Fish and Wildlife Service (2009); Playa Lakes Joint Venture (2014)
Lakes	Vector	U.S. Fish and Wildlife Service (2009)
Palustrine	Vector	U.S. Fish and Wildlife Service (2009); Playa Lakes Joint Venture (2014)
Rivers and riparian	Vector	U.S. Fish and Wildlife Service (2009); Playa Lakes Joint Venture (2014)
Cropland	30 m	Fry et al. (2011)
Grassland	30 m	Fry et al. (2011)
Terrain ruggedness index	30 m	U.S. Geological Survey (2009); Riley, DeGloria, & Elliot (1999)

Global climate model	Resolution	Modeling center
ACCESS1‐0	1/8°	Commonwealth Scientific and Industrial Research Organization and Bureau of Meteorology
CMCC‐CM	1/8°	Centro Euro‐Mediterraneo per I Cambiamenti Climatici
GFDL‐CM3	1/8°	NOAA Geophysical Fluid Dynamics Laboratory
INM‐CM4	1/8°	Institute for Numerical Mathematics, Russia
IPSL‐CM5B‐LR	1/8°	Institut Pierre‐Simon Laplace

a Precipitation variables included monthly and yearly totals.

b Temperature variables included the monthly average minimum, maximum, and mean.

We created wetland variables from unique features, that is, nonoverlapping, with data from the Playa Lakes Joint Venture (PLJV) and the National Wetlands Inventory (NWI) (Table 2). Shallow depressions that formed as a result of dissolution or wind deflation, playas occur throughout the Great Plains, fill intermittently, and occasionally provide crucial stopover and wintering sites for migratory birds (Haukos et al., 2006). The PLJV probable playas layer (version 4; PP4) includes some abrupt, artificial boundaries; therefore, we supplemented PP4 with unique playas separately provided by PLJV for Reagan and Upton counties, Texas (323 features), and with possible playas in Wyoming that we derived from NWI data (861 features). We removed seven playas intersecting perennial streams in the medium‐resolution National Hydrologic Dataset, for 90,305 total playas. We also created a Lake variable from NWI, a River variable by combining NWI riparian areas with PLJV riverine features, and a Palustrine variable by combining NWI ponds with PLJV uncategorized features. Duplicate features were removed from one of the variables, first from the PLJV supplemental layer and next from NWI, that is, PLJV playas were fully retained. In species distribution modeling, variables representing composition are more predictive than those representing distance (Johnson & Higgins, 1997). Separating feature types into different variables allowed wetland dynamics to vary, for example, across weather patterns. In order to minimize conversion error and utilize functions in ArcGIS (10.2), we converted wetland vectors to 10‐m‐resolution rasters, which resulted in <1% change in area, and computed the proportions of each variable within circular moving windows (2‐km radius).

Landscape context is also important to the state and condition of wetlands (Cariveau et al., 2011). For example, wetlands surrounded by native rangeland generally experience less sedimentation (Bartuszevige et al., 2012) and longer hydroperiods than those in cropland (Tsai, Venne, McMurry, & Smith, 2007). The inclusion of stationary land‐cover variables improves the accuracy of predictions even when models are projected using future climate scenarios (Sohl, 2014); therefore, we used the cropland and grassland classes from the National Land Cover Database (Fry et al., 2011) (Table 2) and similarly computed proportions within 2‐km circular moving windows. We computed the root‐mean‐square of elevation change across each 90 × 90 m area (RMSEC):

$$\text{RMSEC}=\sqrt{\left(\sum_{x=1}^{9}E_{x}^{2}\right)+\left(9E^{2}\right)-\left(2E\sum_{x=1}^{9}E_{x}\right)},$$

where *x* is the grid cell in each 3 × 3 matrix and *E* is elevation from the 30‐m National Elevation Dataset (U.S. Geological Survey, 2009), and created a terrain ruggedness index (TRI) as the averages of a 2‐km circular moving window.

Among many possible effects, climate change could alter the connectivity of wetlands across the Great Plains (McIntyre et al., 2014). General circulation models (GCMs), which forecast future climatic conditions, can be the largest source of uncertainty when predicting future species distributions; thus, a complementary subset of GCMs should be used (Stralberg et al., 2015). We selected five GCMs from the fifth phase of the Coupled Model Intercomparison Project (CMIP5) based on an increase of 8.5 W m^−2^ (RCP 8.5). We selected GCMs that represent a large range of the variation between the models using a scatter plot of the means of precipitation change and temperature change across the GPLCC (Talbert, personal communication; North Central Climate Science Center), including one near the average of all the GCMs and one from each of the quadrants relative to that point, for example, one hotter and drier GCM. The GCMs, and average forecasts relative to the central CMCC‐CM, were ACCESS1‐0 (hotter/drier), GFDL‐CM3 (hotter/wetter), INM‐CM4 (warmer/drier), and IPSL‐CM5B‐LR (warmer/wetter) (Table 2).

When comparing contemporary to future predictions, one should first project the model to contemporary GCM data (hindcast) because differences between the empirical and GCM data would otherwise contribute to the predicted changes in the probabilities of occurrence (Sofaer et al., 2016). We therefore projected models using GCM data averaged over 1981–2010 and 2041–2070, temporal ranges large enough to minimize the effects of natural variability. Specifically, we used the average annual total precipitation of each temporal range and the 30‐year averages for the appropriate month.

### 2.4 Modeling and evaluation

In response to the science needs of the GPLCC and the PLJV, we modeled nonbreeding distributions of two common waterfowl species and 14 shorebird species representing three families (Table 1). Collectively, these species range from short‐ to long‐distance migrants that use a broad range of habitat types, including arid grasslands, unvegetated mudflats, and shallow‐to‐deep water wetlands. At each observation location, we extracted a complete set of the empirical predictive variables from which we built models (Table 2). We also extracted variables across a 30 × 30 m lattice of points, including instead the GCM data, to which we projected the models.

We modeled probabilities of occurrence using Random Forests, a nonparametric method that combines the predictions from numerous trees. A tree is constructed from a bootstrap subsample of the observations and, for each branch on the tree, the data are split by selecting from a randomized subset of the predictor variables; this step minimizes modeling issues resulting from correlated variables (Breiman, 2001; Cutler et al., 2007). We were more interested in spatial predictions than inferences about the predictor variables, so reduction of correlation issues was a desirable property (see Dunn et al., 1997). Selected variables are used to hierarchically partition the data into increasing homogenous groups; thus, Random Forests inherently model variable interactions. We equalized the number of presences and absences at 80% of the number of presences for each species, specified that four randomly selected variables would be available for each branch, and built 4,500 trees for each forest.

We evaluated model performance with the out‐of‐bag observations, that is, observations not selected for a bootstrap sample, a standard Random Forests evaluation procedure. The class (presence or absence) of an out‐of‐bag observation is predicted using a majority vote across all of the trees where it was an out‐of‐bag observation. The reported out‐of‐bag error rate is the average across all observations. Given our binary response variable, probabilities of occurrence are the proportion of trees that classified the site as a presence. For the ecoregional‐level evaluations of probability of occurrence gain or loss, we used averages across the GPLCC.

We used the randomForest library (Breiman, Cutler, Liaw, & Wiener, 2014) with R version 3.1.1 (R Development Core Team, 2012) to build and test all models.

## 3 Results

### 3.1 Climate patterns

**Figure 2:**
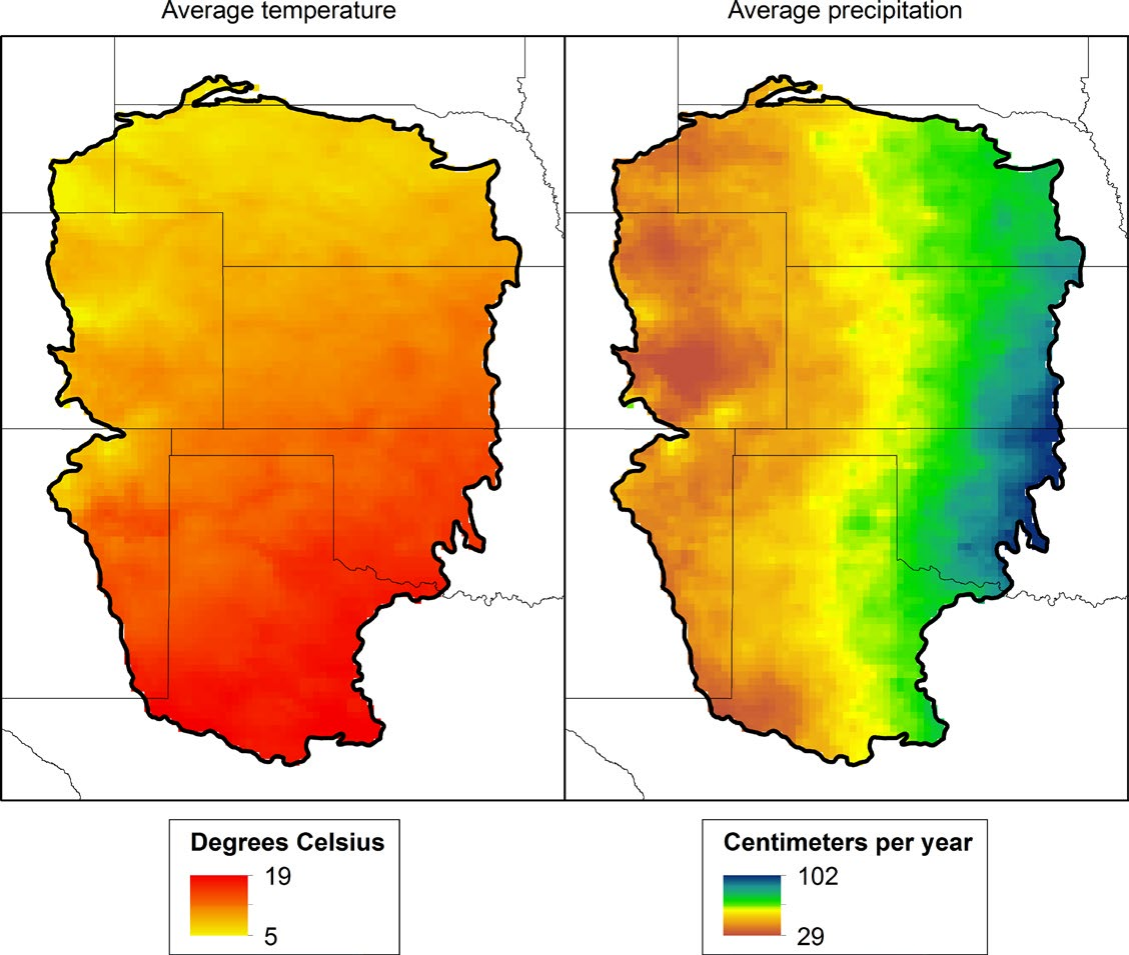
Thirty‐year contemporary averages of precipitation and temperature across the region delineated by the Great Plains Landscape Conservation Cooperative in the south‐central Great Plains of North America

**Figure 3:**
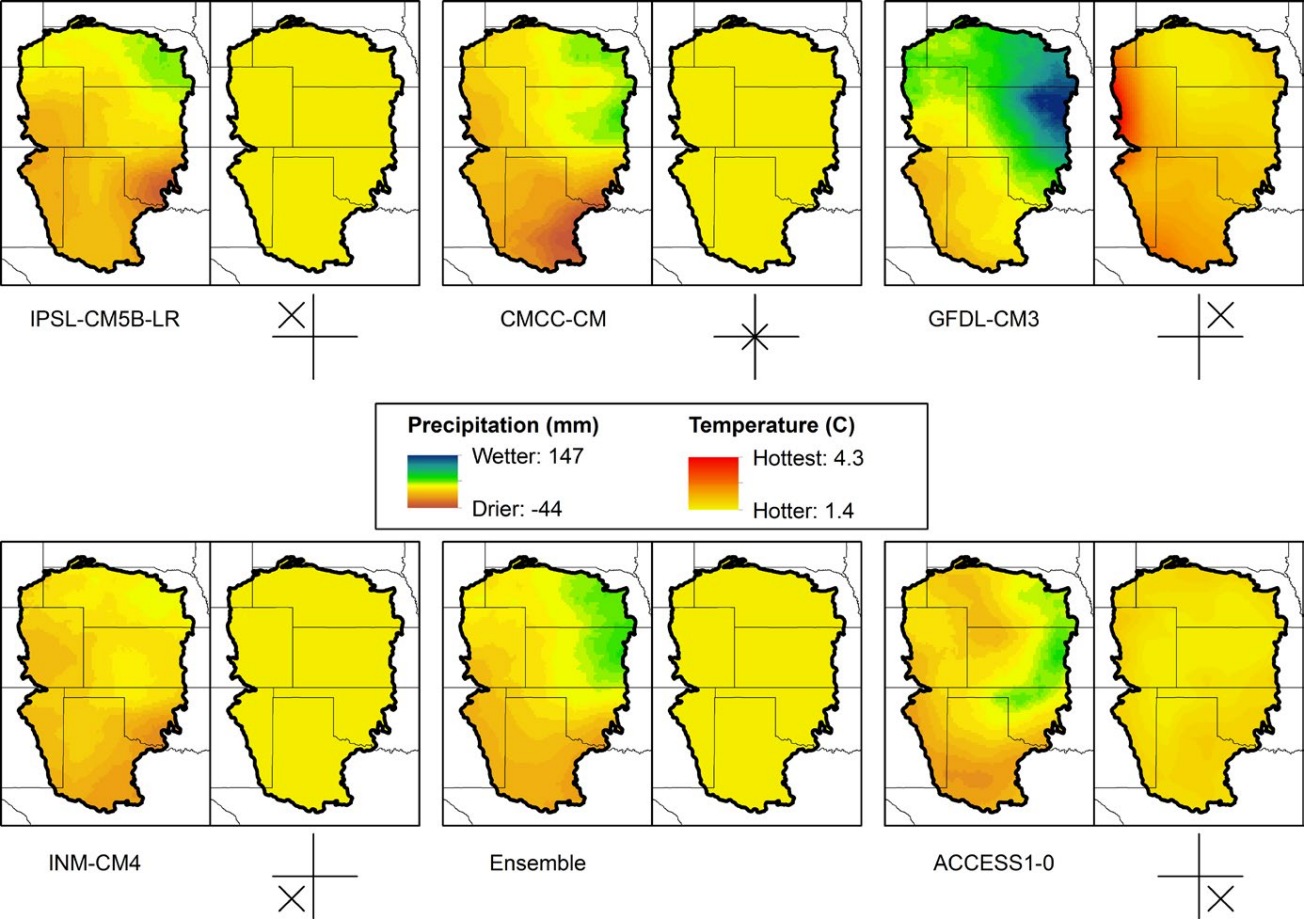
Predicted change in precipitation (mm; left panel each pair) and temperature (°C; right panel in each pair) based on future (2041–2070) minus contemporary (hindcast; 1981–2010) projections for five global circulation models (GCM) from CMIP5, Representative Concentration Pathway 8.5, and their ensemble mean. See Methods for definitions of GCMs. Crosshatches illustrate projected changes relative to the mean; wetter models are shown with an X above the horizontal line, warmer models with an X to the right of the vertical line, and the average model as an X in the center

Across the GPLCC region, there is a north–south gradient in average annual temperature ranging from 5°C to 19°C and a west–east gradient in annual precipitation ranging from 29 to 102 cm year^−1^ (Figure 2). Throughout the past several decades, average annual minimum and maximum temperatures have been rising across the region, and rainfall has varied substantially year‐to‐year. Relative to a 1951–1980 baseline period, the five GCMs estimated future climates (2041–2070) ranging from 16% drier to 20% wetter; averaged across the GCMs, rainfall increased by 2% (Figure 3). The five GCMs predicted varying future precipitation patterns in spring with the wetter GFDL‐CM3 model predicting the greatest increases in the northeastern portion of the region and the other models predicting drier conditions throughout most of the southern portions (Figure 3). The changes predicted in spring temperature varied both spatially and across the GCMs (ranging from 2.5°C to 4.0°C) and with GFDL‐CM3 predicting the highest temperatures, particularly in the western and southern portions of the region (Figure 3). An ensemble of the five GCMs revealed changes in precipitation throughout the region varying from −11 to +65 mm and changes in temperature from 2.2°C to 2.7°C (Figure 3).

### 3.2 General shorebirds patterns

Models generally performed better without longitude; thus, we built models without it. Across all species, out‐of‐bag error rates averaged 5.70% and ranged from 1.19 to 10.51% (Table 3).

**Table 3:** Out‐of‐bag error rates (%) and the numbers of absences and presences correctly and incorrectly predicted. Latin names for shorebird species are provided in Table 1

	Error rate	Empirical absence		Empirical presence	
		Predicted absence	Predicted presence	Predicted absence	Predicted presence
Mountain Plover	3.01	7,814	224	23	156
American Avocet	2.65	7,155	71	184	2,201
Willet	2.84	5,397	93	82	586
Lesser Yellowlegs	10.51	5,899	582	199	754
Whimbrel	1.19	3,555	29	15	92
Long‐billed Curlew	3.19	7,843	214	61	500
Marbled Godwit	2.65	4,199	81	41	288
Stilt Sandpiper	3.24	5,181	141	45	369
Baird's Sandpiper	7.93	6,571	492	120	535
Least Sandpiper	8.25	6,629	524	127	610
White‐rumped Sandpiper	7.56	3,967	295	52	275
Semipalmated Sandpiper	7.63	5,650	403	98	415
Long‐billed Dowitcher	9.23	6,257	564	130	568
Wilson's Phalarope	9.85	7,895	862	129	1,178

The average probabilities of occurrence (ensembles of the five GCMs) in the contemporary (hindcast; 1981–2010) and future (forecast; 2041–2070) projections were largest for Wilson's Phalarope, Lesser Yellowlegs, and Long‐billed Dowitcher (≥0.46) and lowest for Whimbrel, Marbled Godwit, and American Avocet (≤0.22; Table 4) during peak migration. Northbound flights of several early migrants, including Baird's Sandpiper, Lesser Yellowlegs, Least Sandpiper and others, began before April, and migration flights continued through the end of May and beyond for later migrants such as White‐rumped Sandpiper and Wilson's Phalarope (Table 4). Long‐distance migrants such as Stilt Sandpipers and White‐rumped Sandpipers were most likely to occur in the region in mid‐May. Two species that breed in the region, the American Avocet and Long‐billed Curlew, spent the most time in the GPLCC, beginning migration before April and remaining in the area at least through the end of our migration window.

**Table 4:** Probabilities of occurrence of 14 shorebird species during spring migration through the region delineated by the Great Plains Landscape Conservation Cooperative. Probability values are the ensemble of five global climate models from CMIP5, averaged across the region, and provided for both hindcasts (contemporary) and forecasts (future)

	Contemporary (1981–2010)					Future (2041–2070)				
	March 30	April 16	April 30	May 16	May 30	March 30	April 16	April 30	May 16	May 30
Mountain Plover		0.29	0.26	0.19	0.20		0.27	0.23	0.18	0.20
American Avocet	0.19	0.20	0.21	0.22	0.21	0.19	0.21	0.21	0.25	0.25
Willet			0.30	0.18	0.14			0.29	0.20	0.17
Lesser Yellowlegs	0.30	0.46	0.48	0.38		0.33	0.47	0.48	0.39	
Whimbrel		0.18	0.16	0.20			0.19	0.17	0.22	
Long‐billed Curlew	0.41	0.36	0.32	0.23	0.24	0.40	0.35	0.31	0.23	0.24
Marbled Godwit		0.16	0.20	0.13			0.17	0.19	0.14	
Stilt Sandpiper		0.14	0.18	0.38			0.20	0.25	0.39	
Baird's Sandpiper	0.29	0.37	0.39	0.38		0.32	0.40	0.42	0.41	
Least Sandpiper	0.21	0.37	0.43	0.39		0.26	0.41	0.46	0.40	
White‐rumped Sandpiper			0.24	0.46	0.38			0.28	0.47	0.39
Semipalmated Sandpiper		0.29	0.35	0.35			0.32	0.37	0.36	
Long‐billed Dowitcher	0.23	0.37	0.47	0.38		0.27	0.40	0.47	0.38	
Wilson's Phalarope			0.62	0.55	0.37			0.60	0.55	0.39

Probability of occurrence patterns differed among the species, with short‐distance migrants Mountain Plovers, Willets, and Long‐billed Curlews occurring primarily in the western and northern portions of the study area, and several short‐to‐intermediate distance species occurring throughout (American Avocet, Whimbrel, Marbled Godwit, and Wilson's Phalarope; Table 5, Appendix S1). The Lesser Yellowlegs, Least Sandpiper, and Long‐billed Dowitcher occurred throughout, but with marginally larger probabilities of occurrence in the eastern portion of the GPLCC; the remaining four calidridines, including three long‐distance species, occurred primarily in eastern regions, that is, BCR 19 (Table 5, Appendix S1). Northward movement patterns across the spring migration window are apparent in the probabilities of occurrence of many of the species, notably Lesser Yellowlegs, Baird's Sandpiper, Least Sandpiper, and Long‐billed Dowitcher (Appendix S1).

**Table 5:** General distribution pattern of shorebirds across the Great Plain Landscape Conservation Cooperative region relative to migration distance and foraging habitat

Western/northern distribution			Distributed across study area			Distributed across study area but more in eastern side			Eastern distribution		
Species code	Migration distance (index)	Range of water depths (cm)	Species code	Migration distance (index)	Range of water depths (cm)	Species code	Migration distance (index)	Range of water depths (cm)	Species code	Migration distance (index)	Range of water depths (cm)
MOPL	S (2.4)	Dry–2	AMAV	S (2.1)	Dry–12	LEYE	I (9.7)	Dry–10	STSA	L (15.0)	Wet–8
WILL	S (3.6)	Dry–10	WHIM	I (10.0)	Dry–12	LESA	I (9.1)	Wet–4	BASA	L (16.7)	Wet–5
LBCU	S (1.7)	Dry–9	MAGO	S (3.5)	Dry–10	LBDO	I (8.9)	Wet–10	WRSA	L (17.2)	Wet–5
			WIPH	I (10.1)	Wet/deep				SESA	I (9.5)	Wet–4

Migration distance index and water depths from Skagen and Knopf (1993) and Skagen et al. (1999).

**Figure 4:**
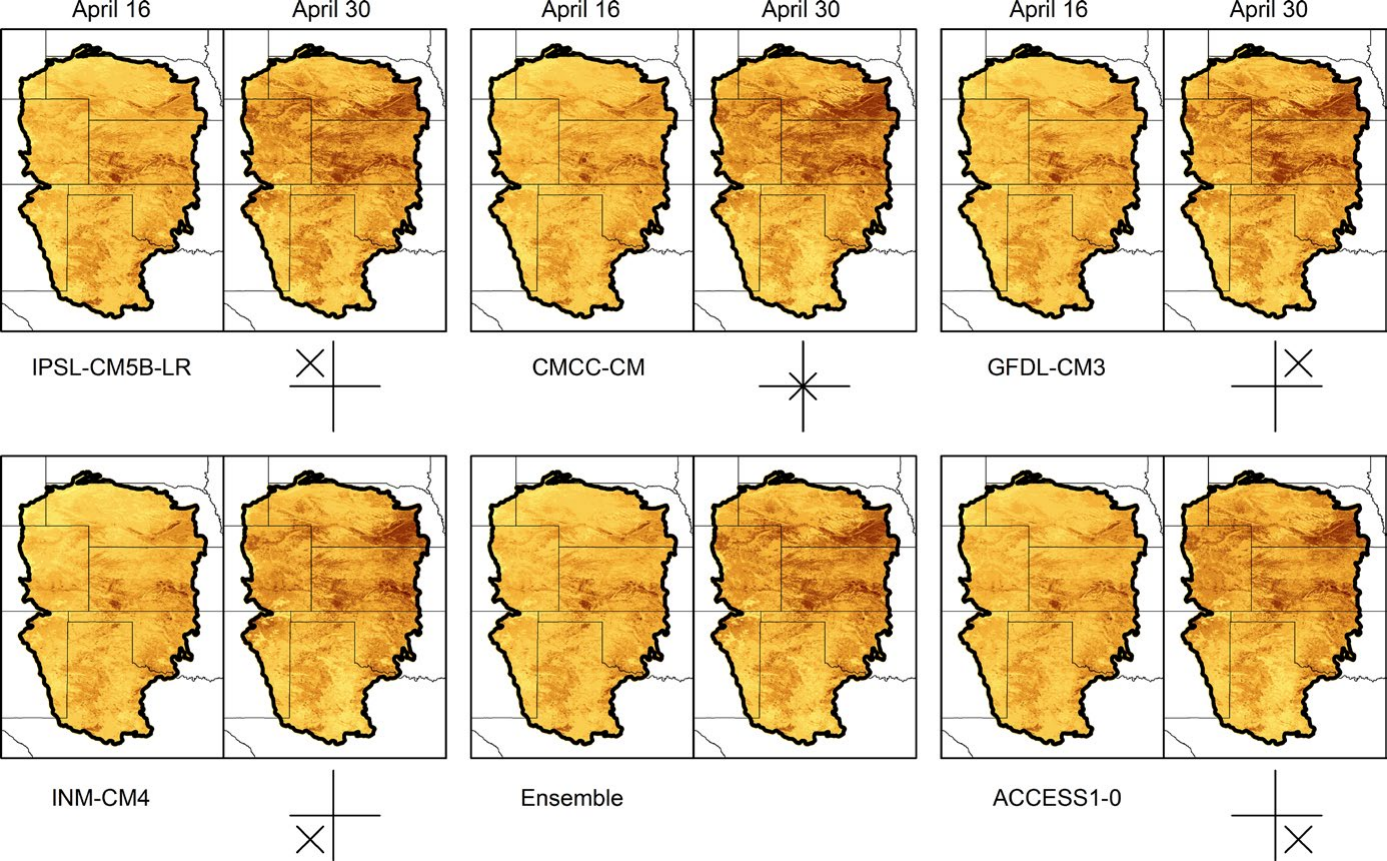
Probability of occurrence of Long‐billed Dowitcher during mid‐migration in 2041–2070 (forecast) based on five general circulation models (GCM) from CMIP5, Representative Concentration Pathway 8.5, and the ensemble of the GCMs. The yellow‐to‐brown color ramp corresponds to small‐to‐large probability values. The diagrams indicate the position of GCM relative to changes in precipitation and temperature with wetter models appearing as an X above the horizontal line, warmer models as an X to the right of the vertical line, and the average model as an X in the center

The five GCMs resulted in small differences in future probabilities of occurrence, as illustrated with predictions for the Long‐billed Dowitcher in the middle of migration (Figure 4). Probabilities of occurrence were slightly larger with the two hottest GCMs, GFDL‐CM3 and ACCESS1‐0, in mid‐April than the remaining models, and slightly smaller with the driest model, INM‐CM4, than all other models. Probabilities of occurrence for all GCMs and species are presented in Appendix S2.

### 3.3 Projected changes in probabilities of occurrence of shorebirds

Overall, changes in probabilities of occurrence differed among GCMs and among species relative to their spatial distribution across the region. Averaged across all locations, species, and models, probabilities of occurrence increased by 0.011–0.023, with the largest increase early in migration (Table 6). Included in (and concealed by) these averages were overall decreases for Mountain Plovers (−0.015) and Long‐billed Curlews (−0.005), and decreases for Marbled Godwits, Willets, and Wilson's Phalaropes during specific time periods (late April to mid‐May; Table 6). Models predicted additional declines for some species in early migration (Appendix S3); changes ranged from −0.042 to 0.094.

**Table 6:** Probability of occurrence changes (forecast minus hindcast) of 14 shorebird species during spring migration through the region delineated by the Great Plains Landscape Conservation Cooperative. Probability values are the ensemble of five global climate models from CMIP5, averaged across the region. Latin names are provided in Table 1

	March 30	April 16	April 30	May 16	May 30	Average
Mountain Plover		−0.025	−0.025	−0.005	−0.005	−0.015
American Avocet	0.001	0.011	0.005	0.033	0.037	0.018
Willet			−0.018	0.023	0.029	0.011
Lesser Yellowlegs	0.028	0.007	0.005	0.010		0.013
Whimbrel		0.013	0.019	0.014		0.016
Long‐billed Curlew	−0.008	−0.007	−0.009	0.000	−0.002	−0.005
Marbled Godwit		0.007	−0.005	0.003		0.002
Stilt Sandpiper		0.056	0.064	0.016		0.045
Baird's Sandpiper	0.023	0.034	0.031	0.028		0.029
Least Sandpiper	0.056	0.042	0.029	0.013		0.035
White‐rumped Sandpiper			0.049	0.010	0.016	0.025
Semipalmated Sandpiper		0.031	0.022	0.008		0.020
Long‐billed Dowitcher	0.039	0.031	0.000	0.001		0.018
Wilson's Phalarope			−0.011	−0.003	0.023	0.003
Across species average	0.023	0.018	0.011	0.011	0.016	0.016

Potential shifts in spatial distribution are more apparent in maps portraying the actual changes in probabilities in occurrence. Maps portraying predicted changes in the probabilities of occurrence, for both the average of the five GCMs (ensemble) and the hottest and driest model, ACCESS1‐0, are provided in Appendix S4 for all species. Interpretation of the predicted changes is best made in conjunction with the contemporary distributions, so that changes can be related to the probabilities of occurrence in particular areas. For example, early in migration, increases are projected for the Lesser Yellowlegs in areas of Kansas and Oklahoma where the contemporary probabilities of occurrence were relatively small (Appendix S4d). In contrast, during the middle of migration, decreases in occurrence were projected in prime habitat in northern Kansas (ensemble) and throughout Kansas (ACCESS1‐0, hot/dry).

**Figure 5:**
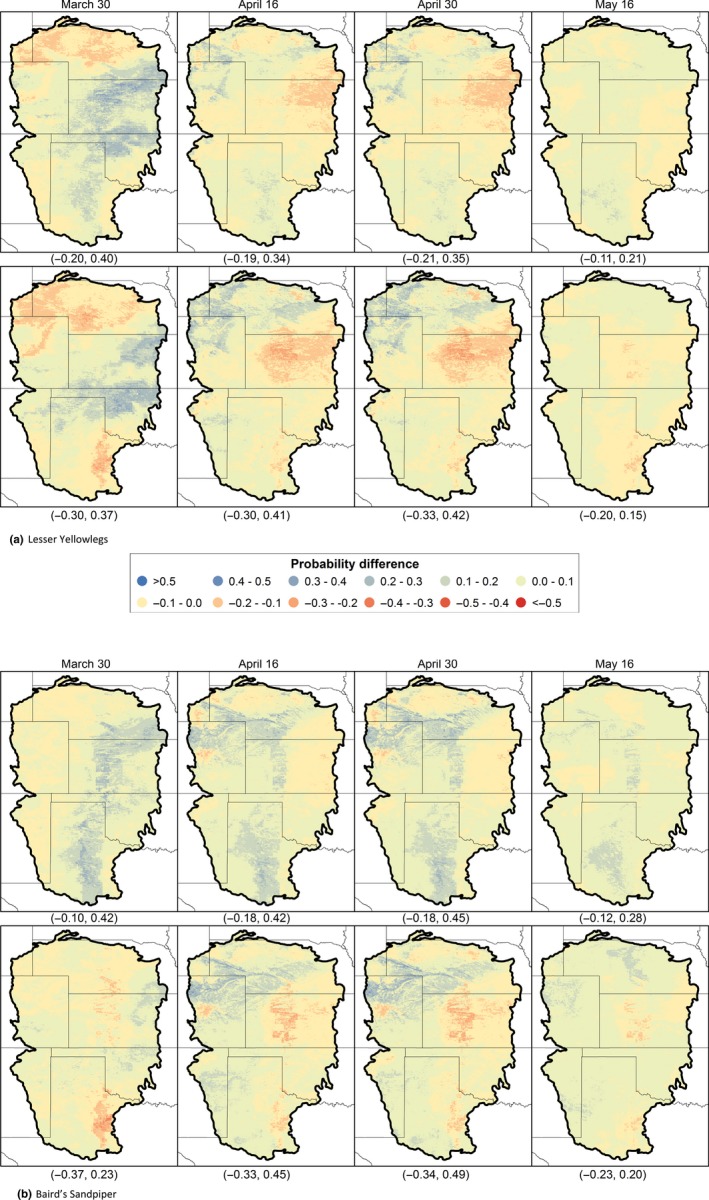
Projected changes in probabilities of occurrence of (a) Lesser Yellowlegs and (b) Baird's Sandpiper based on two general circulation models, the warmer wetter IPSL‐CM5B‐LR, Representative Concentration Pathway 8.5 (top panels), and the hotter drier ACCESS1‐0, Representative Concentration Pathway 8.5 (bottom panels). Minimum and maximum values across the study area are provided in parentheses

Predicted change varies among the GCMs, as portrayed for two species, the Lesser Yellowlegs and Baird's Sandpiper, and two GCMs, the relatively warm/wet IPSL‐CM5B‐LR and hot/dry ACCESS1‐0 (Figure 5). Overall, probabilities of occurrence for the Lesser Yellowlegs and Baird's Sandpiper increased by 0.013 and 0.029, respectively (Table 6), yet there were areas with predicted declines as large as −0.37 and increases of 0.49 (Figure 5b).

Shorebirds with more westerly or northerly distributions exhibited the largest predicted declines in probability of occurrence with an average change of −0.003 (Tables 5 and 6). Mountain Plover occurrences were predicted to decline in April and in mid‐late May in the southern and western portions of the GPLCC, respectively, particularly in their stronghold in eastern Colorado and northeastern New Mexico (Appendix S4), with an average decline in probability of occurrence of −0.015. The largest probabilities for Willets occurred early in migration, and declines were predicted in northern Nebraska. In contrast, the largest probabilities of occurrence for Long‐billed Curlews existed in the northern half of Nebraska although small pockets of declines were predicted there (Appendix S4); their overall predicted decline in probability of occurrence was −0.005.

Shorebirds with a more easterly distribution, mostly long‐distance migrants, had an average increase in probability of occurrence of 0.030 across the region during spring migration. The four species in this group, Stilt, Baird's, White‐rumped, and Semipalmated Sandpipers, were predicted to increase in occurrence to varying degrees during April and mid‐May in western Nebraska and eastern Colorado where they currently have low occurrence rates. Using the hotter/drier climate model, ACCESS1‐0, probabilities of occurrence for this group declined in central Kansas, an area with relatively large occurrence probabilities.

The two groups distributed more evenly across the study area were intermediate in response, with average predicted increases in occurrence of 0.009 and 0.022 for western and eastern species (Table 6), respectively. Of these, the more eastern species, Lesser Yellowlegs, Least Sandpipers, and Long‐billed Dowitchers, exhibited the same patterns as the more eastern group described above, with potential increases in occurrence in the northwestern part of the region and potential declines in central Kansas if conditions become hotter and drier.

### 3.4 General waterfowl patterns and projected changes

**Figure 6:**
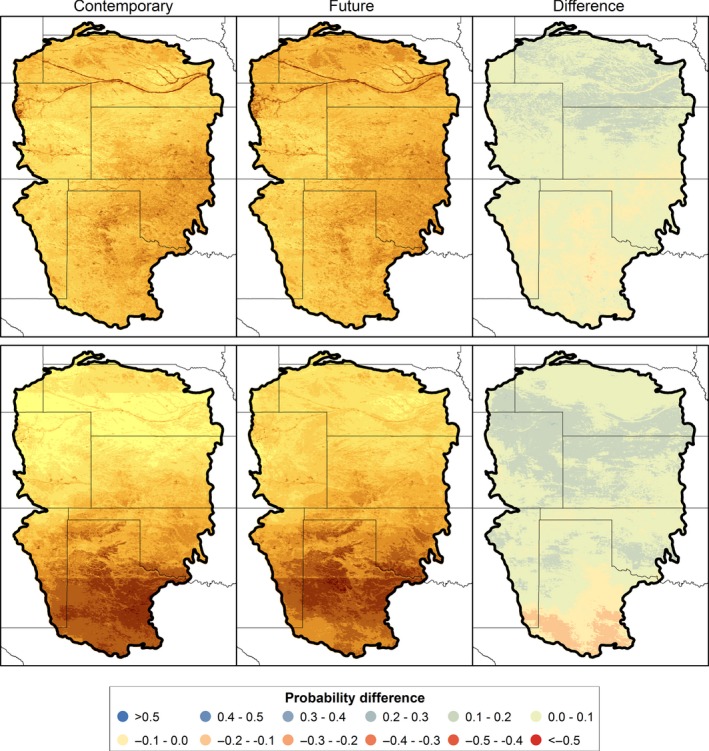
Probability of occurrence of Mallard (top panels) and Northern Pintail (bottom panels) based on the ensemble of five general circulation models from CMIP5, Representative Concentration Pathway 8.5. Contemporary map is based on 1981–2010 (hindcast) and future map on 2041–2070 (forecast) climate data. The yellow‐to‐brown color ramp corresponds to small‐to‐large probability values. Difference panels display the probability differences, future (forecast) minus contemporary (hindcast)

Wintering waterfowl are distributed throughout the GPLCC region during December and January with average probabilities of occurrence of 0.356 and 0.367 for Mallard and Northern Pintail, respectively. Northern Pintail were concentrated in the Texas panhandle (Figure 6). Averaged across the five GCMs, Mallards are predicted to shift northward, with increased probabilities of occurrence in Kansas, Nebraska, and Colorado and declines in Texas. Increases in occurrence probabilities of Northern Pintails were predicted throughout the region with the exception of an area of decline along the southern GPLCC boundary (Figure 6). Across the region, the probabilities of occurrence of Mallards and Northern Pintail are expected to increase by 0.046 and 0.061, respectively. All five of the climate models project region‐wide increases in probability of occurrence (Table 7).

**Table 7:** Probabilities of occurrence of waterfowl species during winter (December–January) in the region delineated by the Great Plains Landscape Conservation Cooperative. Probability values are the ensemble of five global climate models from CMIP5, averaged across the region, and provided for hindcasts (contemporary), forecasts (future), and the predicted change (forecast minus hindcast)

		Contemporary	Future	Differences
Mallard	ACCESS1‐0	0.362	0.405	0.043
	CMCC‐CM	0.362	0.398	0.037
	GFDL‐CM3	0.358	0.410	0.052
	INM‐CM4	0.349	0.377	0.028
	IPSL‐CM5B‐LR	0.350	0.423	0.072
	Ensemble	0.356	0.402	0.046
Northern pintail	ACCESS1‐0	0.375	0.448	0.073
	CMCC‐CM	0.373	0.414	0.041
	GFDL‐CM3	0.367	0.436	0.069
	INM‐CM4	0.365	0.400	0.035
	IPSL‐CM5B‐LR	0.357	0.443	0.086
	Ensemble	0.367	0.428	0.061

## 4 Discussion

Although we did not directly model hydrologic responses to climate in order to forecast future occurrence patterns of birds, we assumed that the presence of birds on the landscape reflected the underlying habitat suitability. Collectively, studies of wetland systems across the Great Plains have documented strong linkages between rainfall patterns and wetland density (Bartuszevige et al., 2012; Cariveau et al., 2011; Sofaer et al., 2016) and, in turn, between wetland density and wetland bird abundance (Austin, 2002; Niemuth & Solberg, 2003; Steen, Skagen, & Noon, 2014). Therefore, modeling bird occurrences in the absence of data on wetland condition can nonetheless provide valuable clues as to the projected spatial distribution of suitable habitat for birds.

Our findings suggest that when aggregated across our entire study area and across a 30‐year time period, the condition of migration stopover sites for shorebirds that can shift in space and time may be marginally improved by changing climate. Exceptions to this generalization are the two upland species, Mountain Plover and Long‐billed Curlew, which may experience slight declines in their probabilities of occurrence. Spatial shifts within the region are expected to some degree for many of the species, however, implicating the need for adequate habitats and resources to be maintained or made available in the future target areas. Our 30‐year projections do not address the inter‐annual variability that currently exists and will likely persist into the future. As a result, shorebird populations may need to shift spatially on a year‐to‐year basis. If populations are unable to shift in space, either because of intrinsic factors (e.g., lack of plasticity in choosing routes) or extrinsic factors (lack of suitable habitat in climatically favorable areas), populations within the study area during spring migration may be negatively impacted by changing climate.

Fortunately, *en route* migrant shorebirds appear to have low site fidelity, selecting landscape features opportunistically (Ambrosini et al., 2014; Skagen, Granfors, & Melcher, 2008; Skagen & Knopf, 1993; Warnock, Haig, & Oring, 1998). Such behavioral plasticity represents a natural adaptation to environmental change and contributes to the adaptive capacity of a species which allows it to cope with climate change “with minimal disruption” (Glick, Stein, & Edelson, 2011).

Recent evidence reveals that long‐distance migrating terrestrial birds have a greater capacity than short‐distance migrants to adjust *en route* migration timing and trajectories (location during migration) in response to environmental variation (La Sorte & Fink, 2017). This is in part because long‐distance migrants presumably experience the selective pressure imposed by greater degrees of environmental variation. Consistent with this viewpoint, predicted increases in probabilities of occurrence of shorebirds in this study were positively related to migration distance (*F*_1,12_ = 12.3, *p* = .04), suggesting greater flexibility of longer distance migrants.

The speed of migration could influence the degree to which bird species can respond to climate change (Hurlbert & Liang, 2012). Some of the predicted shifts in probability of occurrence in our study suggest that shorebirds may advance their migration calendars in response to weather. Most assessments of the effects of climate change on avian migration phenology have used data from arrival areas on the breeding grounds, and far fewer have used field data from passage areas (Gordo, 2007; but see La Sorte & Fink, 2017). Weather, and in particular temperature in the northern hemisphere, plays a large role in the speed and timing of migration (Chambers, Beaumont, & Hudson, 2014; Gordo, 2007). Although photoperiod is generally accepted as the primary trigger for onset of migration (Gwinner, 1996), environmental conditions in departure areas allow birds to fine‐tune their departure dates. Furthermore, weather patterns encountered *en route* may influence progression speed in response to wind favorability and stopover duration in response to body condition and food supplies (Gordo, 2007). Rainfall can slow passage rates, but can also greatly influence the availability of stopover habitats in the dynamic wetlands across the Great Plains (Bartuszevige et al., 2012; Cariveau et al., 2011; Gordo, 2007; Sofaer et al., 2016). Wind, including speed and direction aloft, is also an important factor for migration (Thorup & Rabol, 2001).

Here, we deliberately specify weather, rather than climate, as it has proven to be a better predictor of vagile species (i.e., *en route* migrants, nomads) and is needed for modeling yearly variations. Predictions based on climate can overestimate the “availability of suitable habitat and species climatic tolerances, masking species potential vulnerability to climate change” (Reside, VanDerWal, Kutt, & Perkins, 2010). Our empirical models based on historic weather and bird survey data captured the tight correspondence of bird occurrence and weather because of the small time frame (1 month) within which we associated birds with locations and weather variables.

There remains considerable uncertainty regarding the factors used as signals for speed of migration (Marra et al., 2005). Empirical models of habitat used during active migration appear to be a rare (but see Gowan & Ortega‐Ortiz, 2014) and potentially difficult endeavor (Joseph & Stockwell, 2000). Not surprisingly, there has been considerably more modeling work completed on the relatively static periods of the annual cycle, that is, breeding and wintering habitat, as well as on the dates of first encounter at some geographic location, often based on data availability (e.g., Chambers et al., 2014; Murphy‐Klassen, Underwood, Sealy, & Czyrnyj, 2005).

Distribution models are possibly the best available tool for predicting responses to changes in habitat and environmental conditions (but see Sinclair, White, & Newell, 2010). To our knowledge, modeling probabilities of occurrence across a season of active migration with empirical data is a relatively unique approach; others have modeled migration directly using mechanistic models (e.g., Lonsdorf et al., 2016). Projections using the most current climate model data indicate that the probabilities of occurrence of shorebirds and waterfowl in our region of the Great Plains would remain largely unchanged, but that migration could occur earlier (supporting numerous studies) and that suitable climate would shift predominantly westward, supporting Currie and Venne (2016). However, it should be noted that Random Forests are more likely than some other modeling methods to predict such spatial shifts (Beaumont et al., 2016). Additionally, that the modeled species seem mostly adaptable to current climate projections (at least until 2060), suggests that others factors might pose larger threats, for example, land‐use change (see Titeux, Henle, Mihoub, & Brotons, 2016).

Many shorebird species are experiencing long‐term population declines with habitat loss and anthropogenic threats as likely factors (Andres et al., 2012). Lesser Yellowlegs are categorized as in Significant Decline, and Mountain Plover and Whimbrel are classed as Apparent Decline by representatives of the Canadian Shorebird Conservation Plan and the U.S. Shorebird Conservation Plan (Andres et al., 2012). Furthermore, although considered stable across the past decade, Long‐billed Curlew, Marbled Godwits, and Stilt Sandpipers have been in Apparent Decline over the past 30 years. The population trend status of Baird's Sandpiper and Long‐billed Dowitcher are unknown.

Migrant shorebirds and waterfowl require wetland complexes for food and protection (Farmer & Parent, 1997; Hoekman, Mills, Howerter, Devries, & Ball, 2002; Skagen, 2006), but losses of potentially usable wetlands have already exceeded 50% in the conterminous USA (Dahl, 2000 ; Keddy et al., 2009) due to a combination of pressures from agriculture, development, and sedimentation (Luo, Smith, Allen, & Haukos, 1997). In addition, nearly all freshwater wetland loss since the mid‐1980s has occurred in the Great Plains (Dahl, 2000). Protections favor those wetlands that are relatively permanent, although the suitability of larger wetlands can be altered by the presence of smaller ones (Naugle et al., 2001). In addition, wetland complexes offer the variety of habitat preferred by some species.

Through the aggregation of newly emerging spatial tools, managers can address societal needs while basing ultimate decisions on sound science and landscape ecology foundations. The findings of our study have applicability to ongoing research and management efforts within the Great Plains Landscape Conservation Cooperative boundary. An advantage to conducting our analyses regionally, rather than from national or hemispheric perspectives, is that findings are sufficiently fine‐tuned to incorporate into land management and acquisition decisions. The findings allow our partners, the Rainwater Basin Joint Venture (RWBJV) and PLJV, to evaluate their current habitat priorities and delivery actions for nonbreeding shorebirds and waterfowl and to identify new landscapes to target for current or future conservation action, thereby enhancing the adaptive capacity of target species in the face of climate change (Stein et al., 2013). These findings, when used in combination with the Decision Support Tools of the RWBJV and the landscape‐design planning process undertaken by the PLJV (2014), can lead to site‐ and time‐specific water management options that may help to counteract the detrimental effects of climate change on migrating and wintering wetland birds (Uden et al., 2015). In addition, because there is substantial uncertainty regarding future climate projections, the application of risk diversification tools such as Modern Portfolio Theory to conservation planning may serve to optimize spatial targeting of conservation actions (Ando & Mallory, 2012).

## Conflict of Interest

None declared.
