# Supplementary material for: Modeling nonbreeding distributions of shorebirds and waterfowl in response to climate change
Source: Ecol Evol. 2017 Feb 7;7(5):1497–513. doi: 10.1002/ece3.2755 (PMC5330909; doi:10.1002/ece3.2755)
Supplement: Supplementary file 1 [file ECE3-7-1497-s001.docx]

**
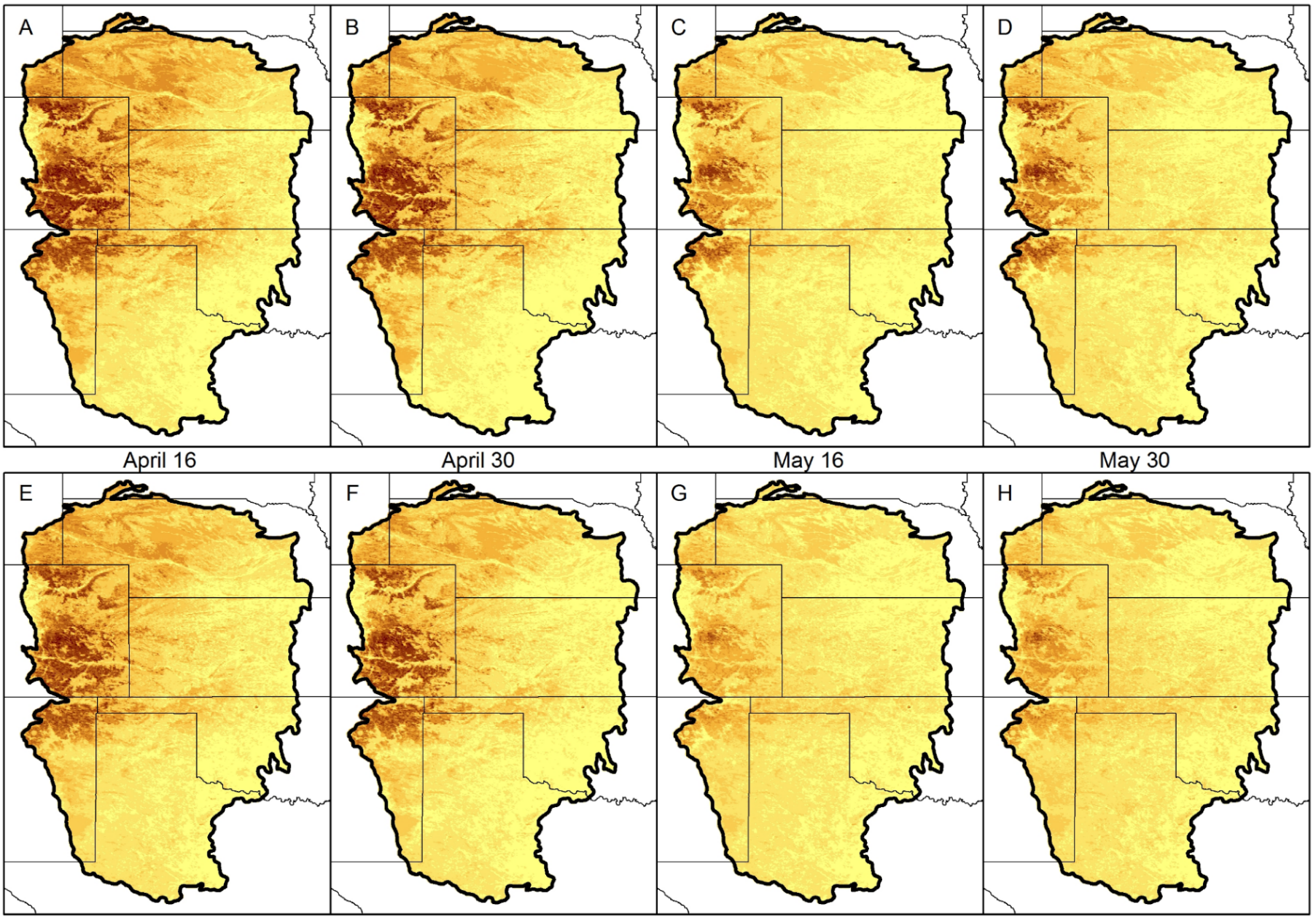
**

**Appendix 1a.** Probability of occurrence of Mountain Plover based on the ensemble of five general circulation models from CMIP5, Representative Concentration Pathway 8.5. The top row is based on 1981-2010 (hindcast) and the bottom row on 2041-2070 (forecast) climate data. The yellow-to-brown color ramp corresponds to small-to-large probability values.


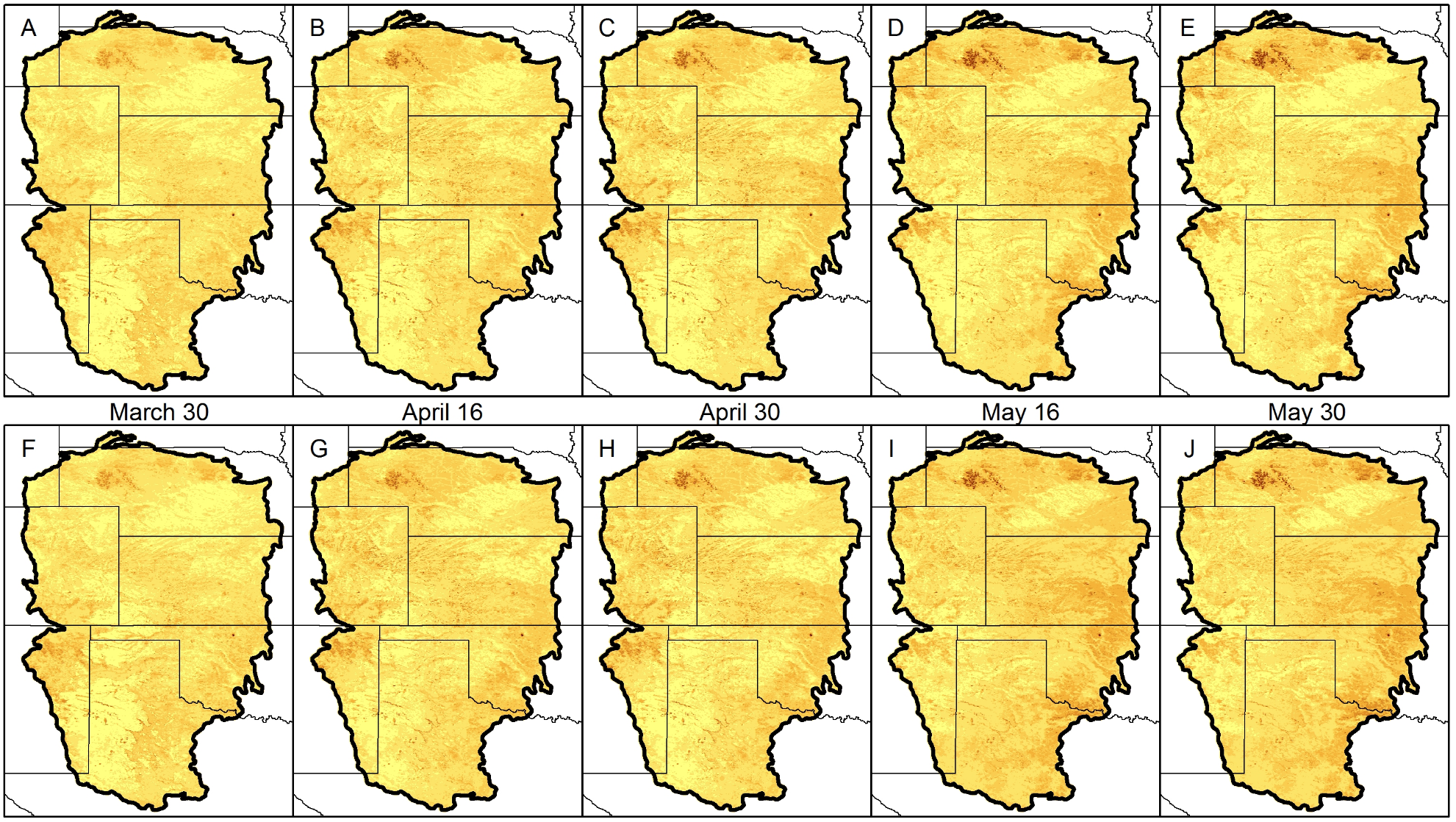


**Appendix 1b.** Probability of occurrence of American Avocet based on the ensemble of five general circulation models from CMIP5, Representative Concentration Pathway 8.5. The top row is based on 1981-2010 (hindcast) and the bottom row on 2041-2070 (forecast) climate data. The yellow-to-brown color ramp corresponds to small-to-large probability values.

**
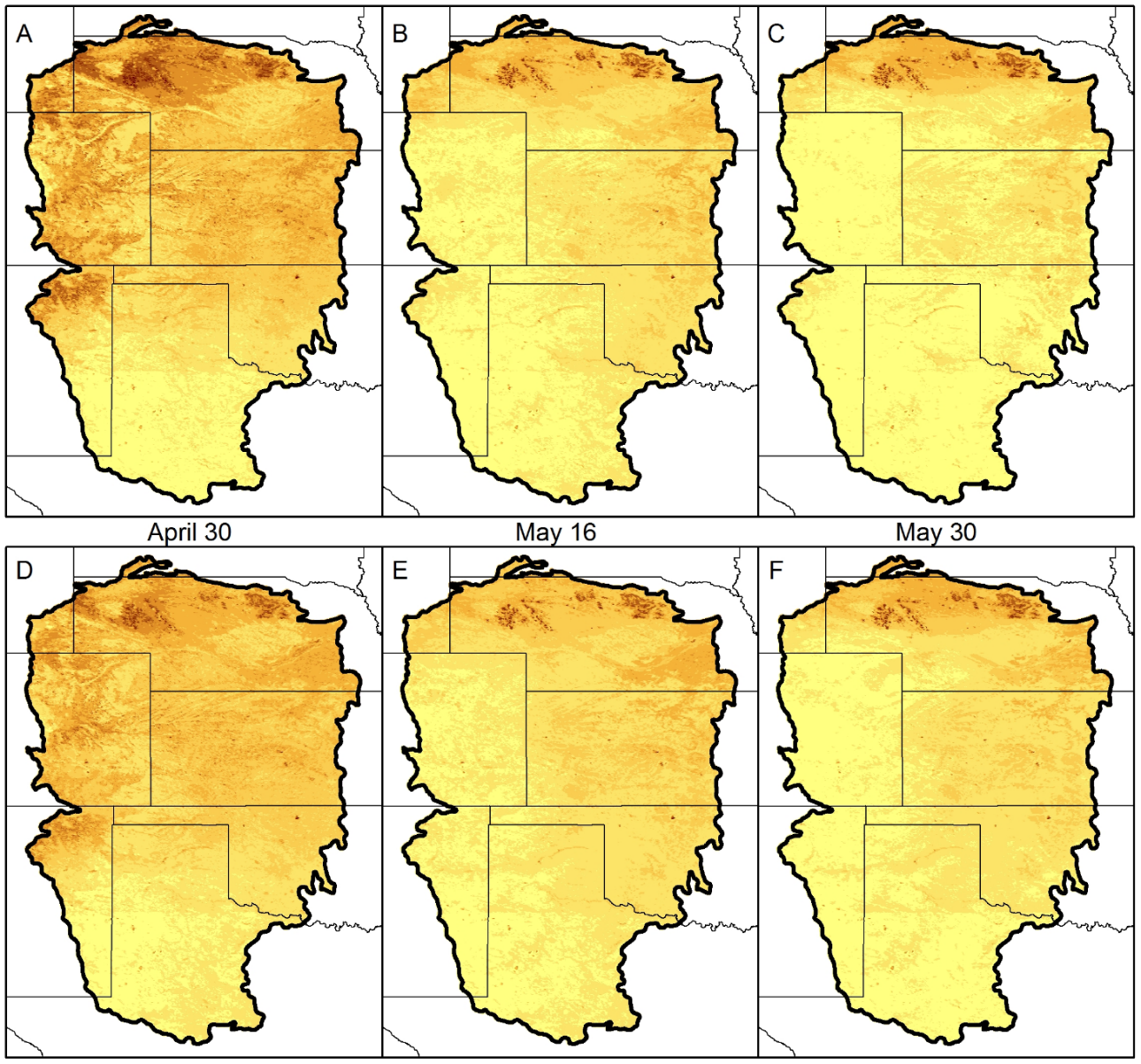
**

**Appendix 1c.** Probability of occurrence of Willet based on the ensemble of five general circulation models from CMIP5, Representative Concentration Pathway 8.5. The top row is based on 1981-2010 (hindcast) and the bottom row on 2041-2070 (forecast) climate data. The yellow-to-brown color ramp corresponds to small-to-large probability values.

**
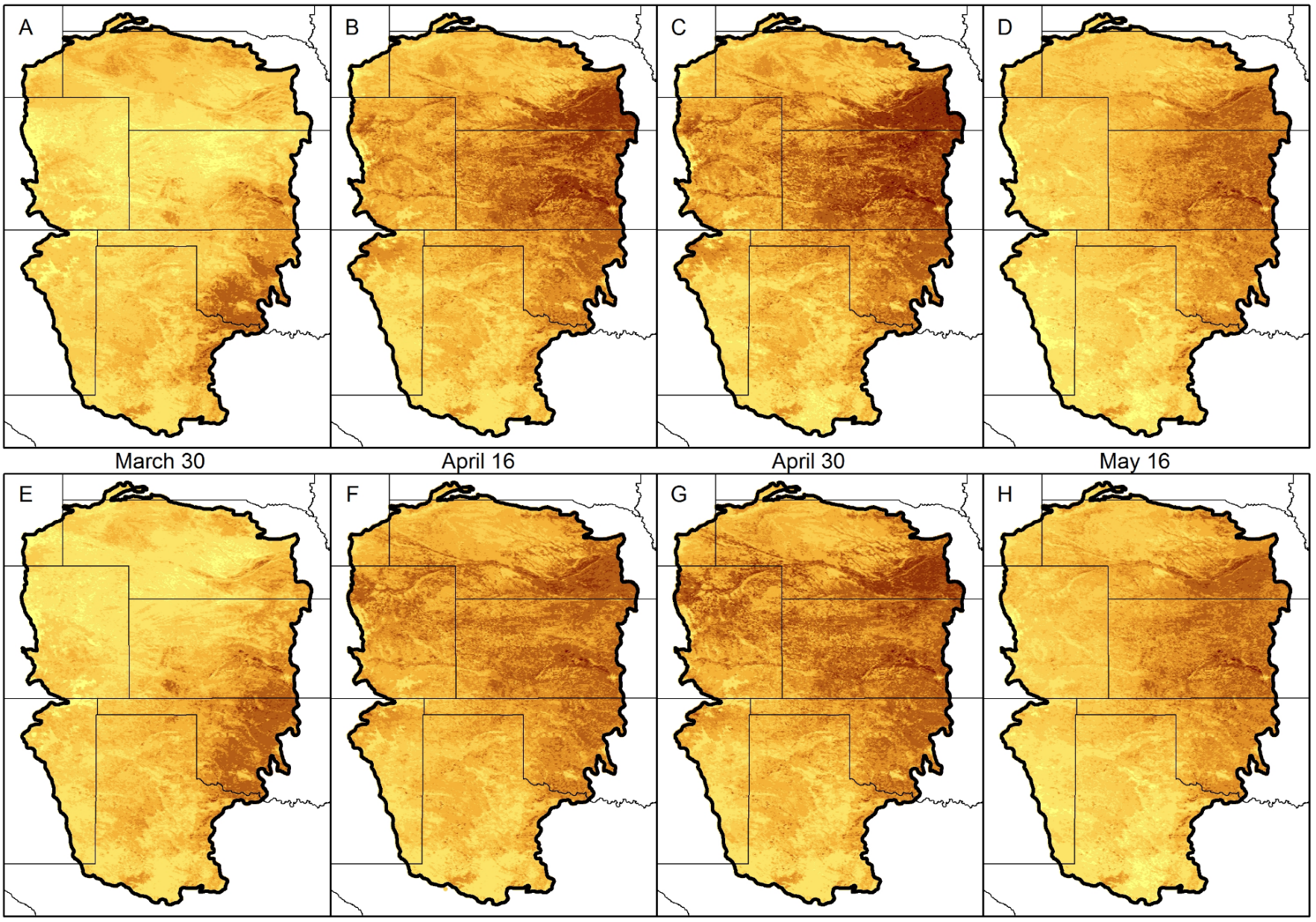
**

**Appendix 1d.** Probability of occurrence of Lesser Yellowlegs based on the ensemble of five general circulation models from CMIP5, Representative Concentration Pathway 8.5. The top row is based on 1981-2010 (hindcast) and the bottom row on 2041-2070 (forecast) climate data. The yellow-to-brown color ramp corresponds to small-to-large probability values.

**
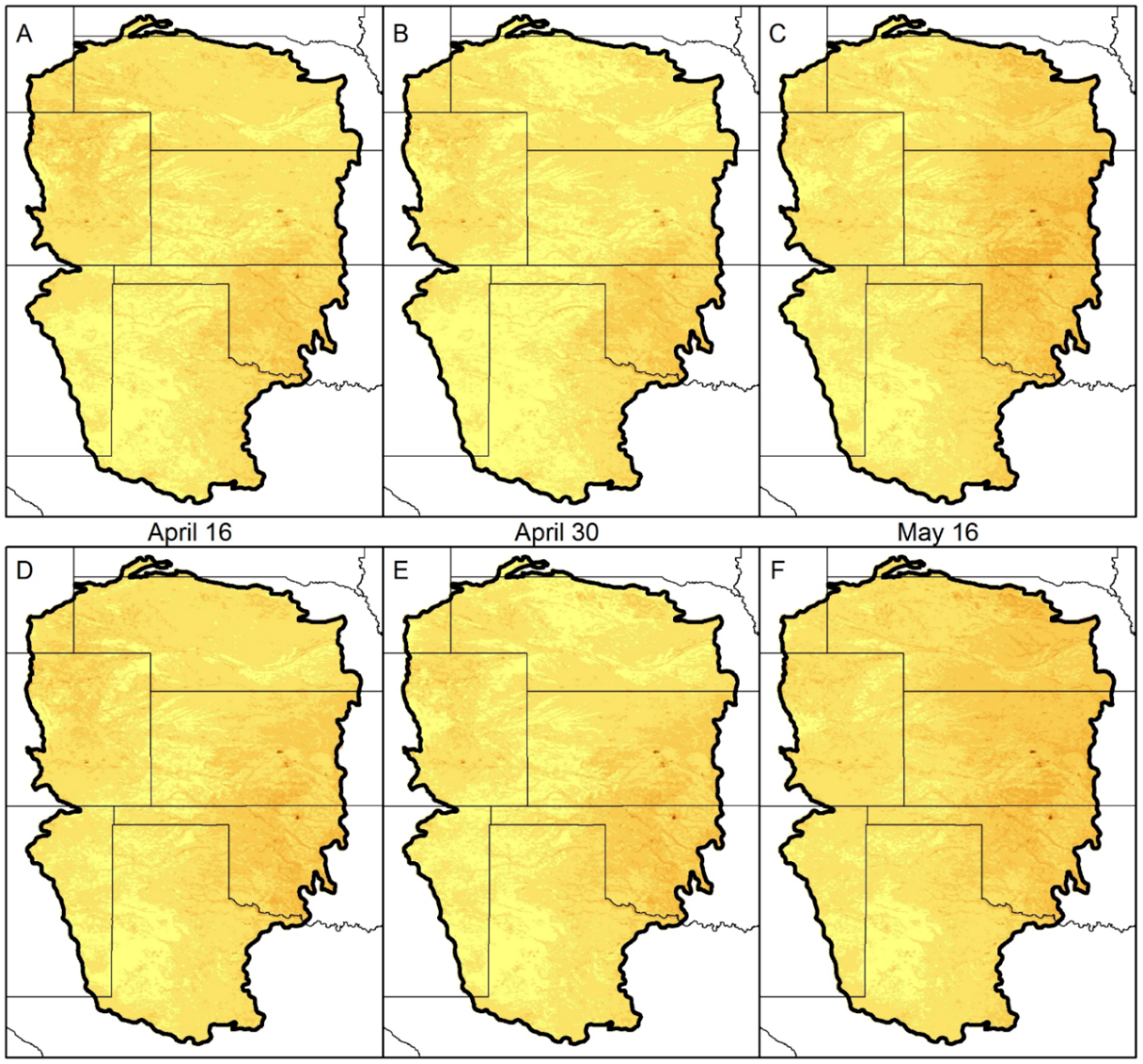
**

**Appendix 1e.** Probability of occurrence of Whimbrel based on the ensemble of five general circulation models from CMIP5, Representative Concentration Pathway 8.5. The top row is based on 1981-2010 (hindcast) and the bottom row on 2041-2070 (forecast) climate data. The yellow-to-brown color ramp corresponds to small-to-large probability values.

**
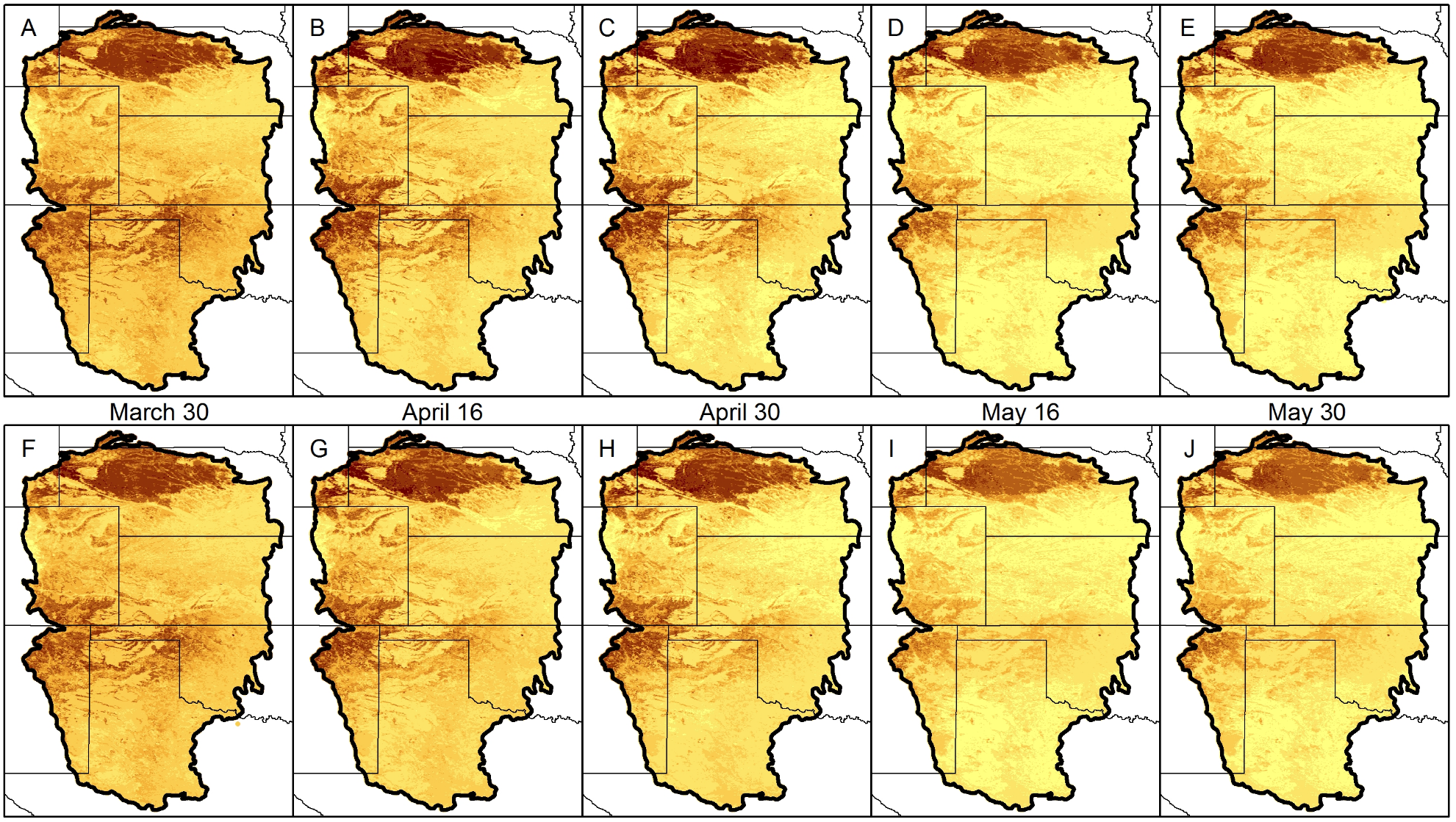
**

**Appendix 1f.** Probability of occurrence of Long-billed Curlew based on the ensemble of five general circulation models from CMIP5, Representative Concentration Pathway 8.5. The top row is based on 1981-2010 (hindcast) and the bottom row on 2041-2070 (forecast) climate data. The yellow-to-brown color ramp corresponds to small-to-large probability values.

**
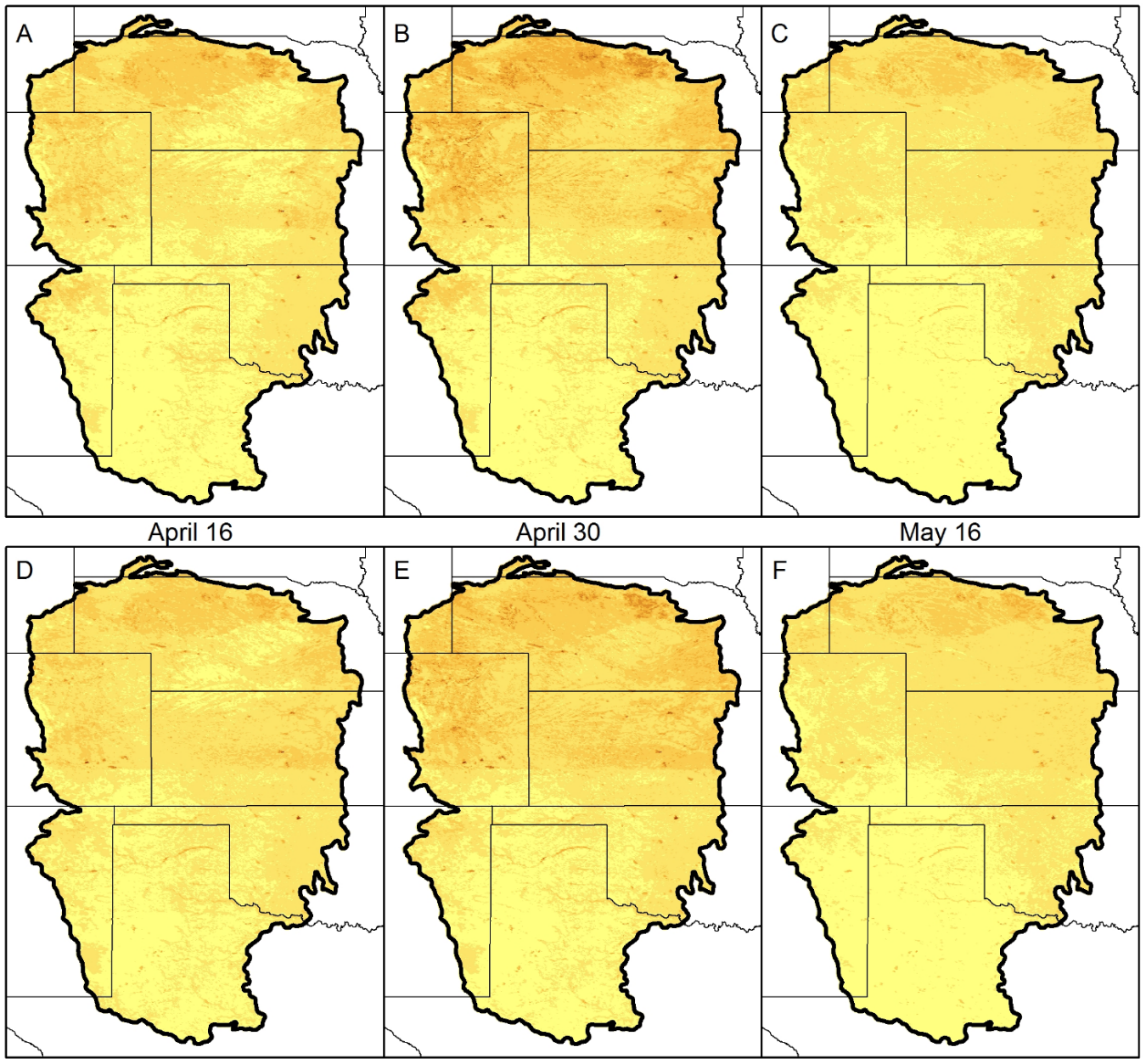
**

**Appendix 1g.** Probability of occurrence of Marbled Godwit based on the ensemble of five general circulation models from CMIP5, Representative Concentration Pathway 8.5. The top row is based on 1981-2010 (hindcast) and the bottom row on 2041-2070 (forecast) climate data. The yellow-to-brown color ramp corresponds to small-to-large probability values.

**
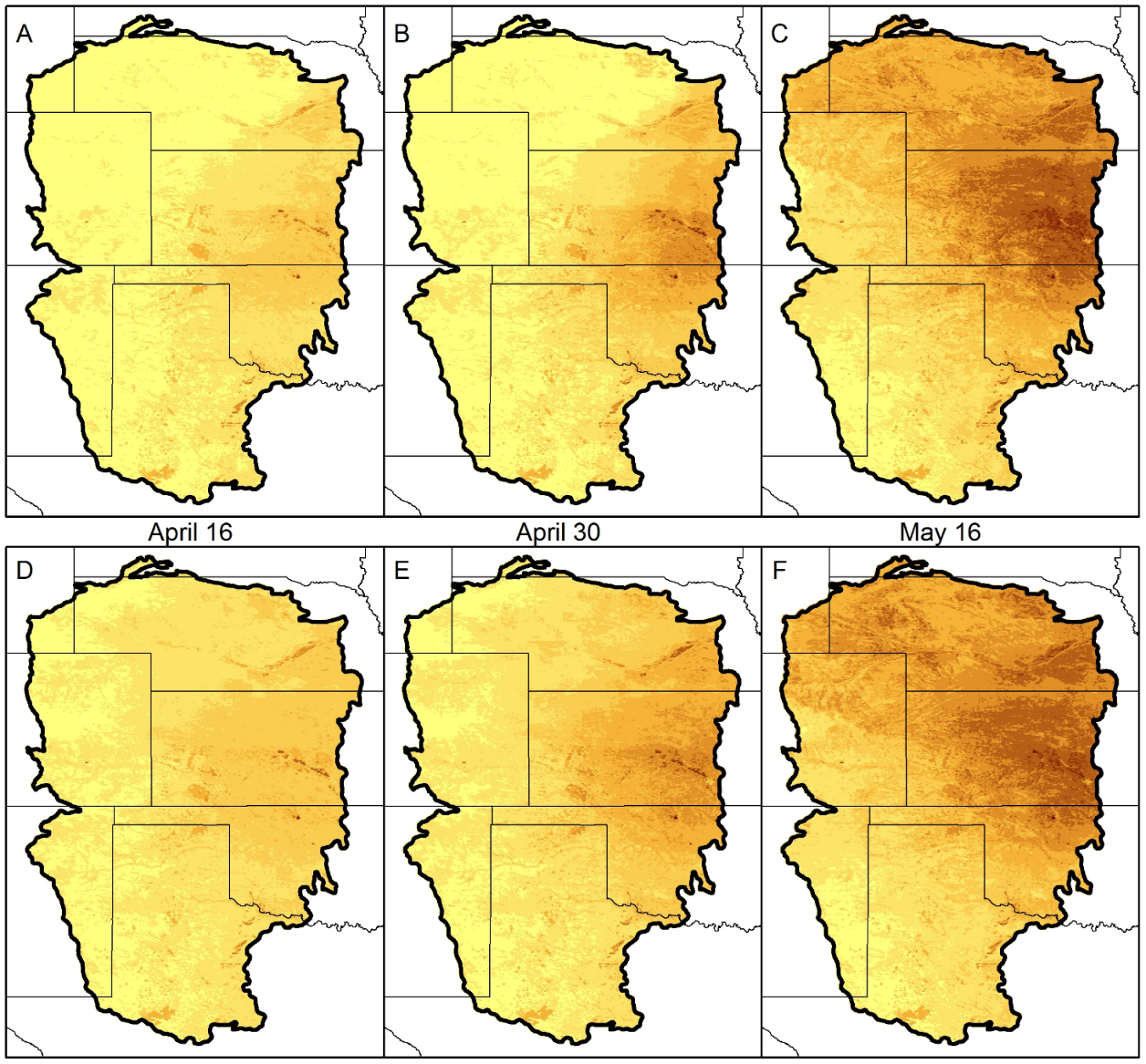
**

**Appendix 1h.** Probability of occurrence of Stilt Sandpiper based on the ensemble of five general circulation models from CMIP5, Representative Concentration Pathway 8.5. The top row is based on 1981-2010 (hindcast) and the bottom row on 2041-2070 (forecast) climate data. The yellow-to-brown color ramp corresponds to small-to-large probability values.

**
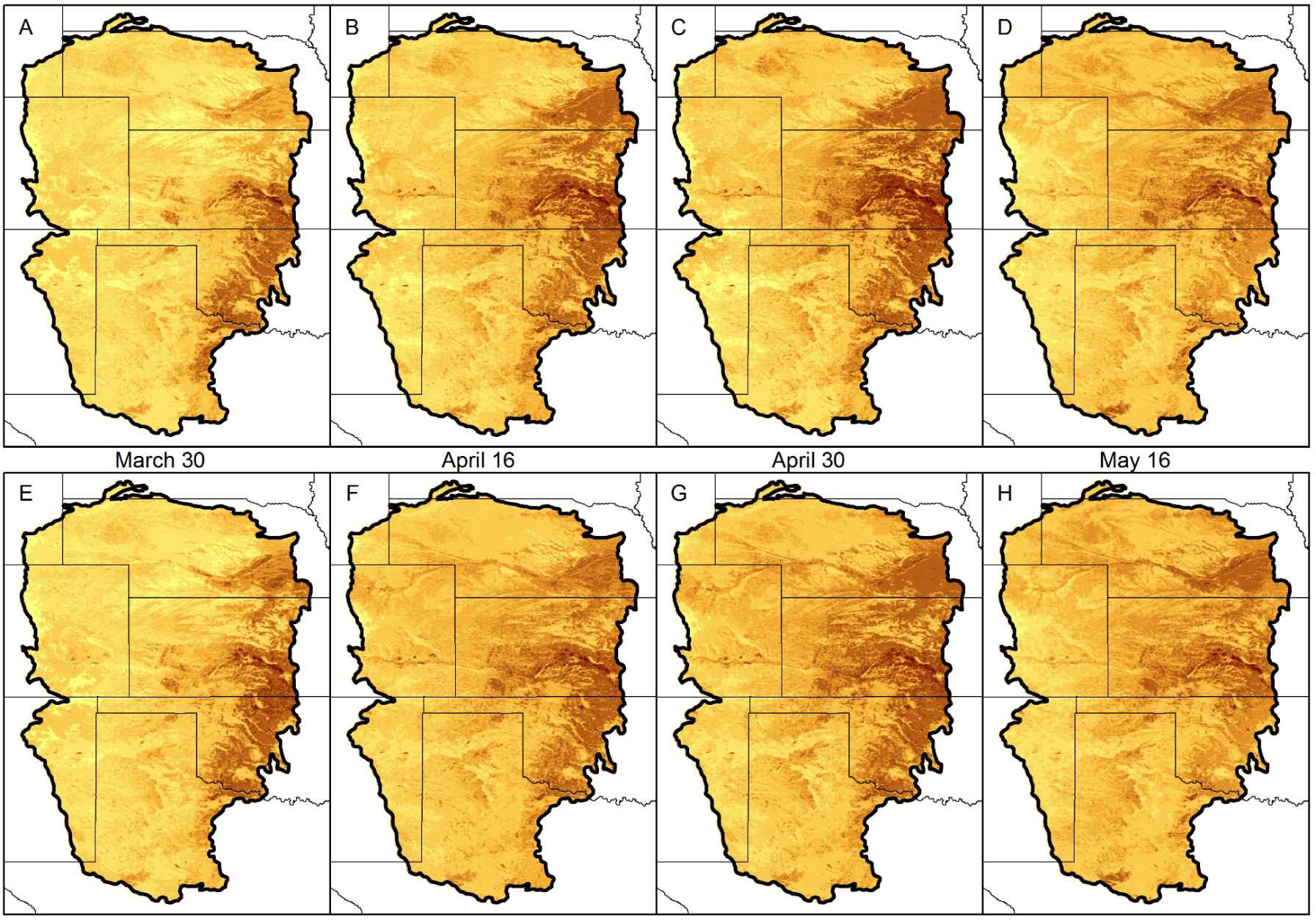
**

**Appendix 1i.** Probability of occurrence of Baird’s Sandpiper based on the ensemble of five general circulation models from CMIP5, Representative Concentration Pathway 8.5. The top row is based on 1981-2010 (hindcast) and the bottom row on 2041-2070 (forecast) climate data. The yellow-to-brown color ramp corresponds to small-to-large probability values.

**
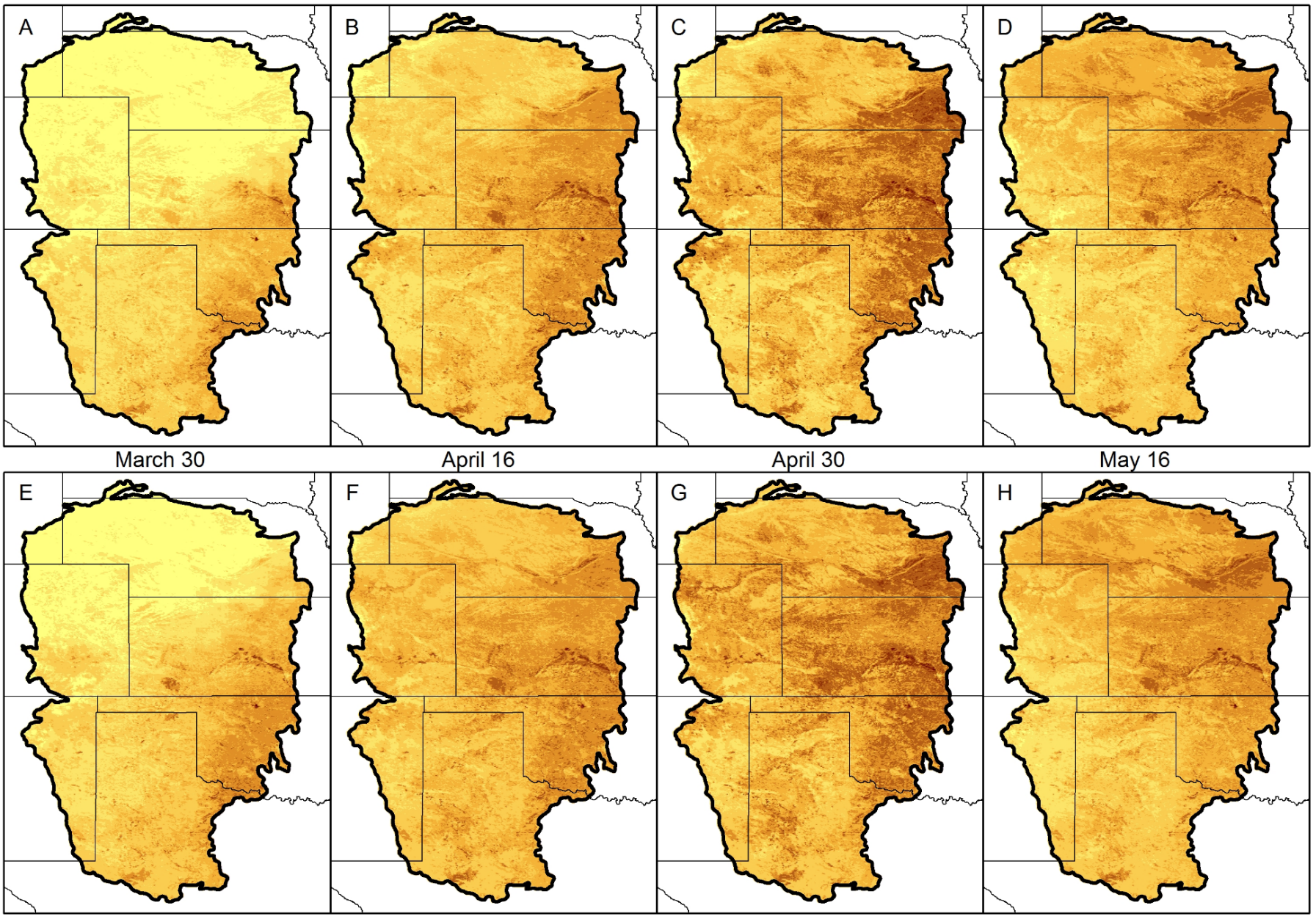
**

**Appendix 1j.** Probability of occurrence of Least Sandpiper based on the ensemble of five general circulation models from CMIP5, Representative Concentration Pathway 8.5. The top row is based on 1981-2010 (hindcast) and the bottom row on 2041-2070 (forecast) climate data. The yellow-to-brown color ramp corresponds to small-to-large probability values.

**
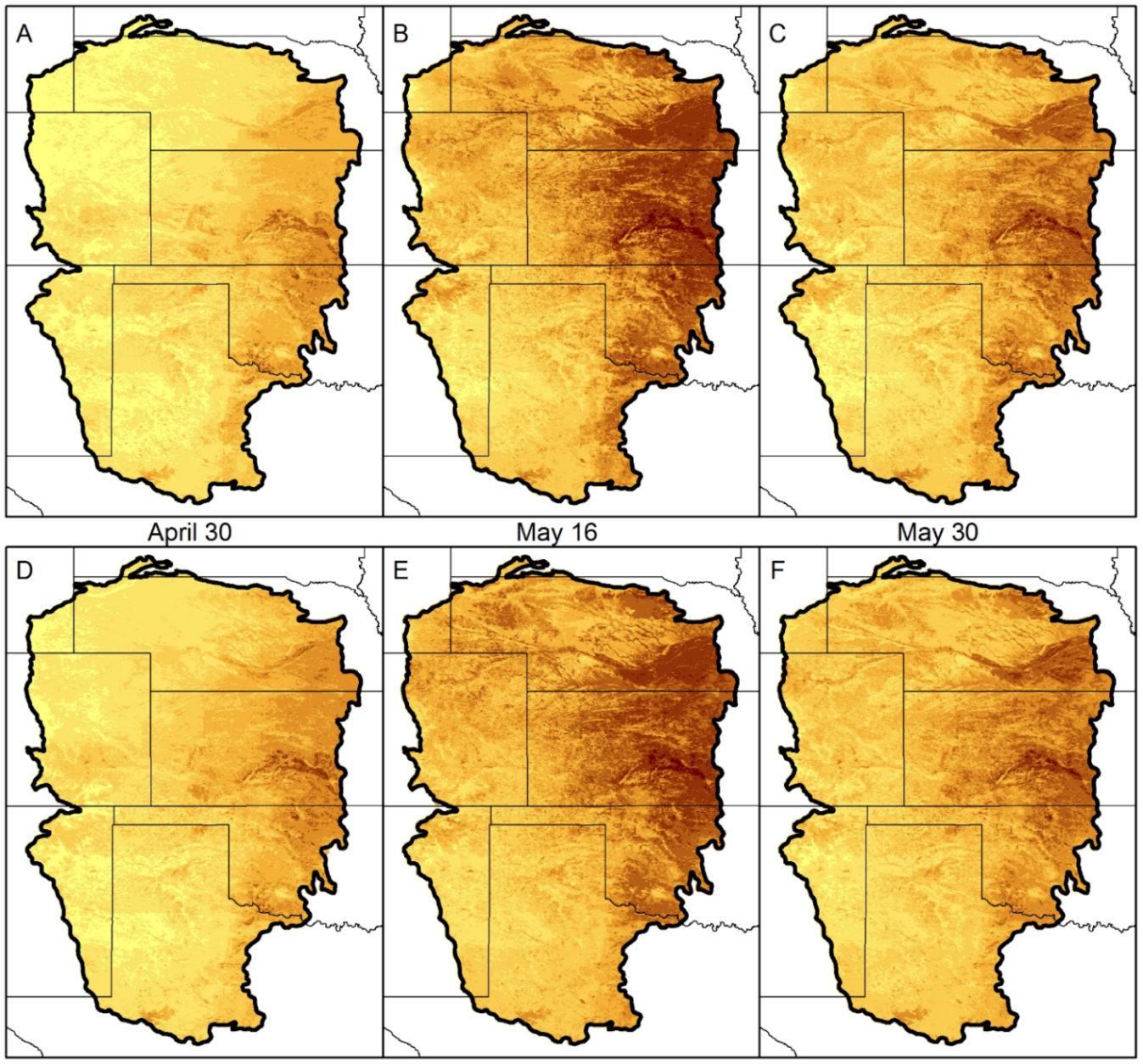
**

**Appendix 1k.** Probability of occurrence of White-rumped Sandpiper based on the ensemble of five general circulation models from CMIP5, Representative Concentration Pathway 8.5. The top row is based on 1981-2010 (hindcast) and the bottom row on 2041-2070 (forecast) climate data. The yellow-to-brown color ramp corresponds to small-to-large probability values.

**
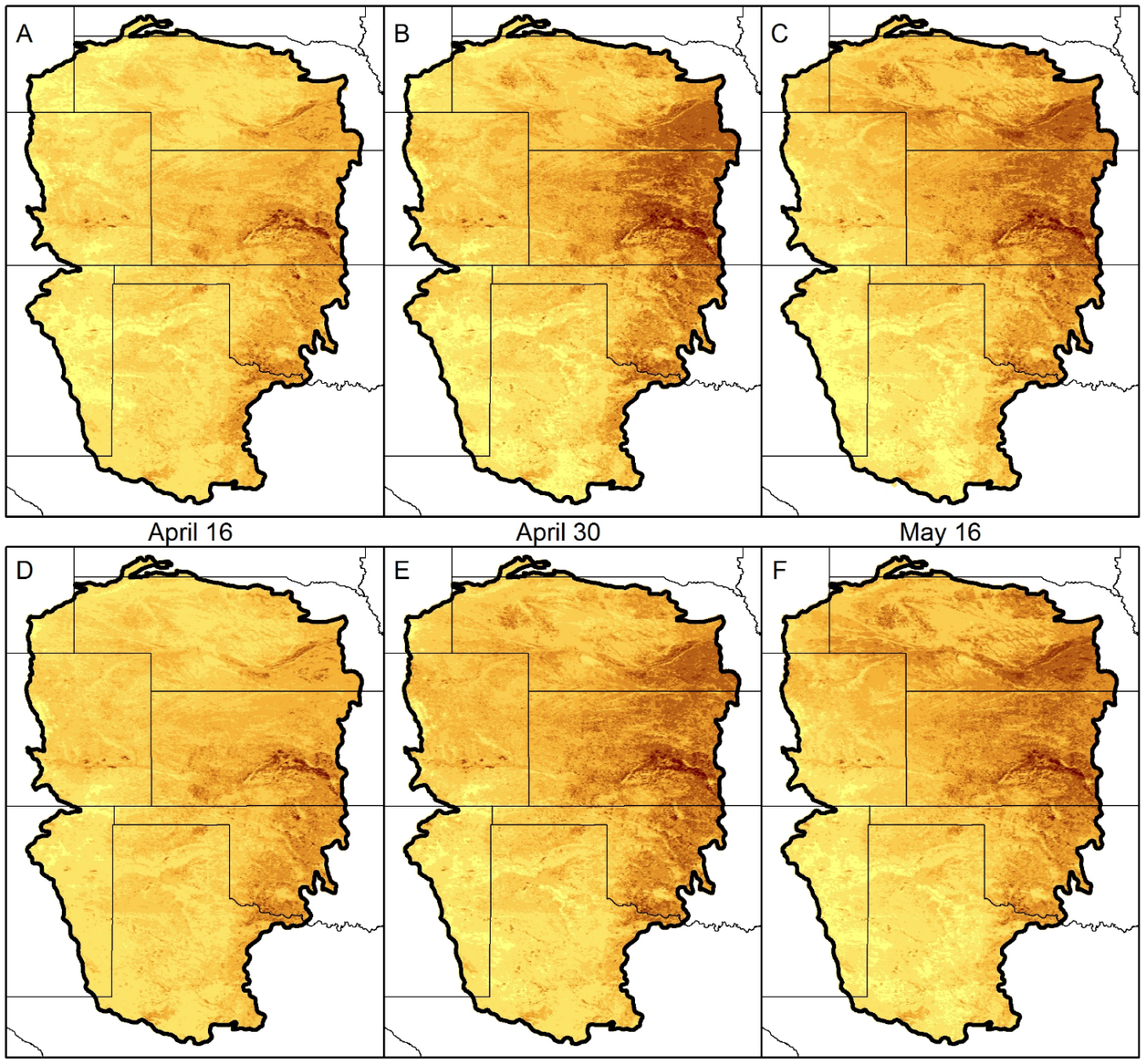
**

**Appendix 1l.** Probability of occurrence of Semipalmated Sandpiper based on the ensemble of five general circulation models from CMIP5, Representative Concentration Pathway 8.5. The top row is based on 1981-2010 (hindcast) and the bottom row on 2041-2070 (forecast) climate data. The yellow-to-brown color ramp corresponds to small-to-large probability values.

**
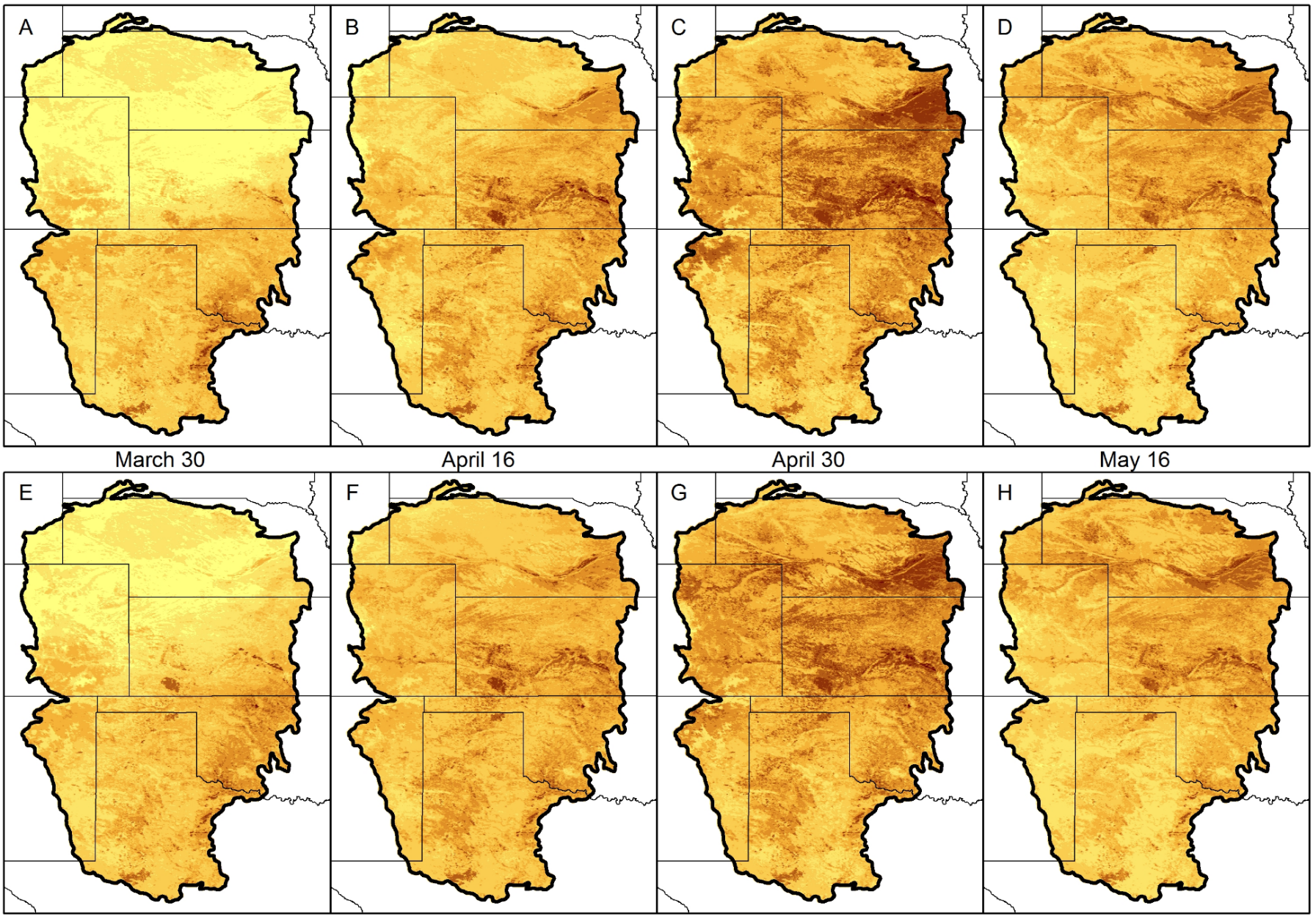
**

**Appendix 1m.** Probability of occurrence of Long-billed Dowitcher based on the ensemble of five general circulation models from CMIP5, Representative Concentration Pathway 8.5. The top row is based on 1981-2010 (hindcast) and the bottom row on 2041-2070 (forecast) climate data. The yellow-to-brown color ramp corresponds to small-to-large probability values.

**
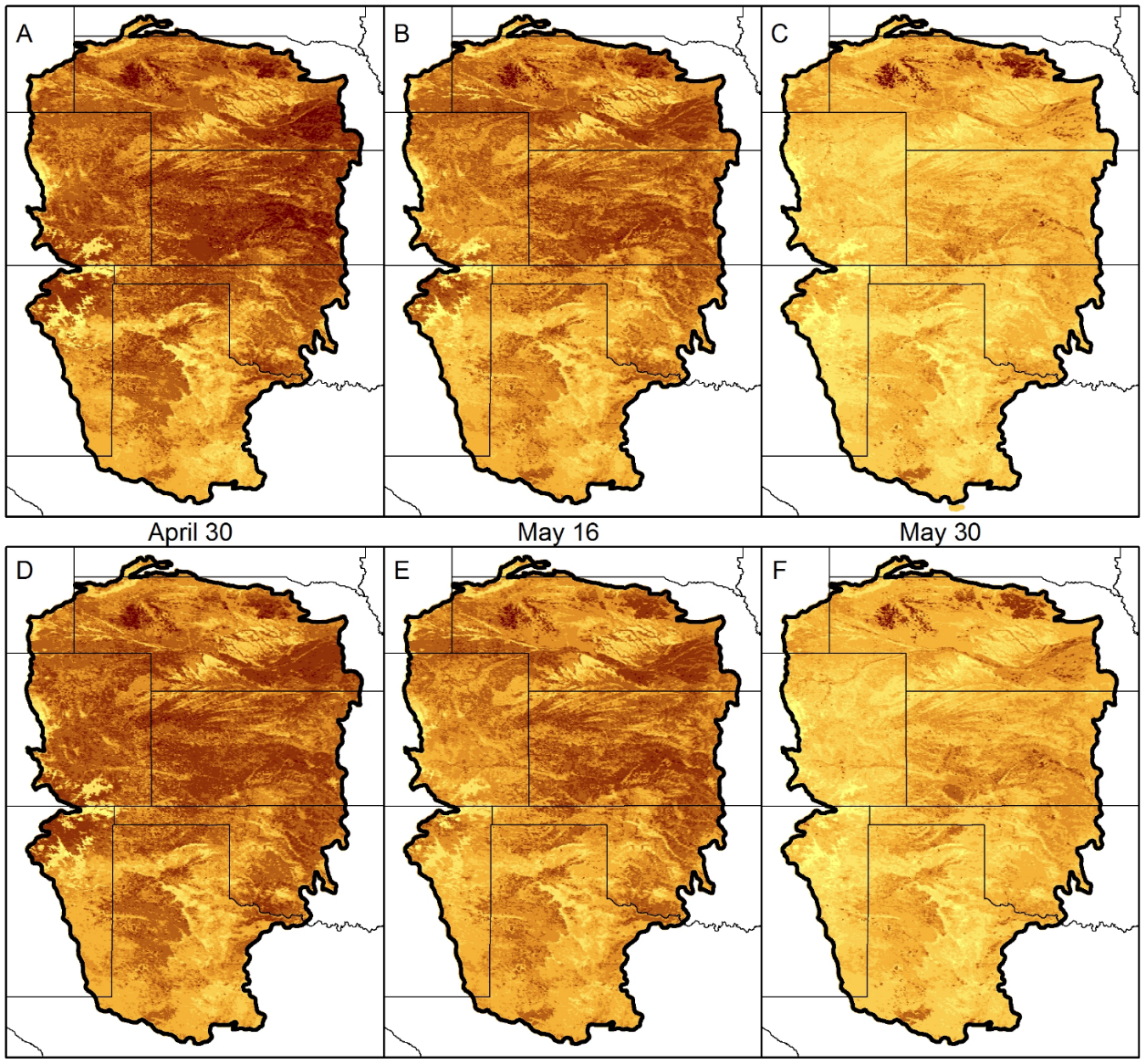
**

**Appendix 1n.** Probability of occurrence of Wilson’s Phalarope based on the ensemble of five general circulation models from CMIP5, Representative Concentration Pathway 8.5. The top row is based on 1981-2010 (hindcast) and the bottom row on 2041-2070 (forecast) climate data. The yellow-to-brown color ramp corresponds to small-to-large probability values.
