# Supplementary material for: Modeling nonbreeding distributions of shorebirds and waterfowl in response to climate change
Source: Ecol Evol. 2017 Feb 7;7(5):1497–513. doi: 10.1002/ece3.2755 (PMC5330909; doi:10.1002/ece3.2755)
Supplement: Supplementary file 2 [file ECE3-7-1497-s002.docx]

**Appendix 2**. Average values for probability of occurrence of 14 shorebird species during spring migration across the area delineated by the Great Plains Landscape Conservation Cooperative as projected by models incorporating climate predictions from five general circulation models from CMIP5. Values are provided for both hindcasts (contemporary) and forecasts (future).

|  |  | **Contemporary (1981-2010)** | | | | |  | **Future (2041-2070)** | | | | |
| --- | --- | --- | --- | --- | --- | --- | --- | --- | --- | --- | --- | --- |
|  | **General Circulation Model** | **Mar 30** | **Apr 16** | **Apr 30** | **May 16** | **May 30** |  | **Mar 30** | **Apr 16** | **Apr 30** | **May 16** | **May 30** |
| Mountain Plover | Access1-0 |  | 0.29 | 0.25 | 0.18 | 0.20 |  |  | 0.28 | 0.24 | 0.19 | 0.20 |
|  | CMCC-CM |  | 0.29 | 0.26 | 0.19 | 0.20 |  |  | 0.27 | 0.23 | 0.18 | 0.19 |
|  | GFDL-CM3 |  | 0.29 | 0.26 | 0.19 | 0.20 |  |  | 0.25 | 0.22 | 0.17 | 0.18 |
|  | inmcm4 |  | 0.29 | 0.26 | 0.19 | 0.20 |  |  | 0.29 | 0.25 | 0.20 | 0.21 |
|  | IPSL-CM5B-LR |  | 0.30 | 0.26 | 0.19 | 0.20 |  |  | 0.26 | 0.23 | 0.18 | 0.19 |
| American Avocet | Access1-0 | 0.19 | 0.20 | 0.21 | 0.22 | 0.21 |  | 0.19 | 0.22 | 0.22 | 0.26 | 0.26 |
|  | CMCC-CM | 0.19 | 0.21 | 0.21 | 0.22 | 0.21 |  | 0.19 | 0.22 | 0.21 | 0.25 | 0.24 |
|  | GFDL-CM3 | 0.19 | 0.20 | 0.21 | 0.22 | 0.21 |  | 0.20 | 0.21 | 0.21 | 0.26 | 0.26 |
|  | inmcm4 | 0.19 | 0.20 | 0.21 | 0.22 | 0.21 |  | 0.19 | 0.21 | 0.21 | 0.24 | 0.24 |
|  | IPSL-CM5B-LR | 0.20 | 0.21 | 0.21 | 0.21 | 0.20 |  | 0.19 | 0.21 | 0.21 | 0.24 | 0.23 |
| Willet | Access1-0 |  |  | 0.30 | 0.18 | 0.14 |  |  |  | 0.28 | 0.21 | 0.17 |
|  | CMCC-CM |  |  | 0.31 | 0.18 | 0.14 |  |  |  | 0.29 | 0.2 | 0.17 |
|  | GFDL-CM3 |  |  | 0.30 | 0.18 | 0.14 |  |  |  | 0.28 | 0.22 | 0.18 |
|  | inmcm4 |  |  | 0.30 | 0.18 | 0.14 |  |  |  | 0.28 | 0.19 | 0.16 |
|  | IPSL-CM5B-LR |  |  | 0.31 | 0.17 | 0.13 |  |  |  | 0.29 | 0.20 | 0.16 |
| Lesser Yellowlegs | Access1-0 | 0.30 | 0.46 | 0.47 | 0.38 |  |  | 0.32 | 0.46 | 0.48 | 0.38 |  |
|  | CMCC-CM | 0.30 | 0.47 | 0.48 | 0.38 |  |  | 0.32 | 0.47 | 0.49 | 0.39 |  |
|  | GFDL-CM3 | 0.31 | 0.46 | 0.47 | 0.38 |  |  | 0.36 | 0.48 | 0.49 | 0.41 |  |
|  | inmcm4 | 0.30 | 0.45 | 0.47 | 0.38 |  |  | 0.31 | 0.45 | 0.47 | 0.37 |  |
|  | IPSL-CM5B-LR | 0.31 | 0.47 | 0.49 | 0.38 |  |  | 0.35 | 0.47 | 0.49 | 0.39 |  |
| Whimbrel | Access1-0 |  | 0.17 | 0.15 | 0.21 |  |  |  | 0.19 | 0.18 | 0.21 |  |
|  | CMCC-CM |  | 0.18 | 0.16 | 0.21 |  |  |  | 0.19 | 0.18 | 0.22 |  |
|  | GFDL-CM3 |  | 0.17 | 0.15 | 0.21 |  |  |  | 0.19 | 0.18 | 0.23 |  |
|  | inmcm4 |  | 0.18 | 0.15 | 0.20 |  |  |  | 0.18 | 0.16 | 0.20 |  |
|  | IPSL-CM5B-LR |  | 0.18 | 0.16 | 0.20 |  |  |  | 0.19 | 0.18 | 0.23 |  |
| Long-billed Curlew | Access1-0 | 0.40 | 0.36 | 0.31 | 0.23 | 0.23 |  | 0.40 | 0.36 | 0.31 | 0.23 | 0.24 |
|  | CMCC-CM | 0.40 | 0.36 | 0.31 | 0.23 | 0.24 |  | 0.40 | 0.35 | 0.31 | 0.23 | 0.23 |
|  | GFDL-CM3 | 0.41 | 0.36 | 0.31 | 0.23 | 0.24 |  | 0.40 | 0.35 | 0.30 | 0.22 | 0.23 |
|  | inmcm4 | 0.41 | 0.36 | 0.31 | 0.23 | 0.24 |  | 0.40 | 0.35 | 0.31 | 0.23 | 0.24 |
|  | IPSL-CM5B-LR | 0.42 | 0.36 | 0.32 | 0.23 | 0.24 |  | 0.40 | 0.35 | 0.31 | 0.23 | 0.24 |
| Marbled Godwit | Access1-0 |  | 0.16 | 0.20 | 0.14 |  |  |  | 0.17 | 0.19 | 0.14 |  |
|  | CMCC-CM |  | 0.16 | 0.20 | 0.13 |  |  |  | 0.17 | 0.20 | 0.14 |  |
|  | GFDL-CM3 |  | 0.16 | 0.20 | 0.13 |  |  |  | 0.17 | 0.20 | 0.15 |  |
|  | inmcm4 |  | 0.16 | 0.20 | 0.13 |  |  |  | 0.16 | 0.19 | 0.13 |  |
|  | IPSL-CM5B-LR |  | 0.16 | 0.21 | 0.14 |  |  |  | 0.17 | 0.19 | 0.14 |  |
| Stilt Sandpiper | Access1-0 |  | 0.14 | 0.19 | 0.38 |  |  |  | 0.21 | 0.25 | 0.39 |  |
|  | CMCC-CM |  | 0.15 | 0.19 | 0.38 |  |  |  | 0.19 | 0.24 | 0.39 |  |
|  | GFDL-CM3 |  | 0.14 | 0.18 | 0.38 |  |  |  | 0.22 | 0.28 | 0.40 |  |
|  | inmcm4 |  | 0.14 | 0.18 | 0.37 |  |  |  | 0.17 | 0.21 | 0.37 |  |
|  | IPSL-CM5B-LR |  | 0.14 | 0.18 | 0.37 |  |  |  | 0.20 | 0.26 | 0.40 |  |
| Baird's Sandpiper | Access1-0 | 0.29 | 0.37 | 0.39 | 0.38 |  |  | 0.30 | 0.40 | 0.41 | 0.41 |  |
|  | CMCC-CM | 0.29 | 0.37 | 0.39 | 0.39 |  |  | 0.31 | 0.40 | 0.42 | 0.41 |  |
|  | GFDL-CM3 | 0.30 | 0.37 | 0.39 | 0.38 |  |  | 0.35 | 0.42 | 0.44 | 0.41 |  |
|  | inmcm4 | 0.29 | 0.36 | 0.38 | 0.38 |  |  | 0.28 | 0.37 | 0.39 | 0.40 |  |
|  | IPSL-CM5B-LR | 0.30 | 0.37 | 0.39 | 0.37 |  |  | 0.34 | 0.41 | 0.43 | 0.40 |  |
| Least Sandpiper | Access1-0 | 0.20 | 0.37 | 0.43 | 0.39 |  |  | 0.25 | 0.42 | 0.46 | 0.40 |  |
|  | CMCC-CM | 0.20 | 0.38 | 0.44 | 0.4 |  |  | 0.25 | 0.41 | 0.46 | 0.40 |  |
|  | GFDL-CM3 | 0.20 | 0.37 | 0.43 | 0.39 |  |  | 0.30 | 0.43 | 0.47 | 0.41 |  |
|  | inmcm4 | 0.21 | 0.36 | 0.42 | 0.39 |  |  | 0.23 | 0.39 | 0.44 | 0.39 |  |
|  | IPSL-CM5B-LR | 0.21 | 0.38 | 0.44 | 0.38 |  |  | 0.28 | 0.42 | 0.47 | 0.41 |  |
| White-rumped Sandpiper | Access1-0 |  |  | 0.24 | 0.46 | 0.38 |  |  |  | 0.28 | 0.46 | 0.39 |
|  | CMCC-CM |  |  | 0.25 | 0.47 | 0.38 |  |  |  | 0.28 | 0.47 | 0.39 |
|  | GFDL-CM3 |  |  | 0.23 | 0.46 | 0.38 |  |  |  | 0.31 | 0.49 | 0.41 |
|  | inmcm4 |  |  | 0.23 | 0.45 | 0.37 |  |  |  | 0.25 | 0.45 | 0.37 |
|  | IPSL-CM5B-LR |  |  | 0.23 | 0.45 | 0.37 |  |  |  | 0.30 | 0.48 | 0.40 |
| Semipalmated Sandpiper | Access1-0 |  | 0.29 | 0.35 | 0.36 |  |  |  | 0.32 | 0.36 | 0.36 |  |
|  | CMCC-CM |  | 0.3 | 0.35 | 0.36 |  |  |  | 0.32 | 0.37 | 0.36 |  |
|  | GFDL-CM3 |  | 0.29 | 0.35 | 0.35 |  |  |  | 0.34 | 0.39 | 0.37 |  |
|  | inmcm4 |  | 0.29 | 0.34 | 0.35 |  |  |  | 0.3 | 0.35 | 0.34 |  |
|  | IPSL-CM5B-LR |  | 0.29 | 0.35 | 0.35 |  |  |  | 0.33 | 0.38 | 0.37 |  |
| Long-billed Dowitcher | Access1-0 | 0.23 | 0.37 | 0.47 | 0.38 |  |  | 0.26 | 0.41 | 0.47 | 0.38 |  |
|  | CMCC-CM | 0.23 | 0.38 | 0.48 | 0.38 |  |  | 0.26 | 0.40 | 0.48 | 0.38 |  |
|  | GFDL-CM3 | 0.23 | 0.36 | 0.47 | 0.38 |  |  | 0.3 | 0.41 | 0.48 | 0.38 |  |
|  | inmcm4 | 0.24 | 0.36 | 0.46 | 0.38 |  |  | 0.25 | 0.38 | 0.46 | 0.38 |  |
|  | IPSL-CM5B-LR | 0.24 | 0.37 | 0.48 | 0.38 |  |  | 0.29 | 0.40 | 0.48 | 0.38 |  |
| Wilson's Phalarope | Access1-0 |  |  | 0.62 | 0.55 | 0.37 |  |  |  | 0.6 | 0.55 | 0.39 |
|  | CMCC-CM |  |  | 0.62 | 0.56 | 0.37 |  |  |  | 0.61 | 0.55 | 0.39 |
|  | GFDL-CM3 |  |  | 0.61 | 0.55 | 0.37 |  |  |  | 0.61 | 0.55 | 0.40 |
|  | inmcm4 |  |  | 0.61 | 0.55 | 0.37 |  |  |  | 0.59 | 0.55 | 0.38 |
|  | IPSL-CM5B-LR |  |  | 0.62 | 0.55 | 0.36 |  |  |  | 0.61 | 0.55 | 0.39 |

**Appendix 3**. Projected changes in probability of occurrence for shorebirds for individual general circulation model (forecast – hindcast) and the average change across time periods.

|  |  | Differences | |  |  |  |  |  |
| --- | --- | --- | --- | --- | --- | --- | --- | --- |
|  | **General Circulation Model** | **Mar 30** | **Apr 16** | **Apr 30** | **May 16** | **May 30** | **Average change** | |
| **American avocet** | Access1-0 | 0.009 | 0.022 | 0.016 | 0.048 | 0.051 | 0.029 |  |
|  | CMCC-CM | -0.001 | 0.009 | 0.004 | 0.026 | 0.030 | 0.014 |  |
|  | GFDL-CM3 | 0.001 | 0.011 | 0.003 | 0.040 | 0.047 | 0.020 |  |
|  | inmcm4 | 0.003 | 0.011 | 0.007 | 0.029 | 0.030 | 0.016 |  |
|  | IPSL-CM5B-LR | -0.005 | 0.002 | -0.003 | 0.023 | 0.029 | 0.009 | 0.018 |
| **Baird's sandpiper** | Access1-0 | 0.005 | 0.029 | 0.023 | 0.033 |  | 0.022 |  |
|  | CMCC-CM | 0.016 | 0.027 | 0.024 | 0.022 |  | 0.022 |  |
|  | GFDL-CM3 | 0.058 | 0.055 | 0.054 | 0.031 |  | 0.050 |  |
|  | inmcm4 | -0.005 | 0.015 | 0.011 | 0.020 |  | 0.010 |  |
|  | IPSL-CM5B-LR | 0.044 | 0.044 | 0.044 | 0.033 |  | 0.041 | 0.029 |
| **Least sandpiper** | Access1-0 | 0.042 | 0.048 | 0.032 | 0.009 |  | 0.033 |  |
|  | CMCC-CM | 0.047 | 0.031 | 0.020 | 0.008 |  | 0.026 |  |
|  | GFDL-CM3 | 0.094 | 0.063 | 0.047 | 0.020 |  | 0.056 |  |
|  | inmcm4 | 0.027 | 0.024 | 0.014 | 0.004 |  | 0.017 |  |
|  | IPSL-CM5B-LR | 0.070 | 0.045 | 0.032 | 0.023 |  | 0.043 | 0.035 |
| **Lesser yellowlegs** | Access1-0 | 0.017 | 0.007 | 0.004 | 0.000 |  | 0.007 |  |
|  | CMCC-CM | 0.018 | 0.007 | 0.007 | 0.006 |  | 0.010 |  |
|  | GFDL-CM3 | 0.055 | 0.022 | 0.019 | 0.031 |  | 0.032 |  |
|  | inmcm4 | 0.008 | -0.004 | -0.005 | -0.005 |  | -0.002 |  |
|  | IPSL-CM5B-LR | 0.044 | 0.003 | 0.000 | 0.017 |  | 0.016 | 0.013 |
| **Long-billed curlew** | Access1-0 | -0.003 | -0.004 | -0.006 | 0.007 | 0.006 | 0.000 |  |
|  | CMCC-CM | -0.002 | -0.005 | -0.009 | -0.004 | -0.005 | -0.005 |  |
|  | GFDL-CM3 | -0.011 | -0.010 | -0.012 | -0.009 | -0.011 | -0.011 |  |
|  | inmcm4 | -0.006 | -0.003 | -0.006 | 0.004 | 0.004 | -0.001 |  |
|  | IPSL-CM5B-LR | -0.017 | -0.012 | -0.014 | -0.001 | -0.003 | -0.010 | -0.005 |
| **Long-billed** | Access1-0 | 0.033 | 0.042 | 0.003 | 0.002 |  | 0.020 |  |
| **dowitcher** | CMCC-CM | 0.029 | 0.025 | -0.001 | -0.004 |  | 0.013 |  |
|  | GFDL-CM3 | 0.067 | 0.043 | 0.010 | 0.004 |  | 0.031 |  |
|  | inmcm4 | 0.017 | 0.019 | -0.006 | -0.001 |  | 0.007 |  |
|  | IPSL-CM5B-LR | 0.048 | 0.028 | -0.004 | 0.007 |  | 0.020 | 0.018 |
| **Marbled godwit** | Access1-0 |  | 0.008 | -0.007 | -0.001 |  | 0.000 |  |
|  | CMCC-CM |  | 0.010 | 0.003 | 0.002 |  | 0.005 |  |
|  | GFDL-CM3 |  | 0.013 | 0.001 | 0.013 |  | 0.009 |  |
|  | inmcm4 |  | 0.001 | -0.009 | -0.003 |  | -0.004 |  |
|  | IPSL-CM5B-LR |  | 0.002 | -0.012 | 0.002 |  | -0.003 | 0.002 |
| **Mountain plover** | Access1-0 |  | -0.010 | -0.012 | 0.009 | 0.009 | -0.001 |  |
|  | CMCC-CM |  | -0.026 | -0.027 | -0.010 | -0.010 | -0.018 |  |
|  | GFDL-CM3 |  | -0.042 | -0.041 | -0.017 | -0.018 | -0.029 |  |
|  | inmcm4 |  | -0.007 | -0.009 | 0.005 | 0.005 | -0.002 |  |
|  | IPSL-CM5B-LR |  | -0.037 | -0.035 | -0.013 | -0.014 | -0.025 | -0.015 |
| **Semi-palmated** | Access1-0 |  | 0.029 | 0.014 | -0.001 |  | 0.014 |  |
| **sandpiper** | CMCC-CM |  | 0.024 | 0.019 | 0.005 |  | 0.016 |  |
|  | GFDL-CM3 |  | 0.047 | 0.040 | 0.021 |  | 0.036 |  |
|  | inmcm4 |  | 0.014 | 0.004 | -0.006 |  | 0.004 |  |
|  | IPSL-CM5B-LR |  | 0.039 | 0.032 | 0.022 |  | 0.031 | 0.020 |
| **Stilt sandpiper** | Access1-0 |  | 0.065 | 0.066 | 0.009 |  | 0.047 |  |
|  | CMCC-CM |  | 0.044 | 0.050 | 0.012 |  | 0.036 |  |
|  | GFDL-CM3 |  | 0.079 | 0.098 | 0.027 |  | 0.068 |  |
|  | inmcm4 |  | 0.030 | 0.029 | 0.002 |  | 0.020 |  |
|  | IPSL-CM5B-LR |  | 0.063 | 0.077 | 0.028 |  | 0.056 | 0.045 |
| **Whimbrel** | Access1-0 |  | 0.017 | 0.023 | 0.006 |  | 0.015 |  |
|  | CMCC-CM |  | 0.009 | 0.013 | 0.009 |  | 0.011 |  |
|  | GFDL-CM3 |  | 0.022 | 0.030 | 0.027 |  | 0.026 |  |
|  | inmcm4 |  | 0.005 | 0.008 | 0.001 |  | 0.004 |  |
|  | IPSL-CM5B-LR |  | 0.014 | 0.020 | 0.028 |  | 0.021 | 0.016 |
| **White-rumped** | Access1-0 |  |  | 0.045 | -0.005 | 0.005 | 0.015 |  |
| **sandpiper** | CMCC-CM |  |  | 0.035 | 0.000 | 0.006 | 0.013 |  |
|  | GFDL-CM3 |  |  | 0.081 | 0.032 | 0.038 | 0.050 |  |
|  | inmcm4 |  |  | 0.015 | -0.009 | -0.003 | 0.001 |  |
|  | IPSL-CM5B-LR |  |  | 0.068 | 0.030 | 0.031 | 0.043 | 0.025 |
| **Willet** | Access1-0 |  |  | -0.018 | 0.025 | 0.033 | 0.013 |  |
|  | CMCC-CM |  |  | -0.015 | 0.019 | 0.024 | 0.010 |  |
|  | GFDL-CM3 |  |  | -0.017 | 0.035 | 0.040 | 0.019 |  |
|  | inmcm4 |  |  | -0.019 | 0.013 | 0.018 | 0.004 |  |
|  | IPSL-CM5B-LR |  |  | -0.020 | 0.024 | 0.028 | 0.010 | 0.011 |
| **Wilson's phalarope** | Access1-0 |  |  | -0.018 | -0.003 | 0.026 | 0.002 |  |
|  | CMCC-CM |  |  | -0.008 | -0.008 | 0.016 | 0.000 |  |
|  | GFDL-CM3 |  |  | -0.005 | -0.007 | 0.027 | 0.005 |  |
|  | inmcm4 |  |  | -0.015 | -0.004 | 0.016 | -0.001 |  |
|  | IPSL-CM5B-LR |  |  | -0.010 | 0.005 | 0.029 | 0.008 | 0.003 |


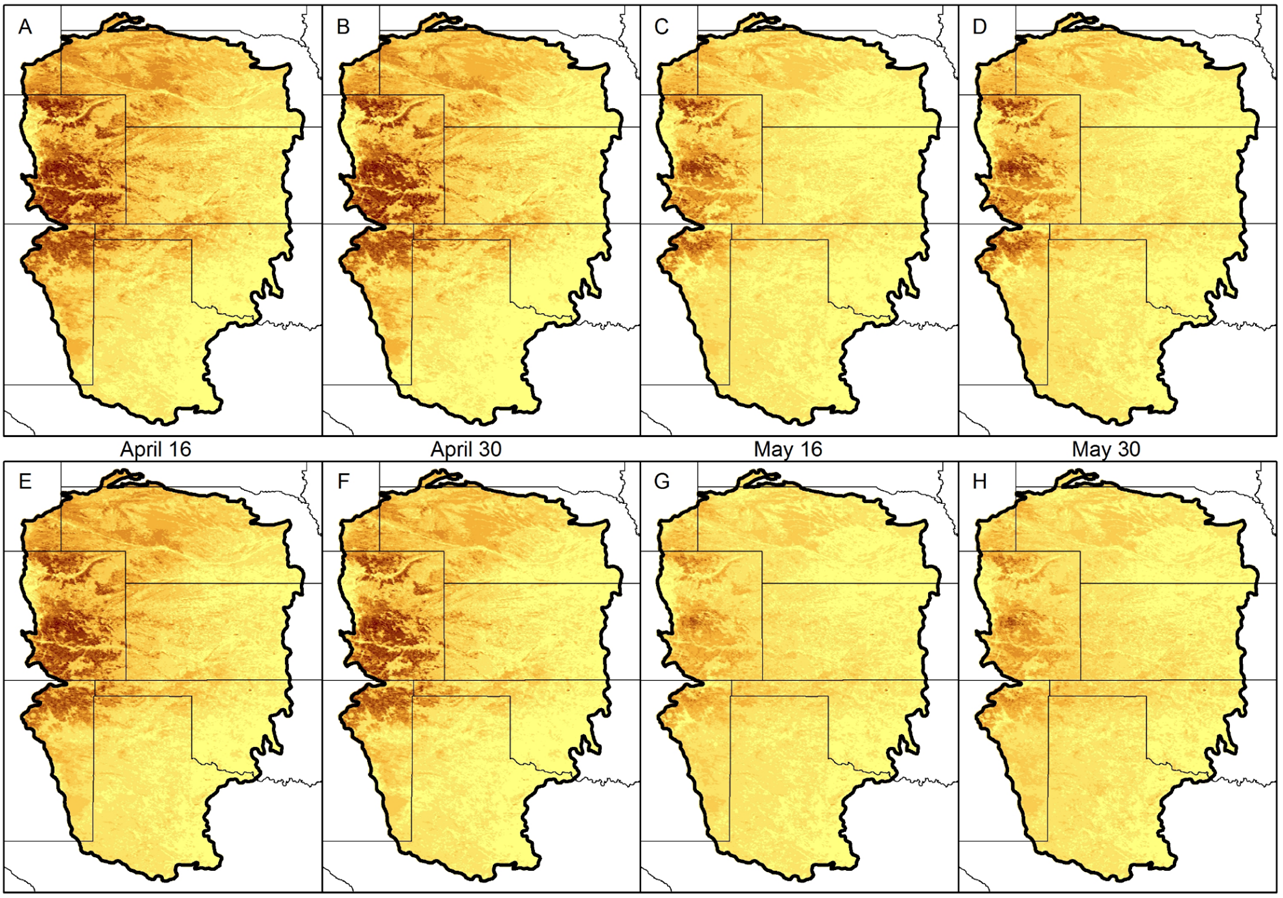


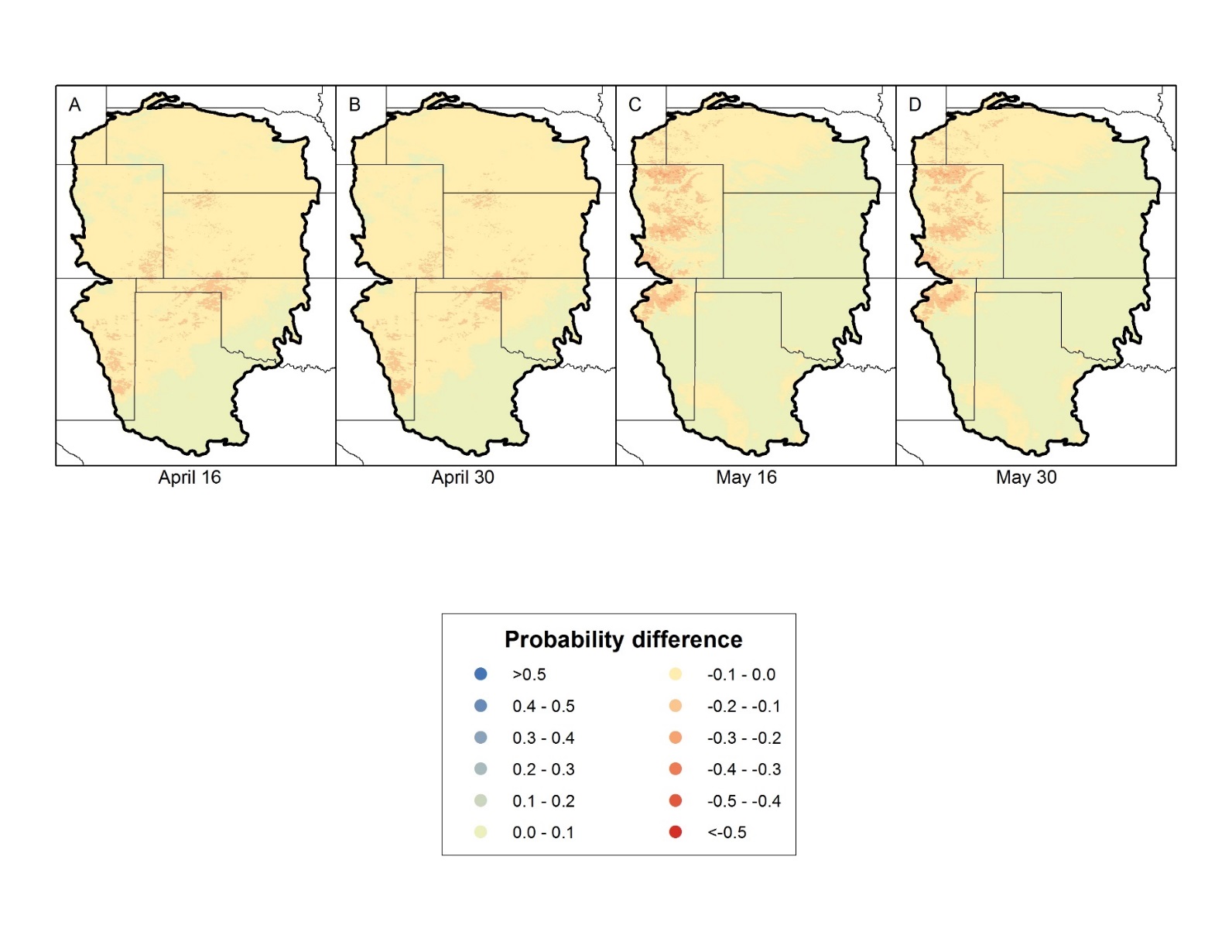

**
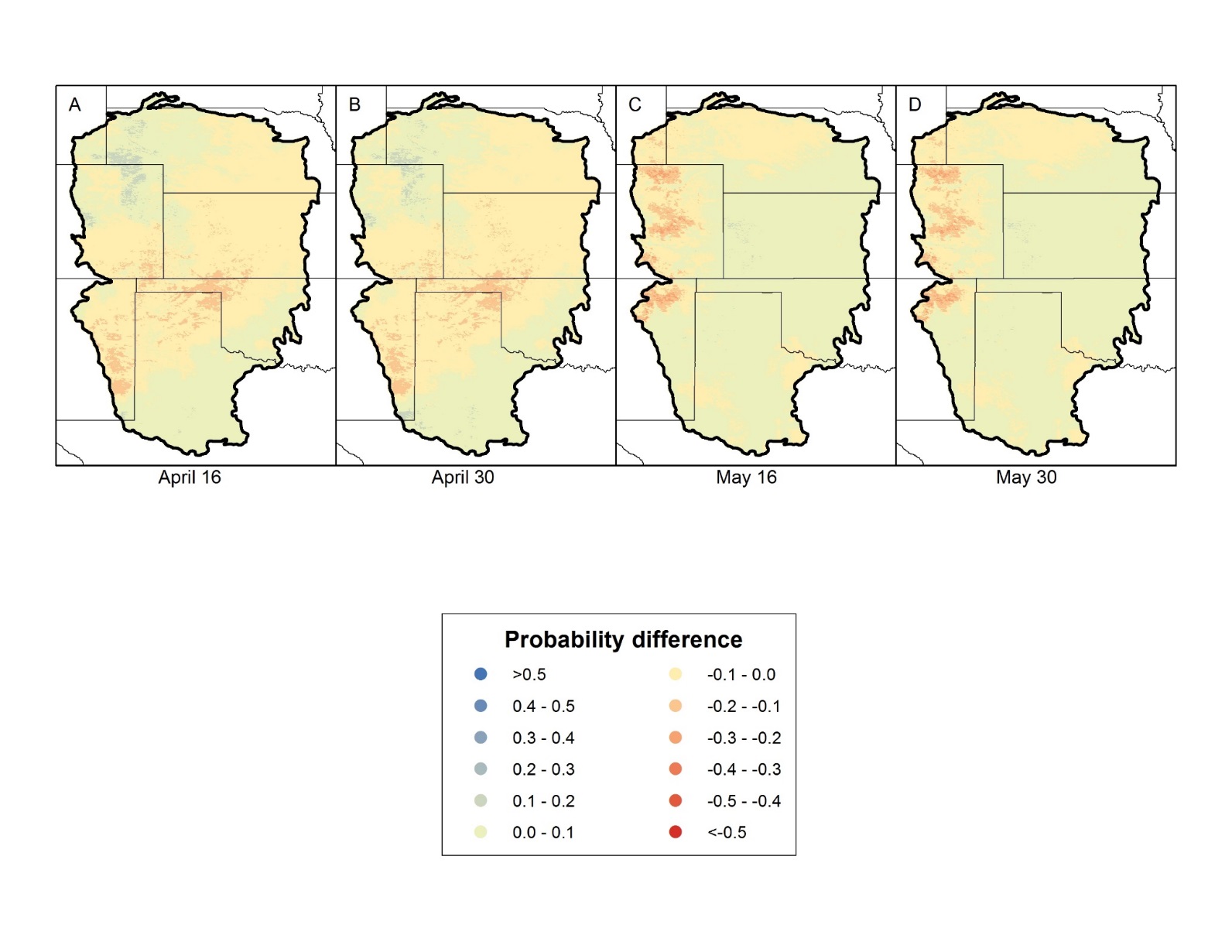
**

**Appendix 4a.** Probability of occurrence of Mountain Plover, 1981-2010 (top panel) and projected changes based on the ensemble (middle panel) and hot dry ACCESS1-0 GCM (bottom panel).


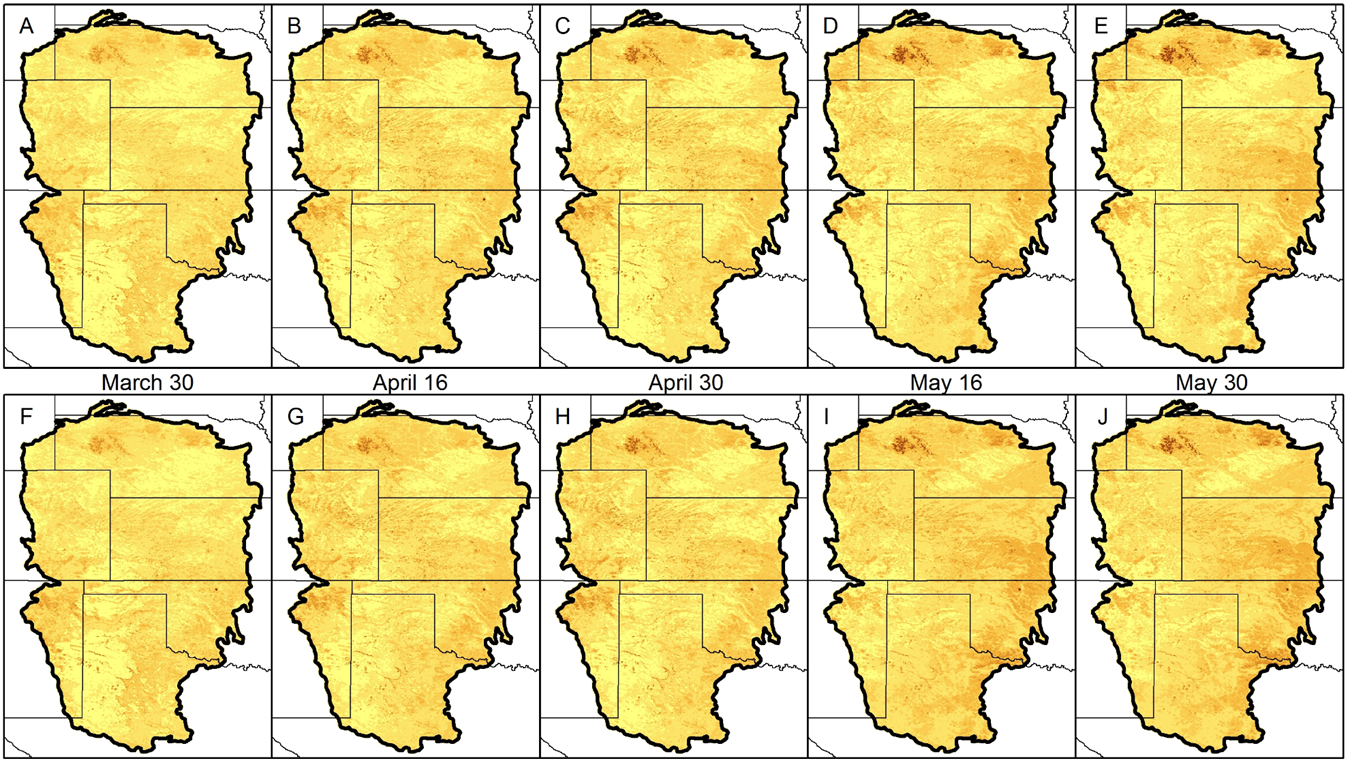


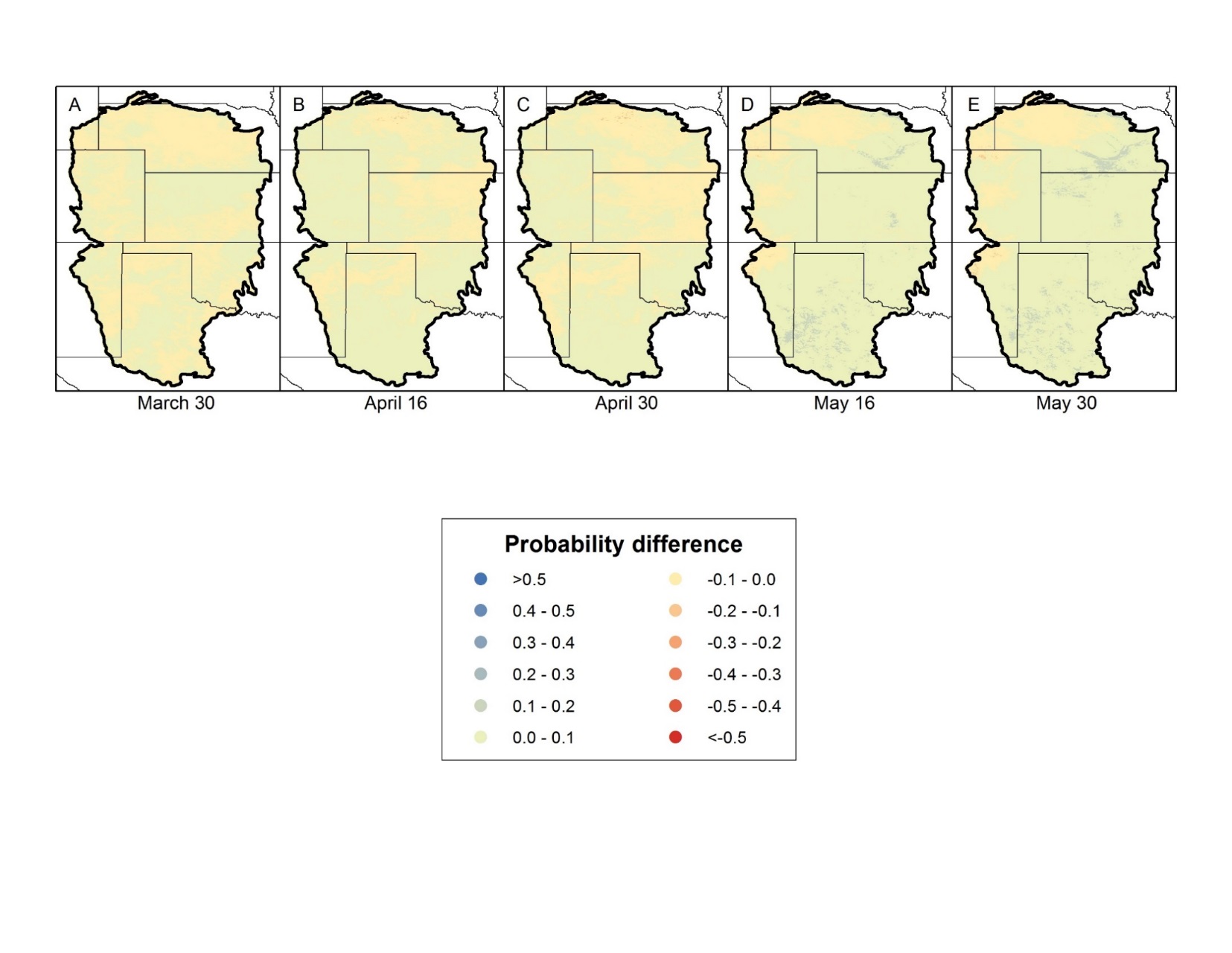

**
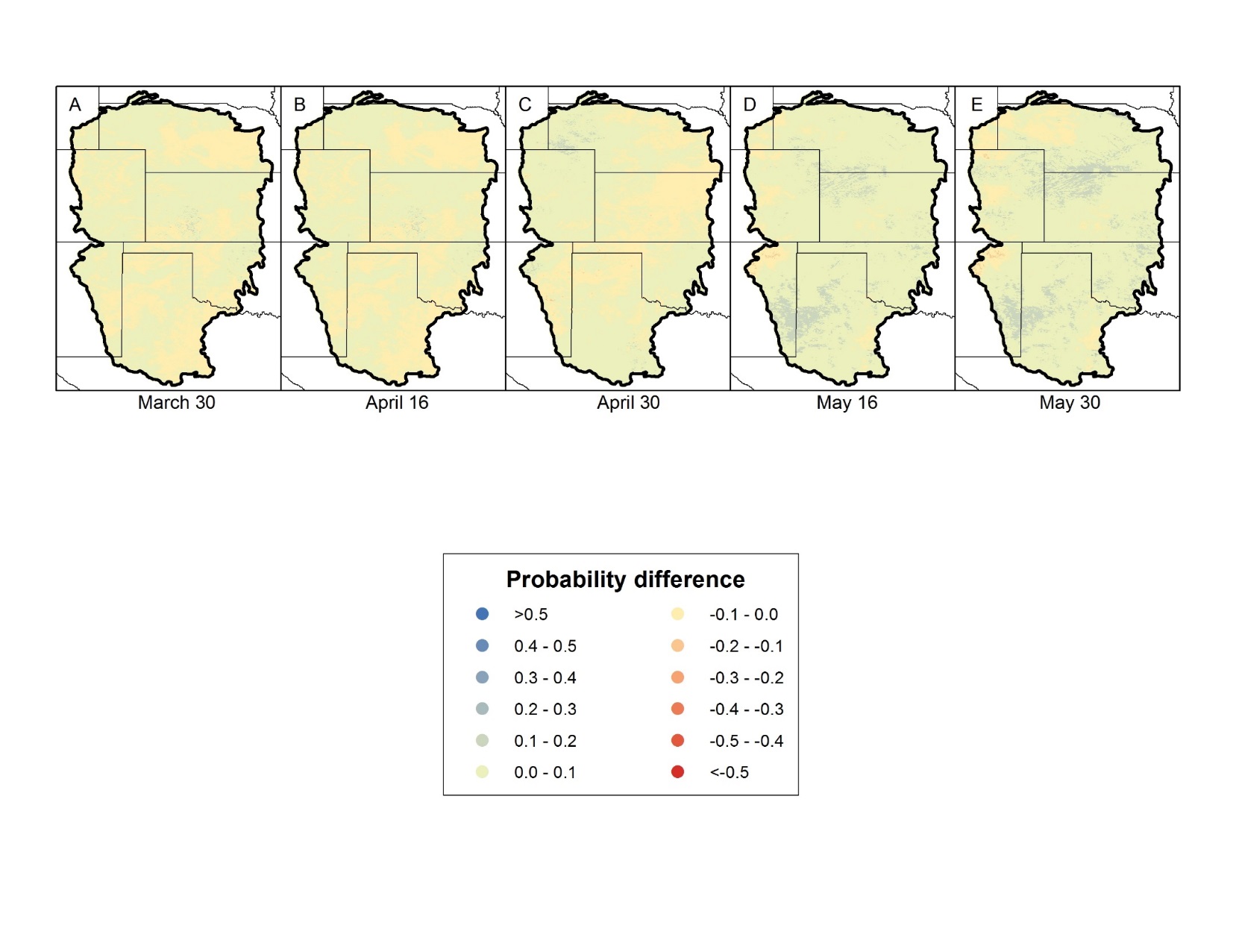
**

**Appendix 4b.** Probability of occurrence of American Avocet, 1981-2010 (top panel) and projected changes based on the ensemble (middle panel) and hot dry ACCESS1-0 GCM (bottom panel).


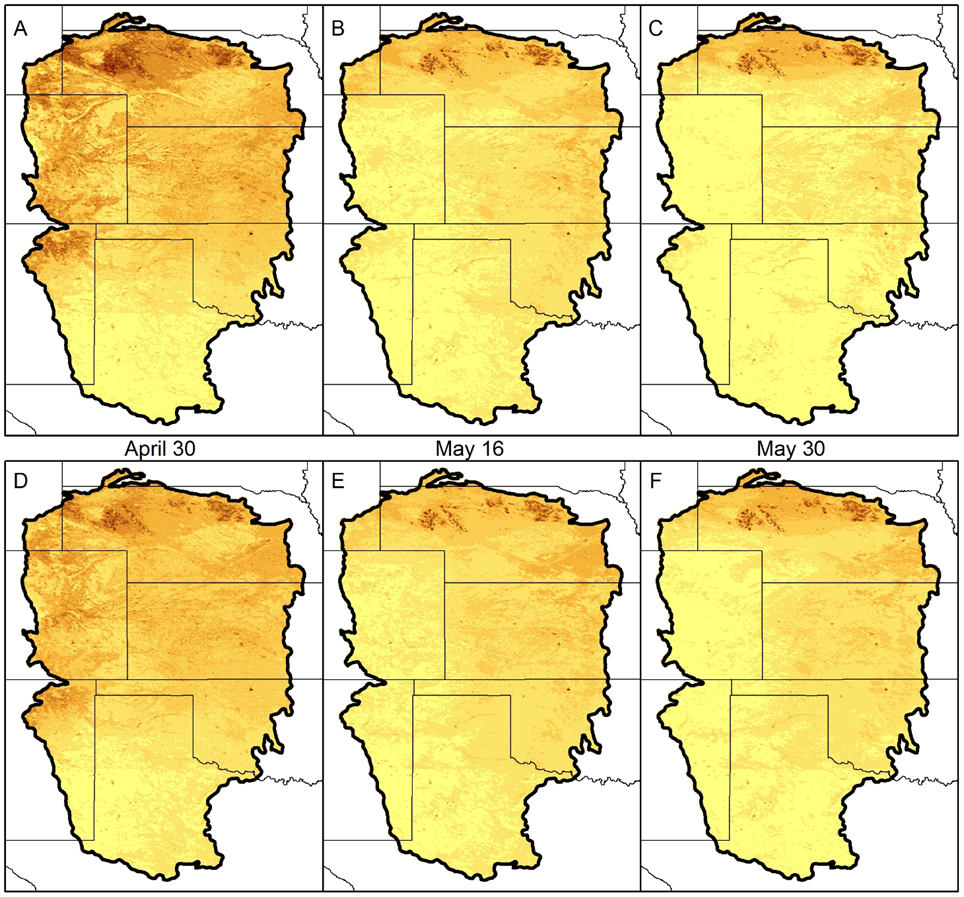


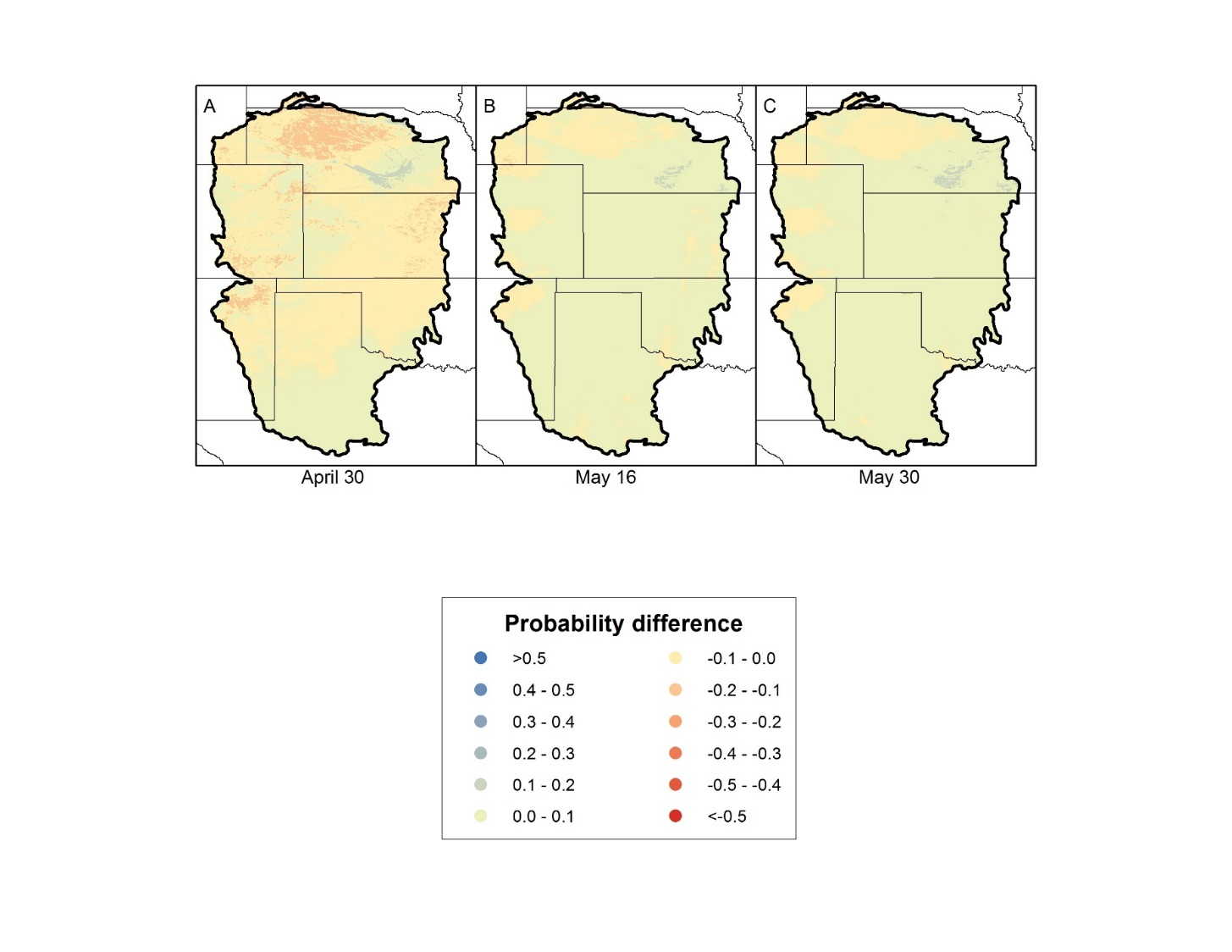

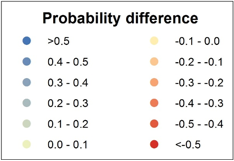

**
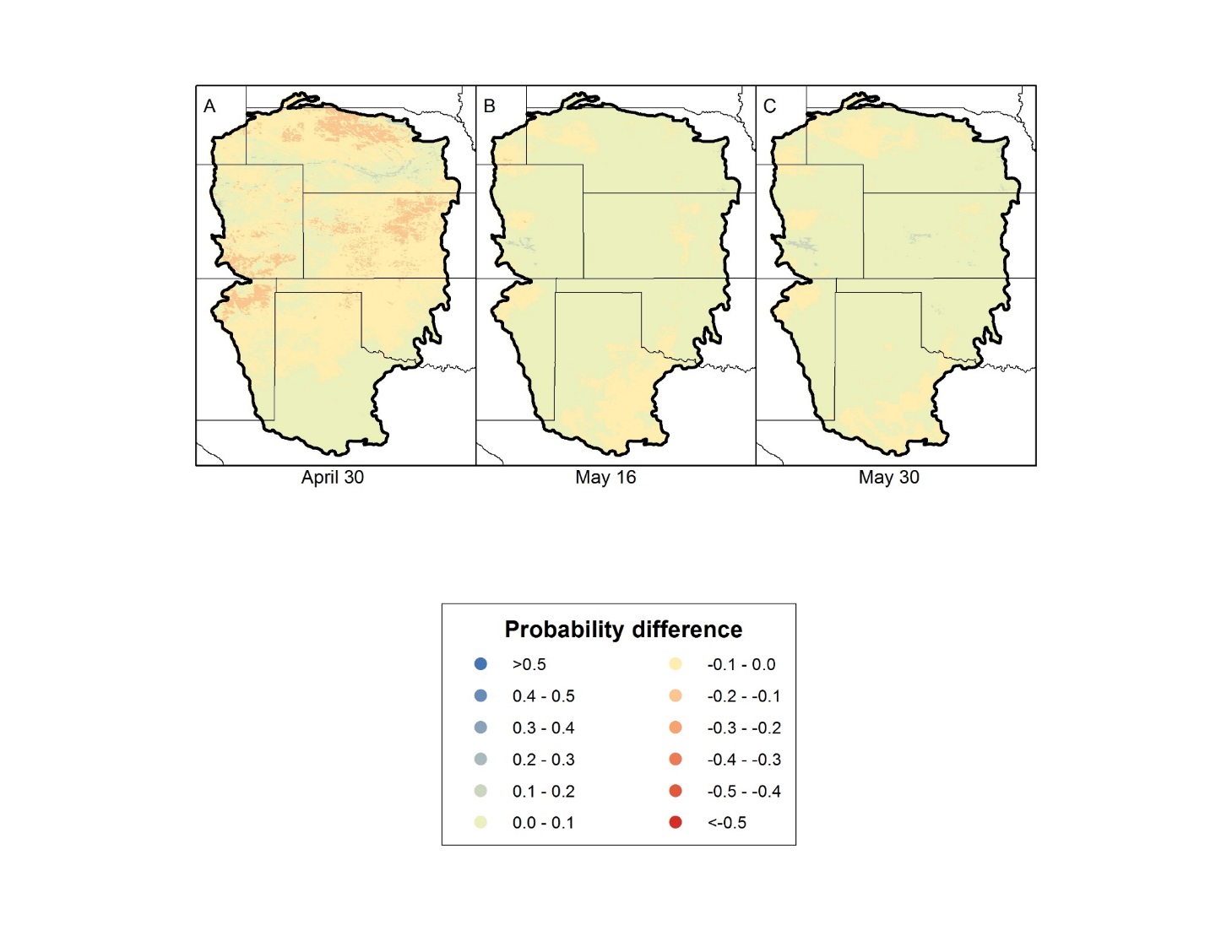
**

**Appendix 4c.** Probability of occurrence of Willet, 1981-2010 (top panel) and projected changes based on the ensemble (middle panel) and hot dry ACCESS1-0 GCM (bottom panel).


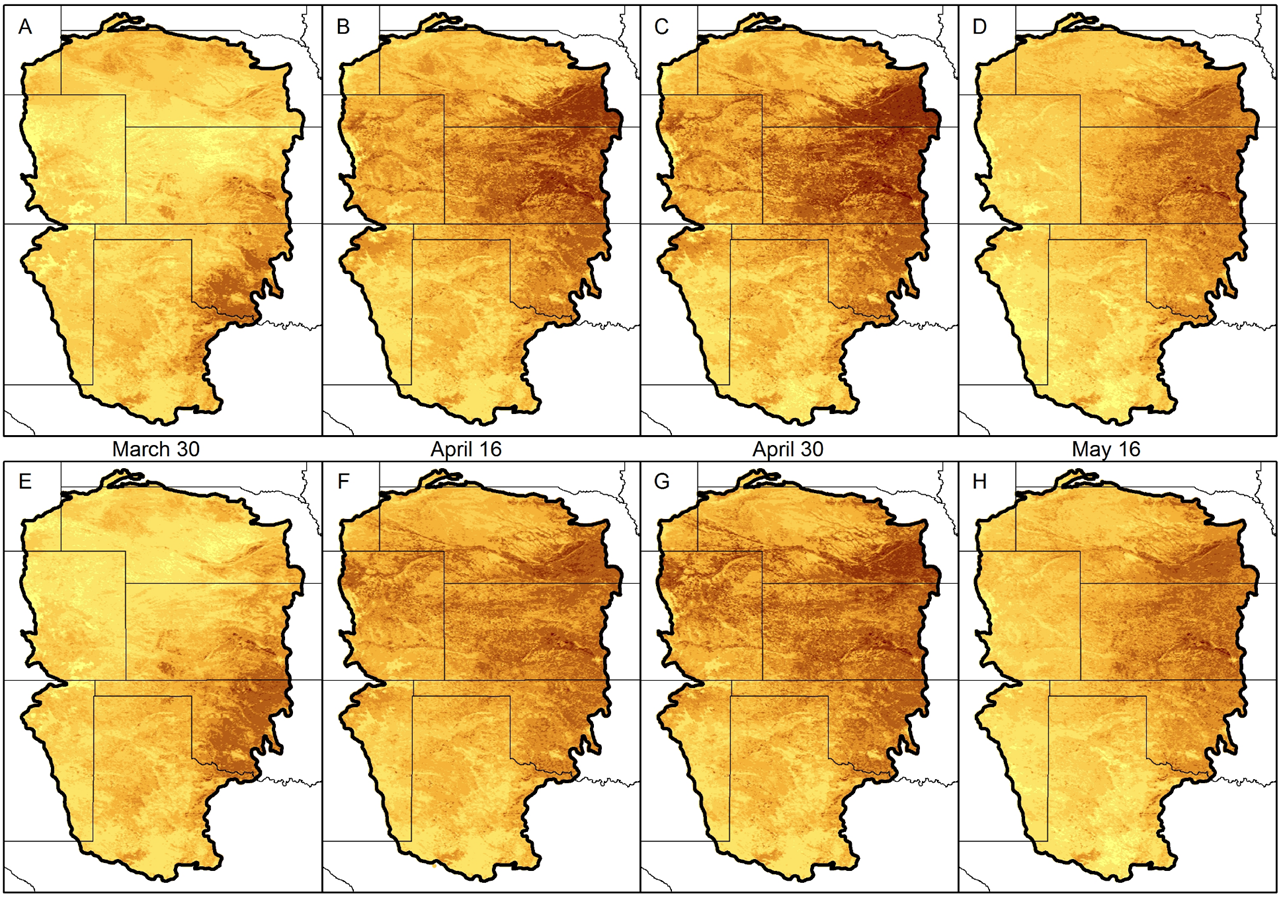


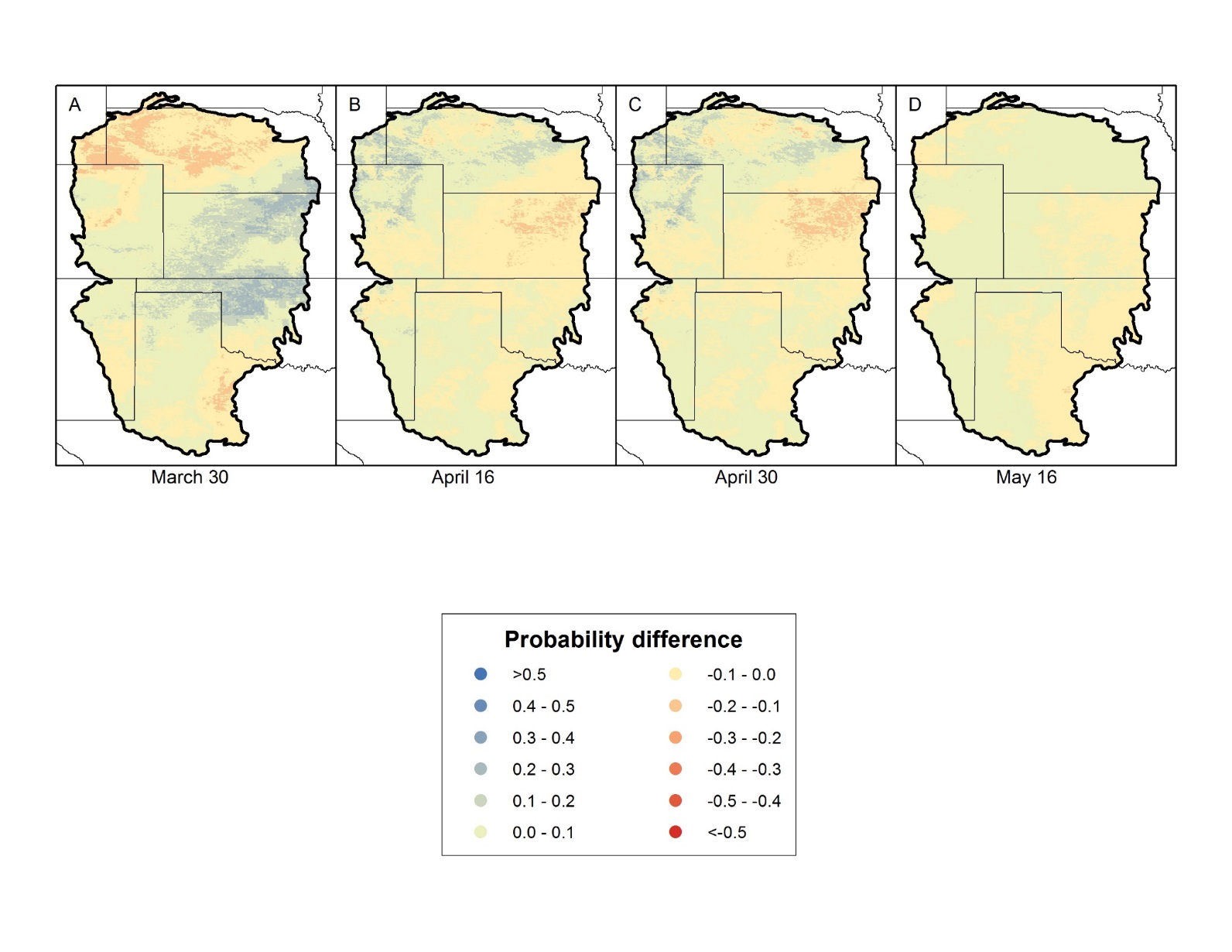

**
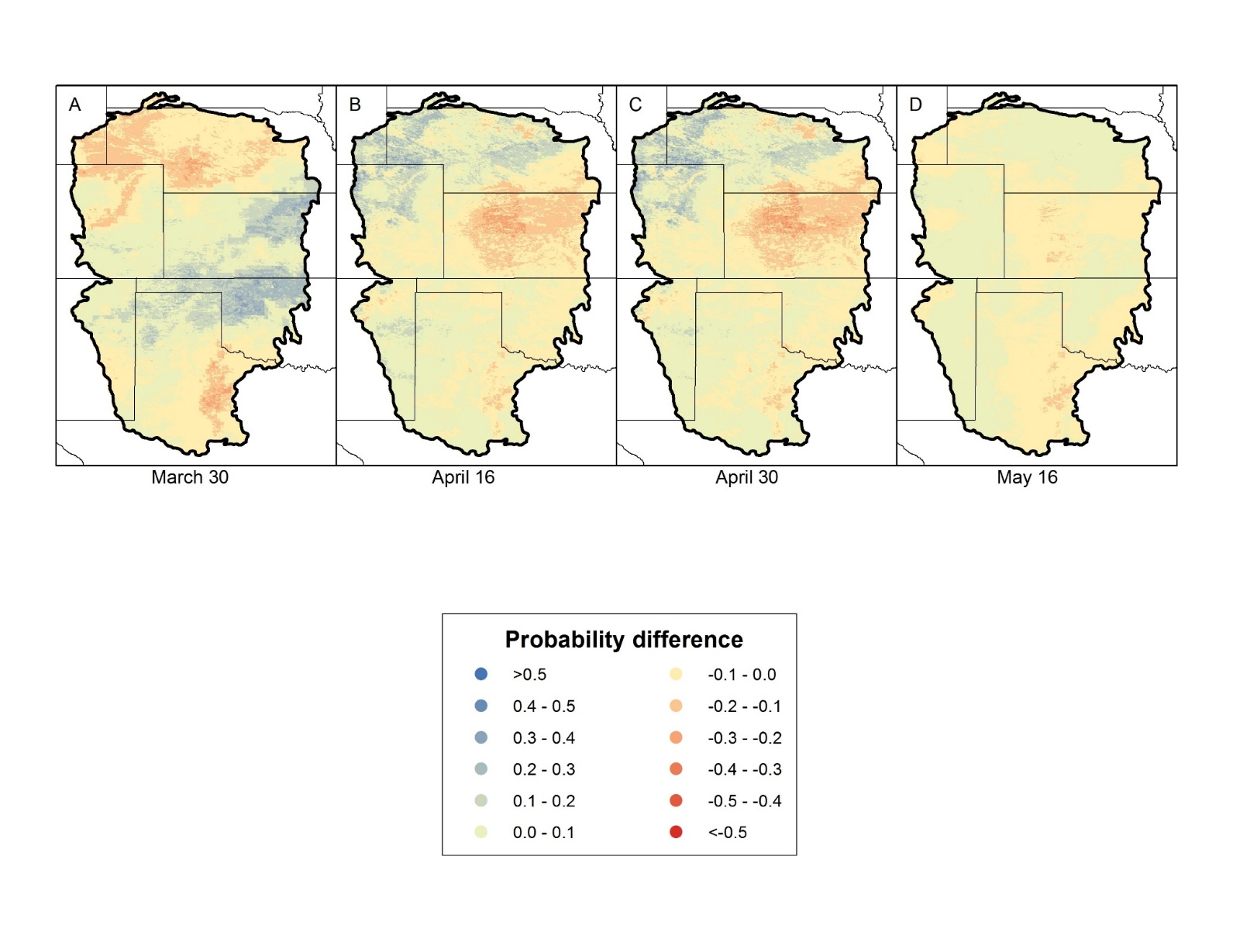
**

**Appendix 4d.** Probability of occurrence of Lesser Yellowlegs, 1981-2010 (top panel) and projected changes based on the ensemble (middle panel) and hot dry ACCESS1-0 GCM (bottom panel).


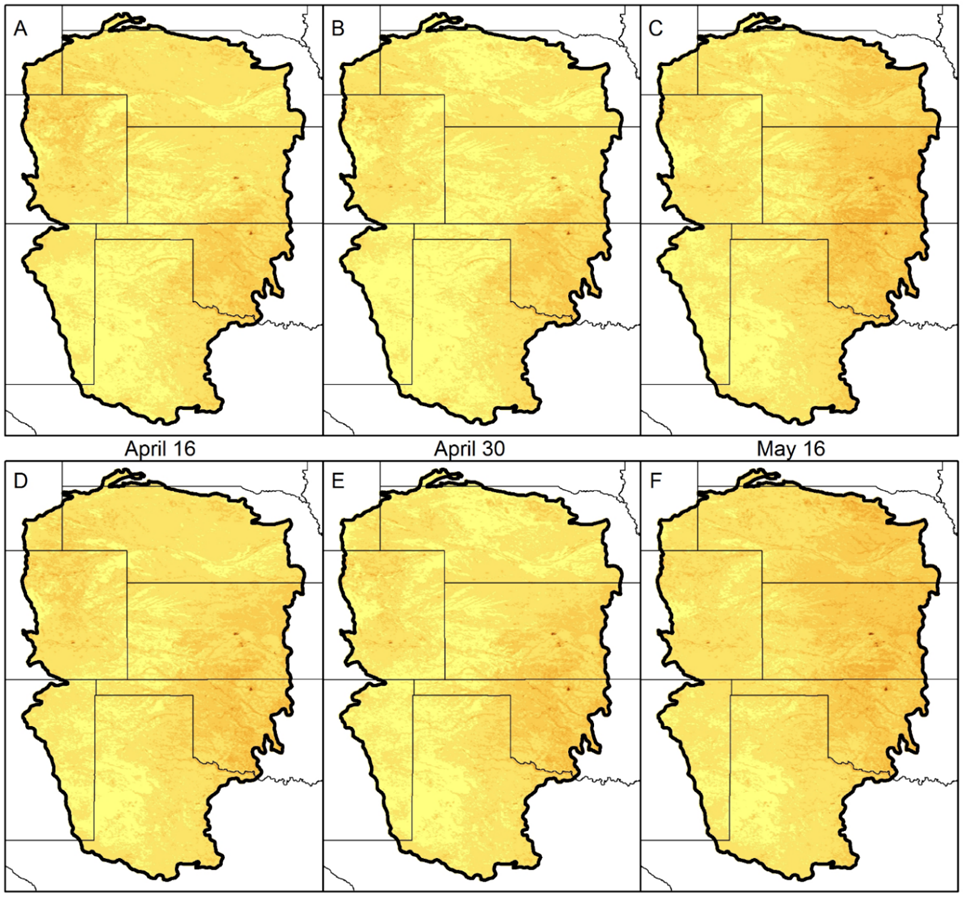


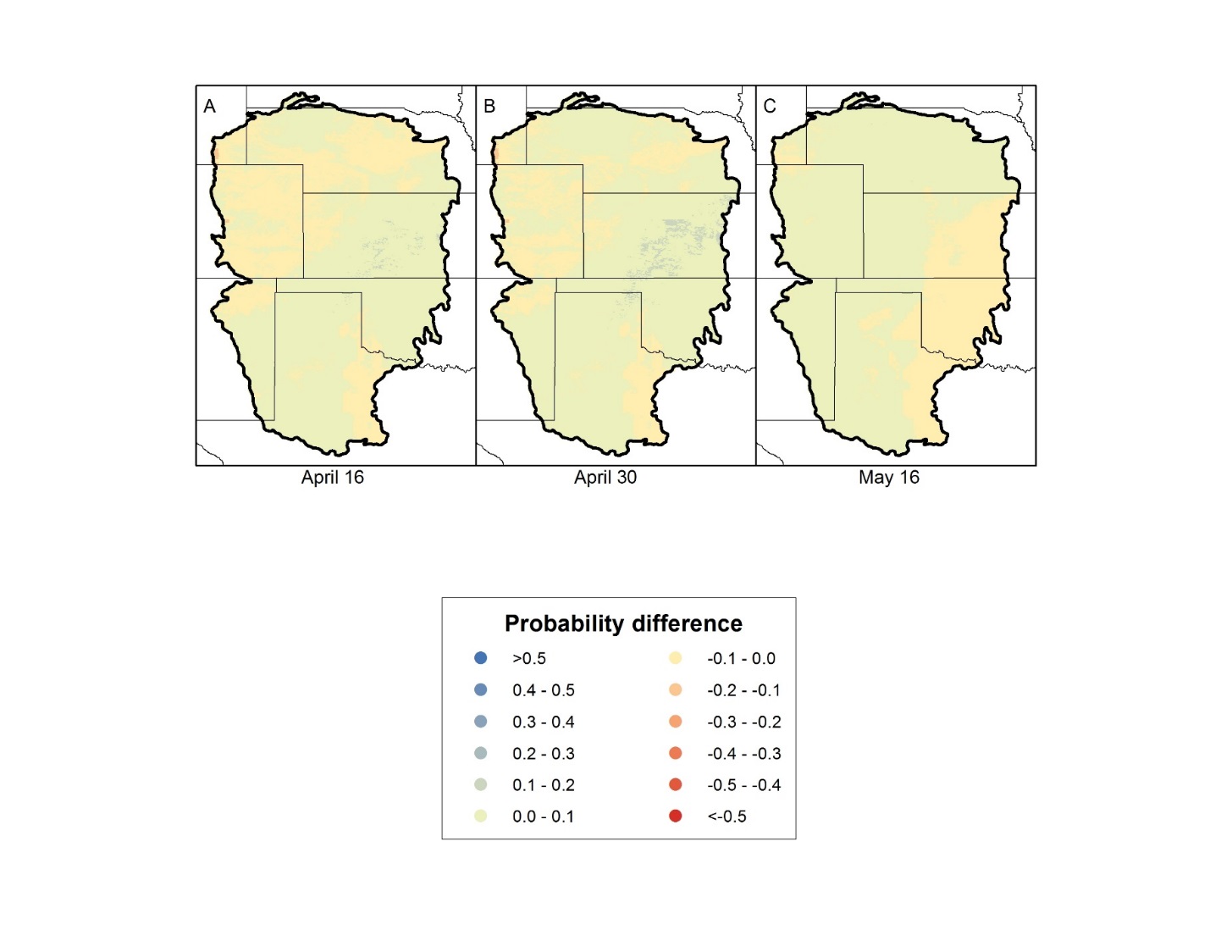

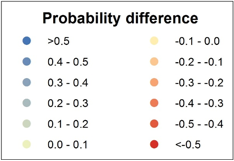

**
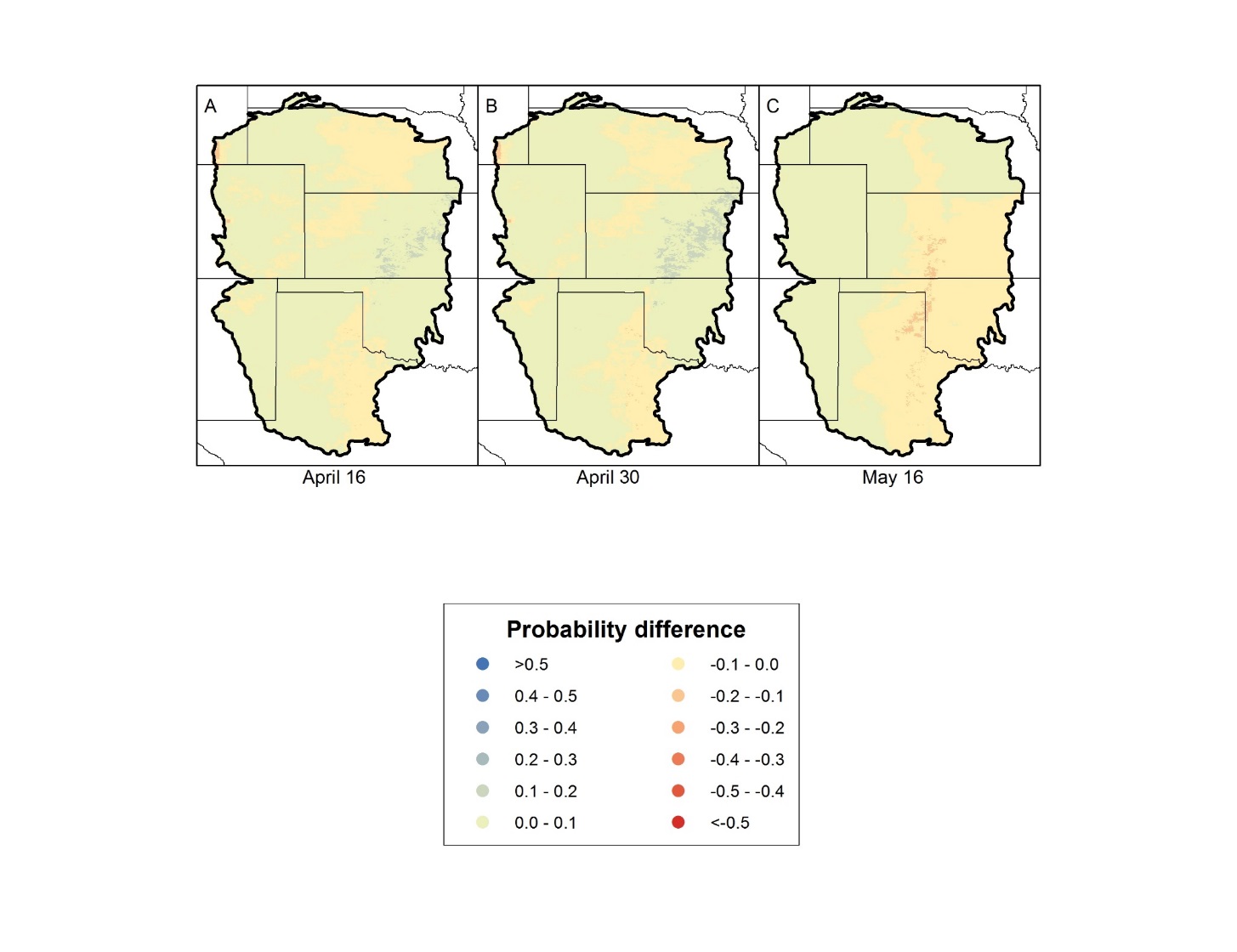
**

**Appendix 4e.** Probability of occurrence of Whimbrel, 1981-2010 (top panel) and projected changes based on the ensemble (middle panel) and hot dry ACCESS1-0 GCM (bottom panel).


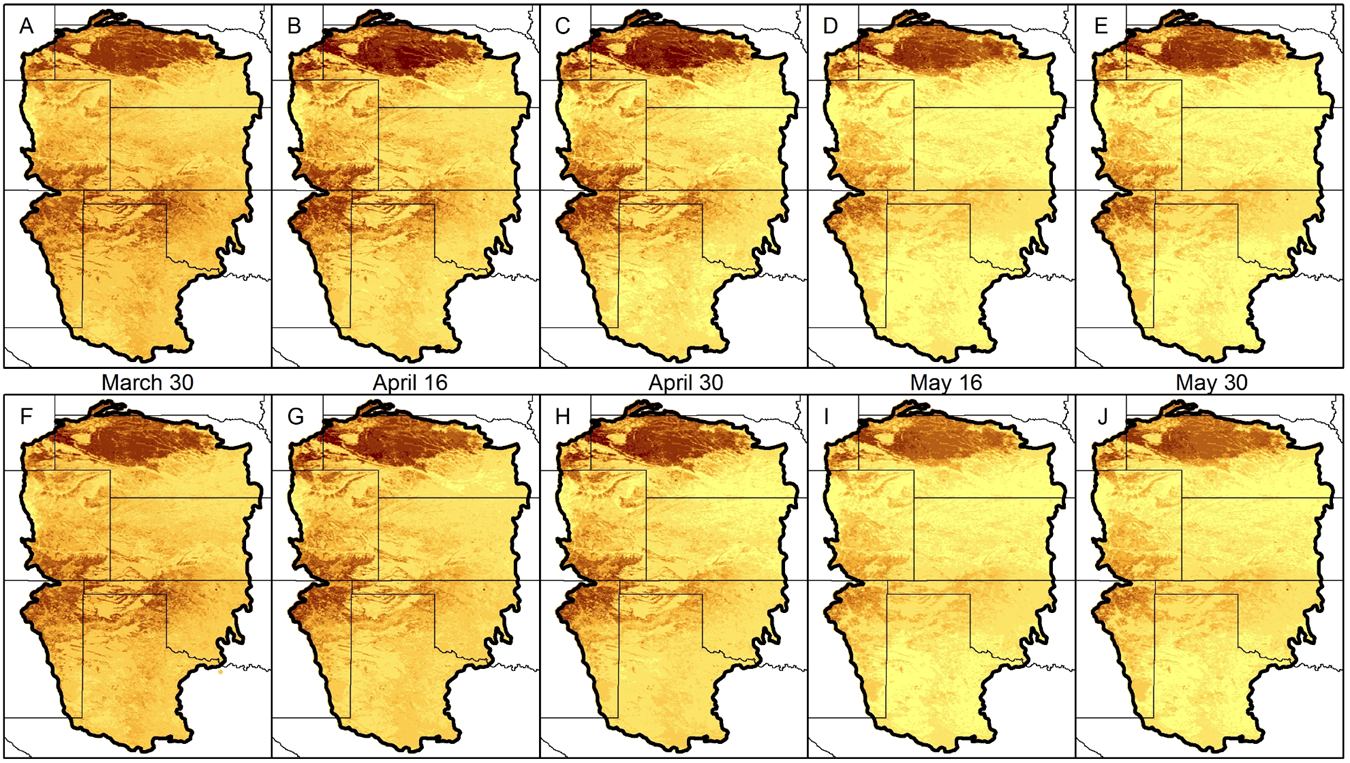


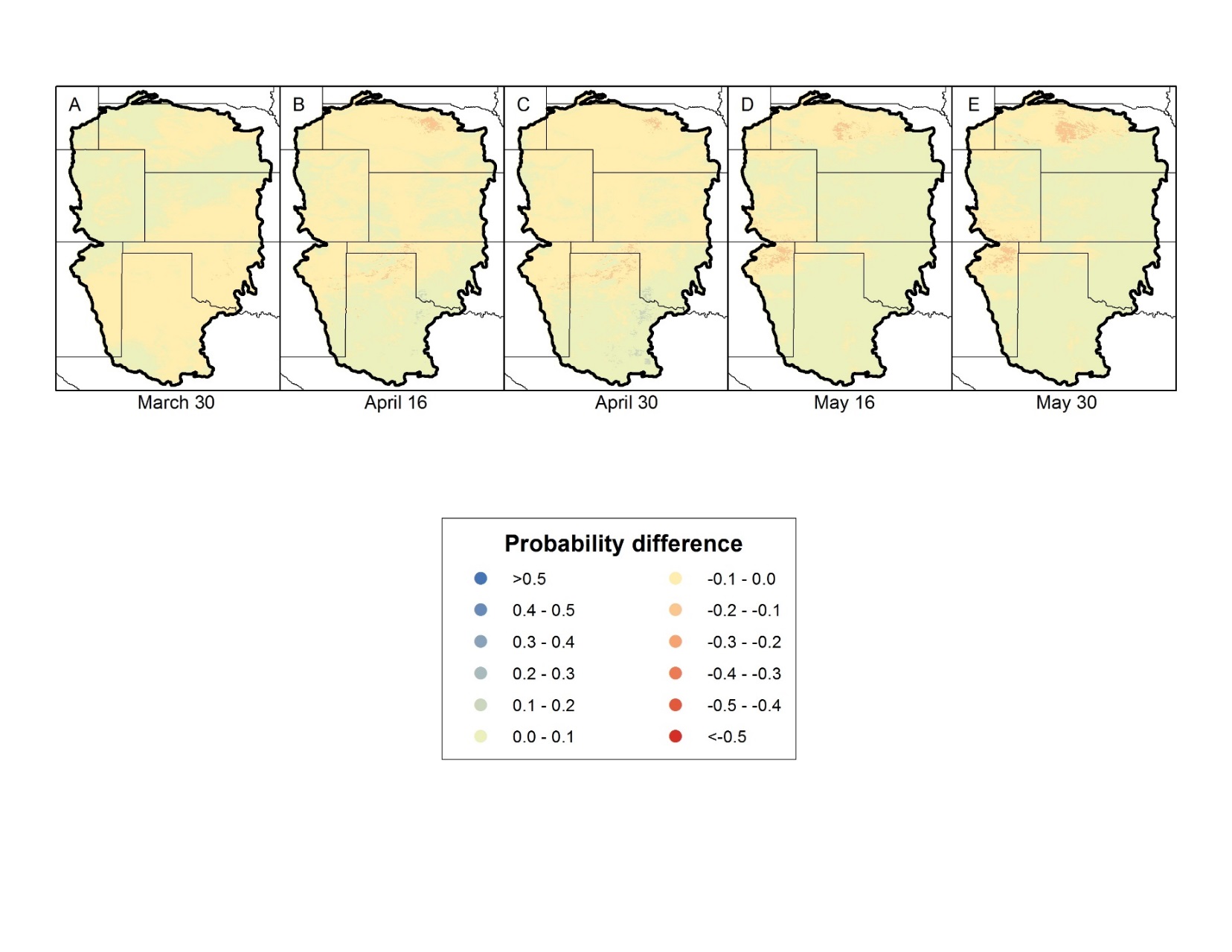

**
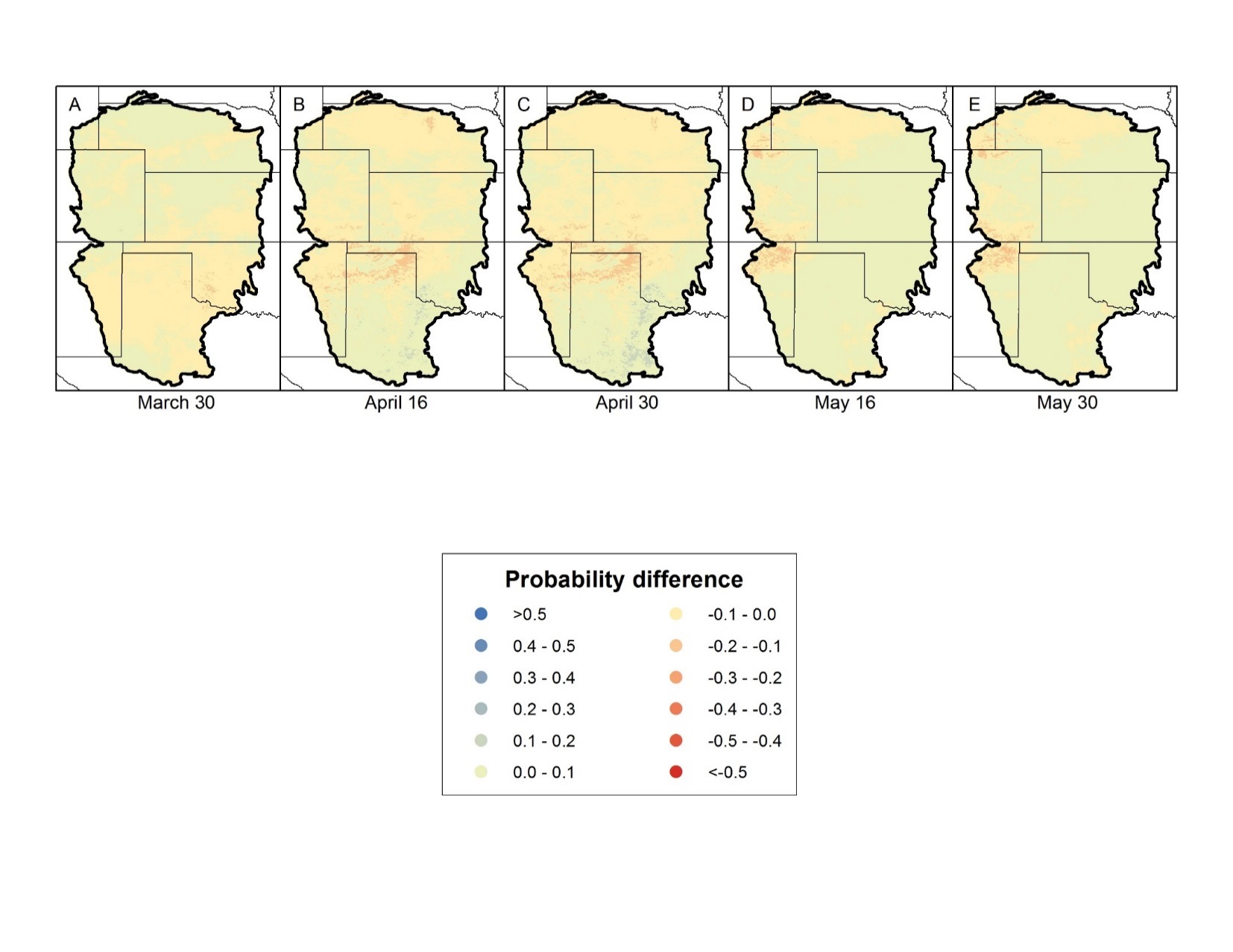
**

**Appendix 4f.** Probability of occurrence of Long-billed Curlew, 1981-2010 (top panel) and projected changes based on the ensemble (middle panel) and hot dry ACCESS1-0 GCM (bottom panel).

**
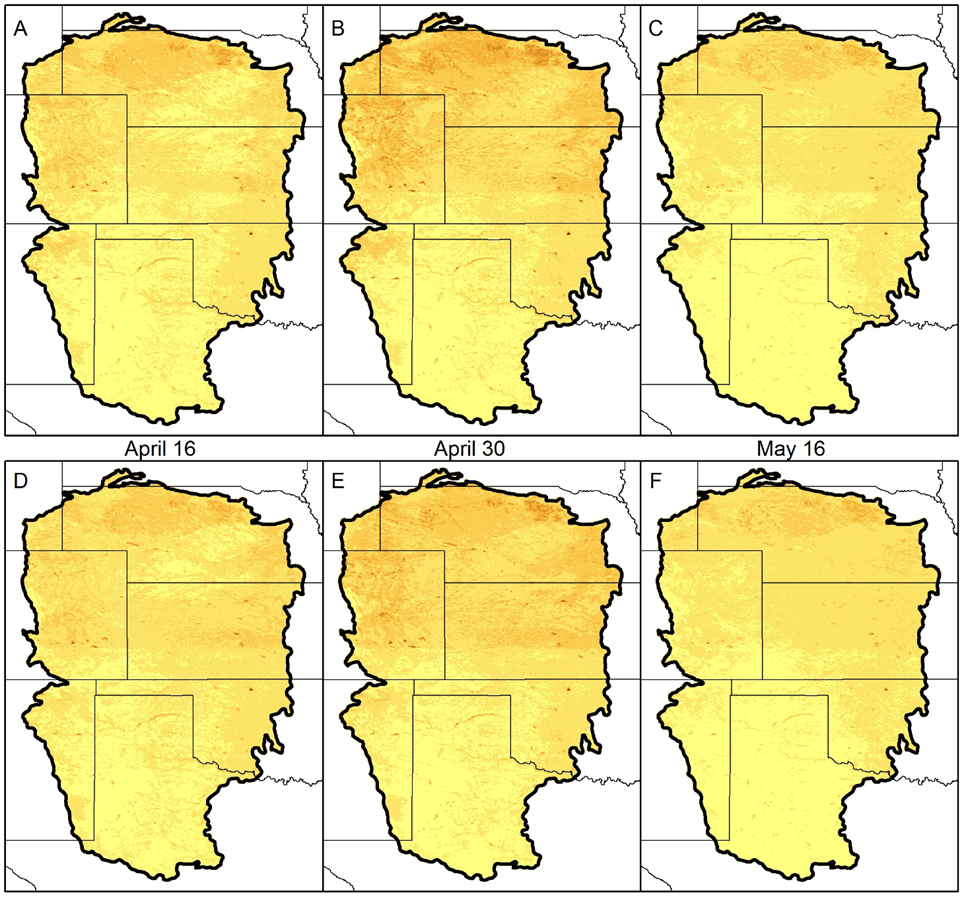
**


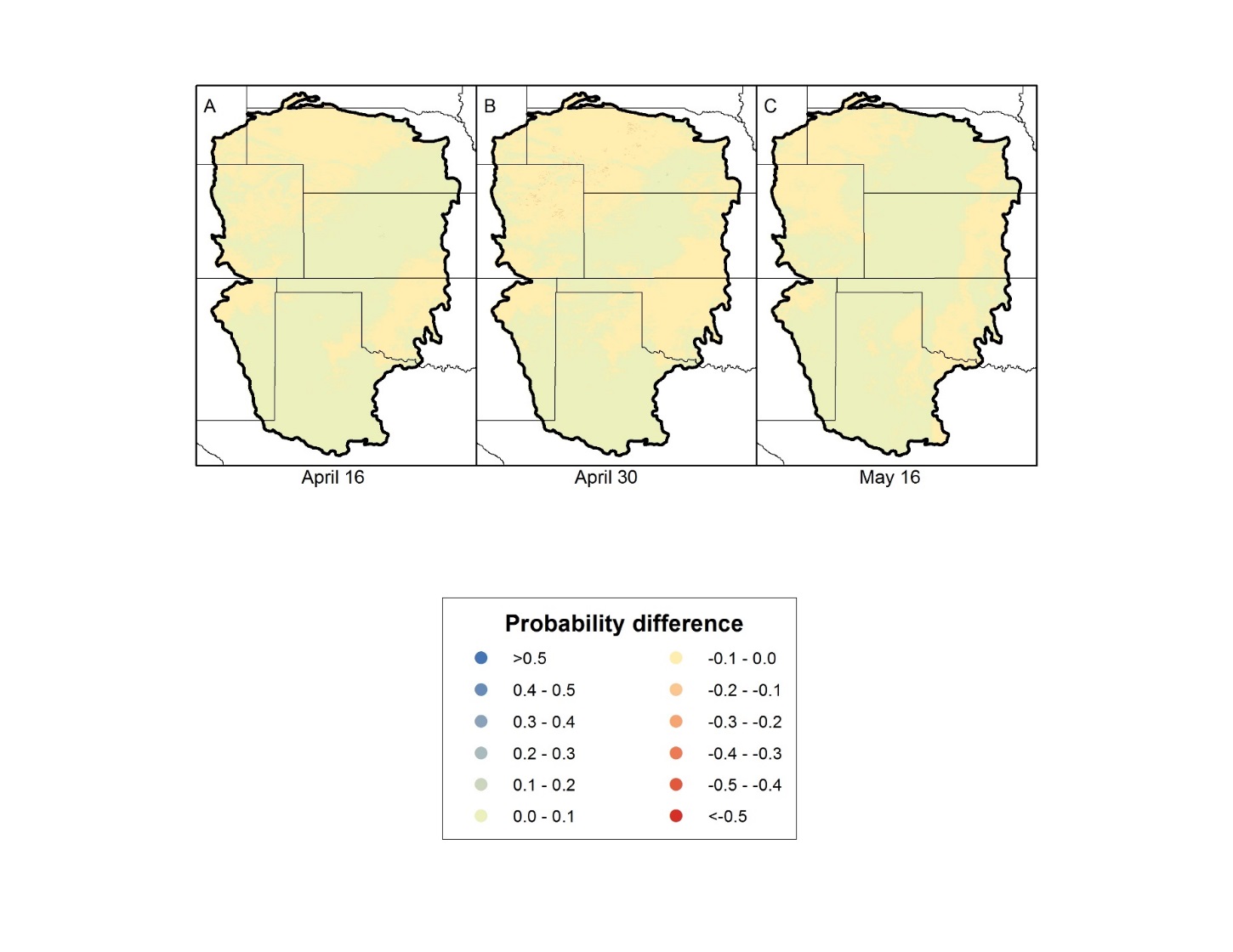

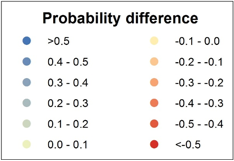

**
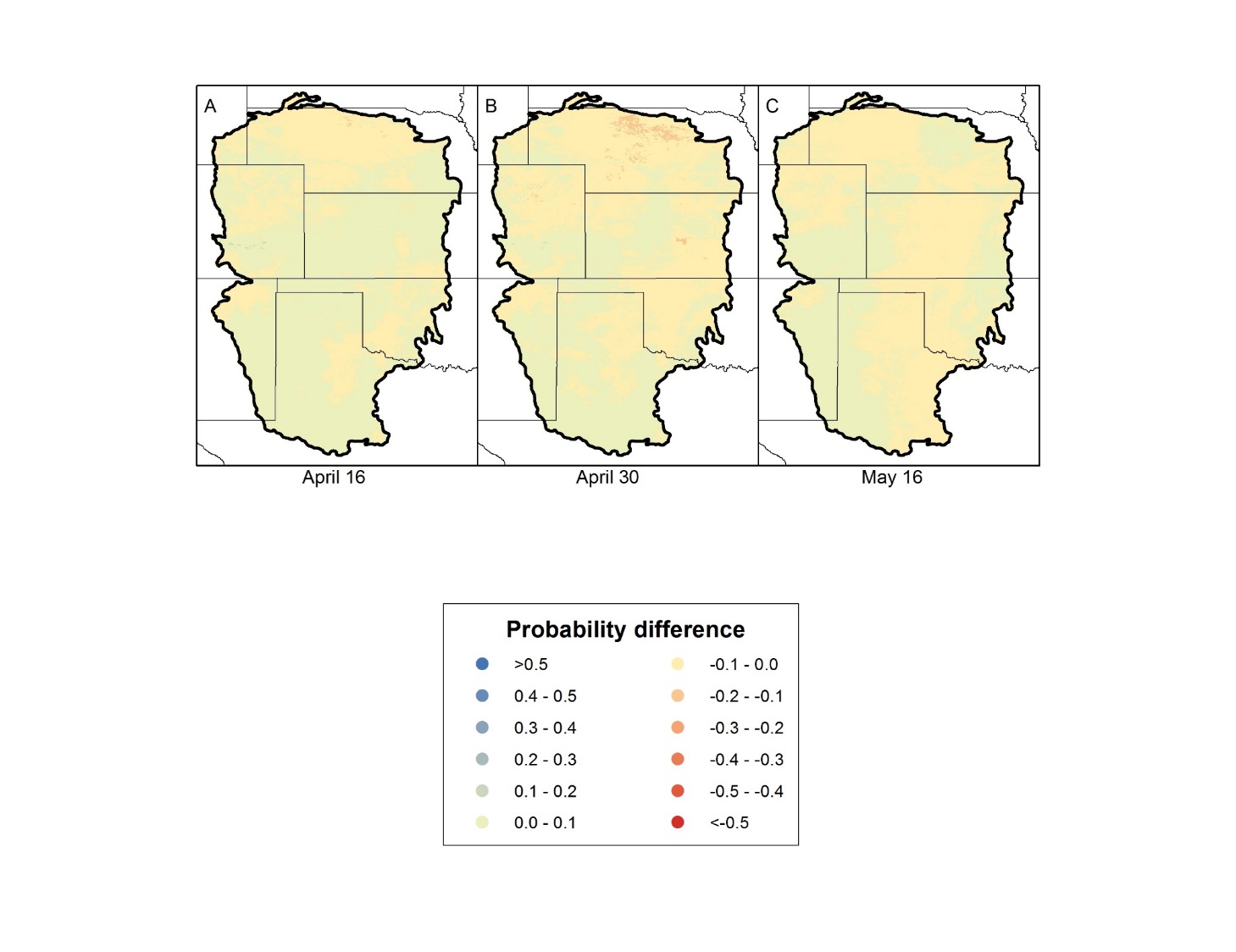
**

**Appendix 4g.** Probability of occurrence of Marbled Godwit, 1981-2010 (top panel) and projected changes based on the ensemble (middle panel) and hot dry ACCESS1-0 GCM (bottom panel).


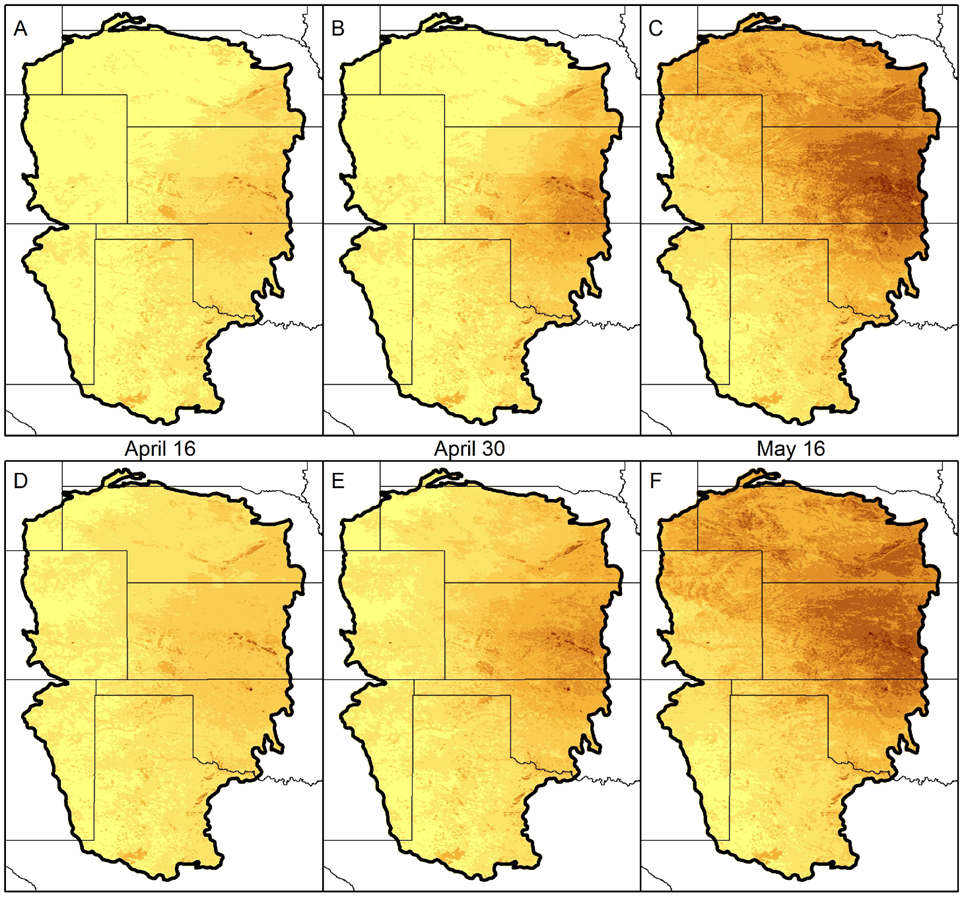


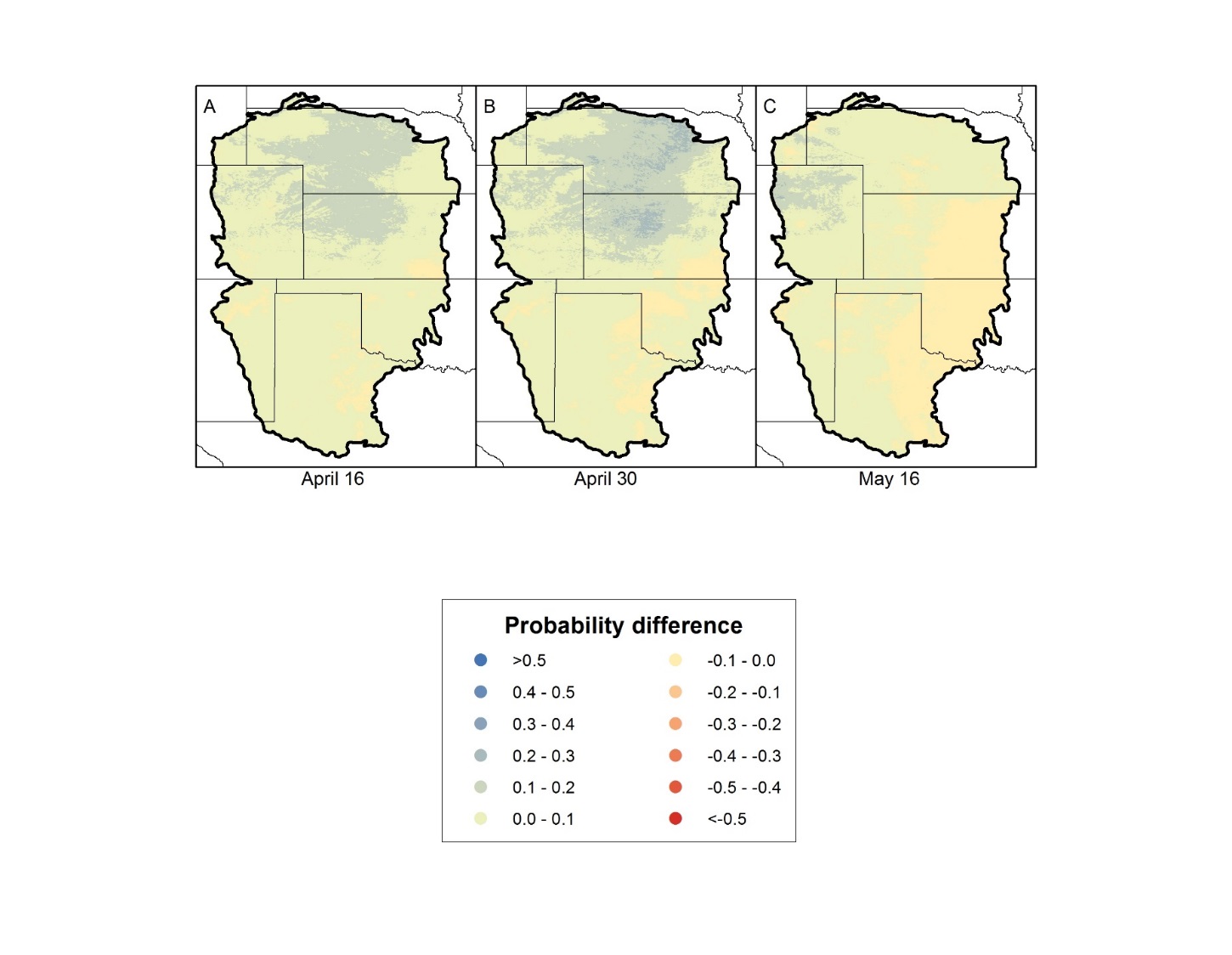

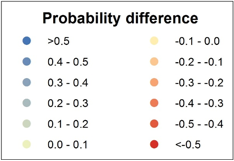

**
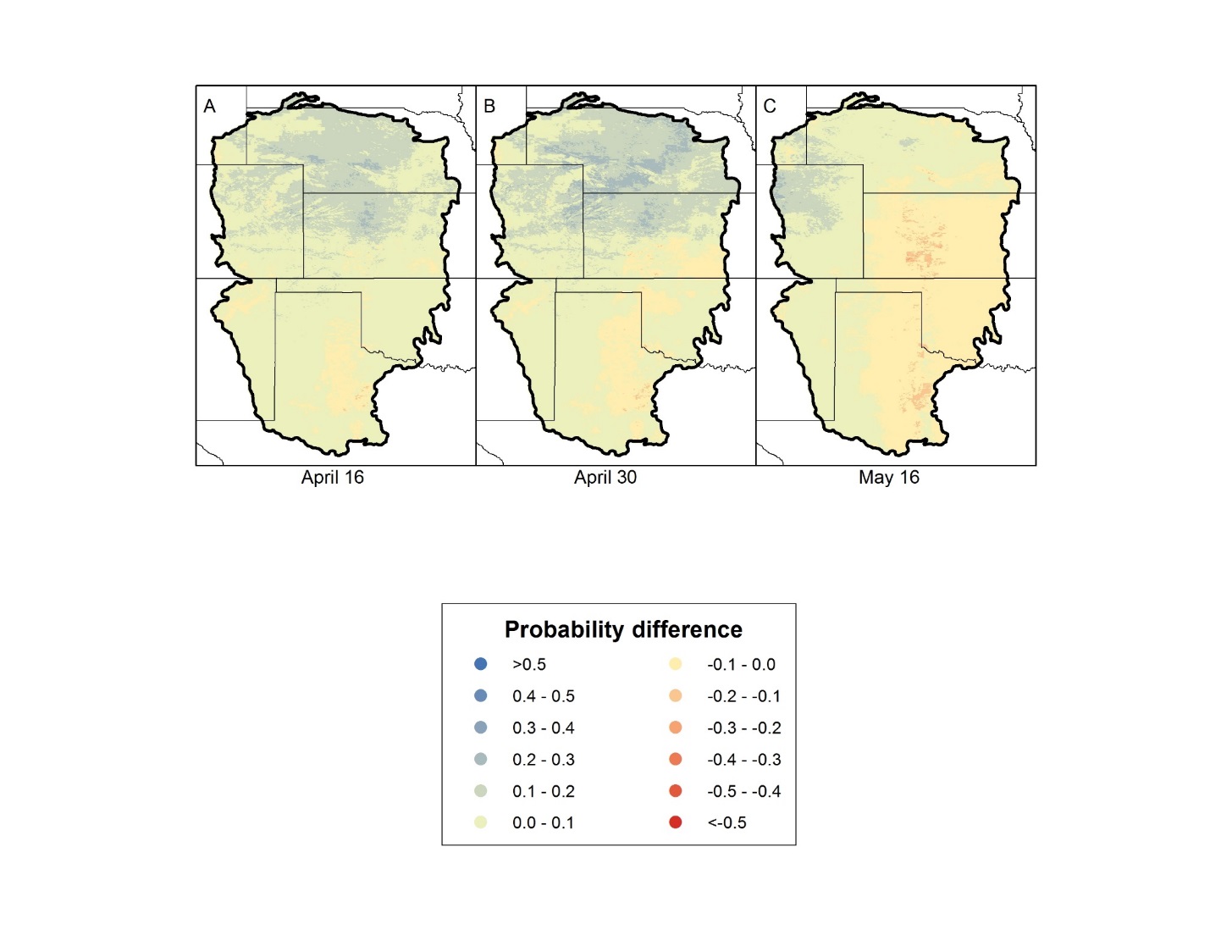
**

**Appendix 4h.** Probability of occurrence of Stilt Sandpiper, 1981-2010 (top panel) and projected changes based on the ensemble (middle panel) and hot dry ACCESS1-0 GCM (bottom panel).


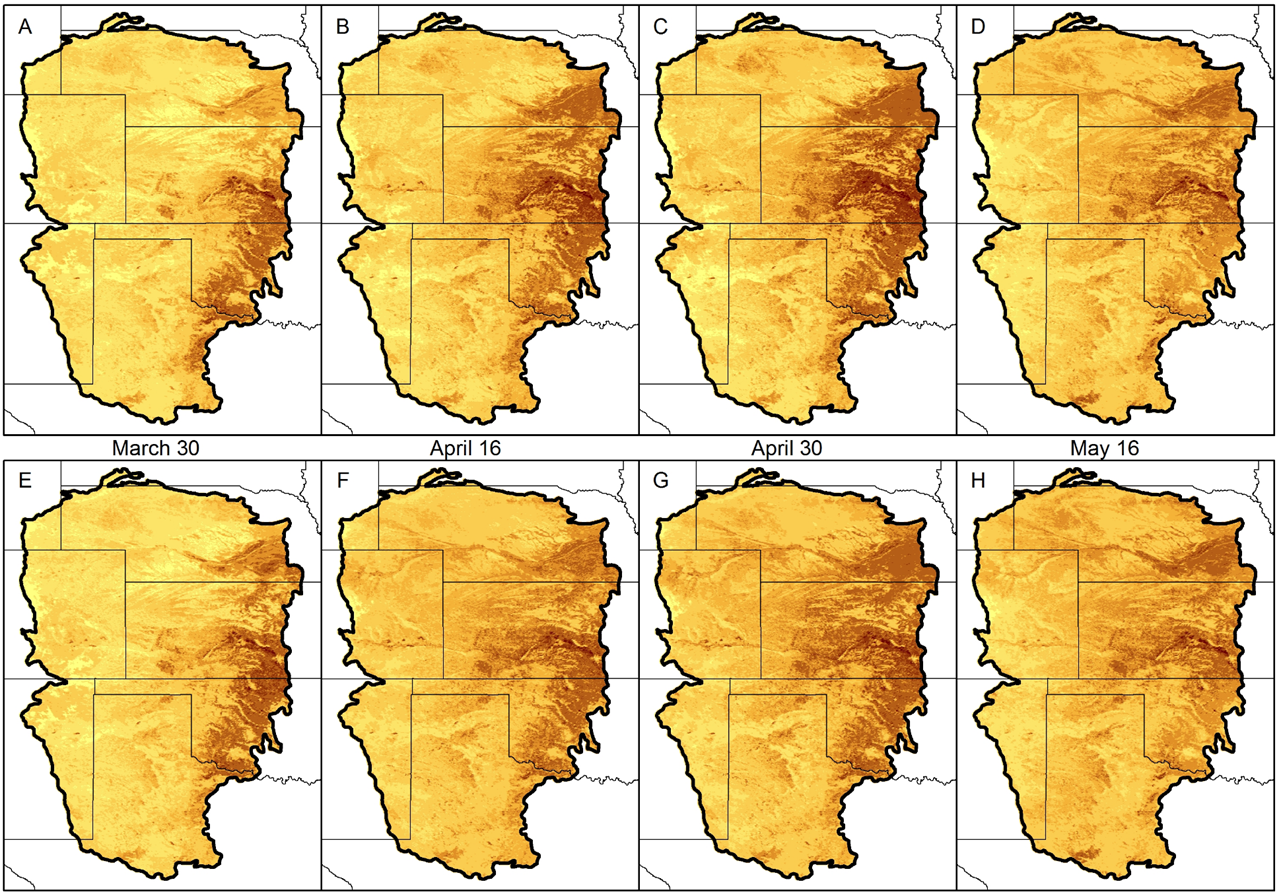


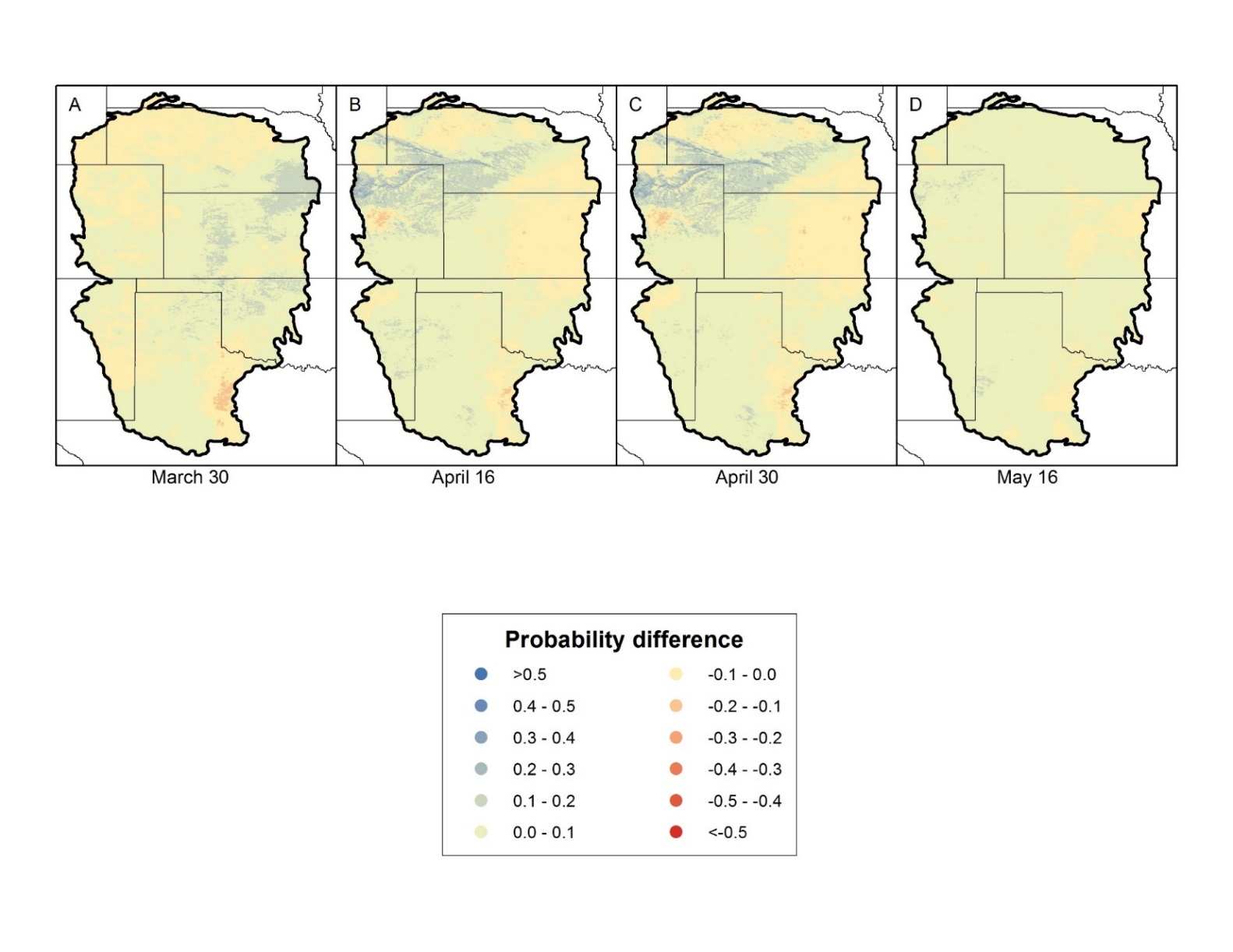

**
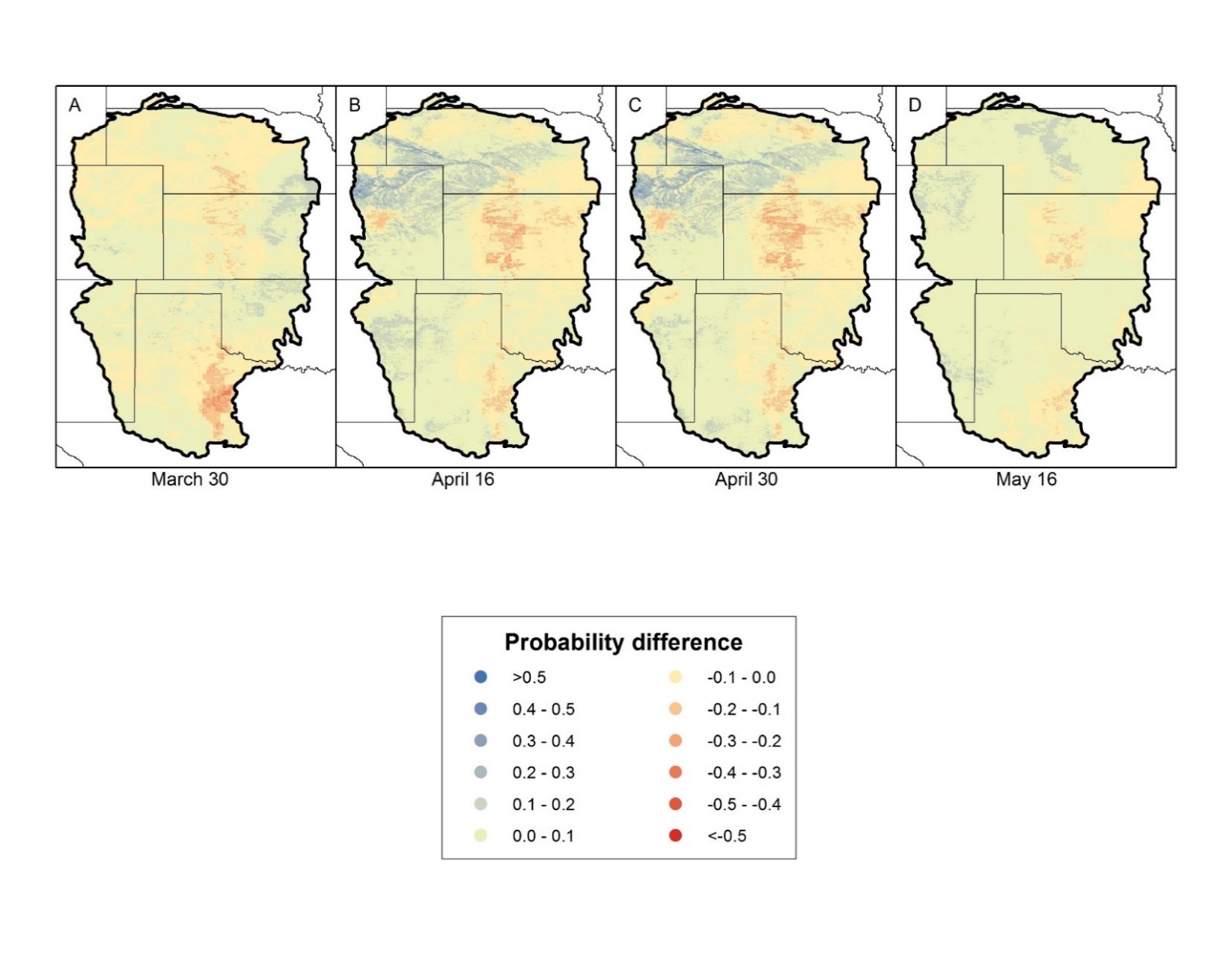
**

**Appendix 4i.** Probability of occurrence of Baird’s Sandpiper, 1981-2010 (top panel) and projected changes based on the ensemble (middle panel) and hot dry ACCESS1-0 GCM (bottom panel).


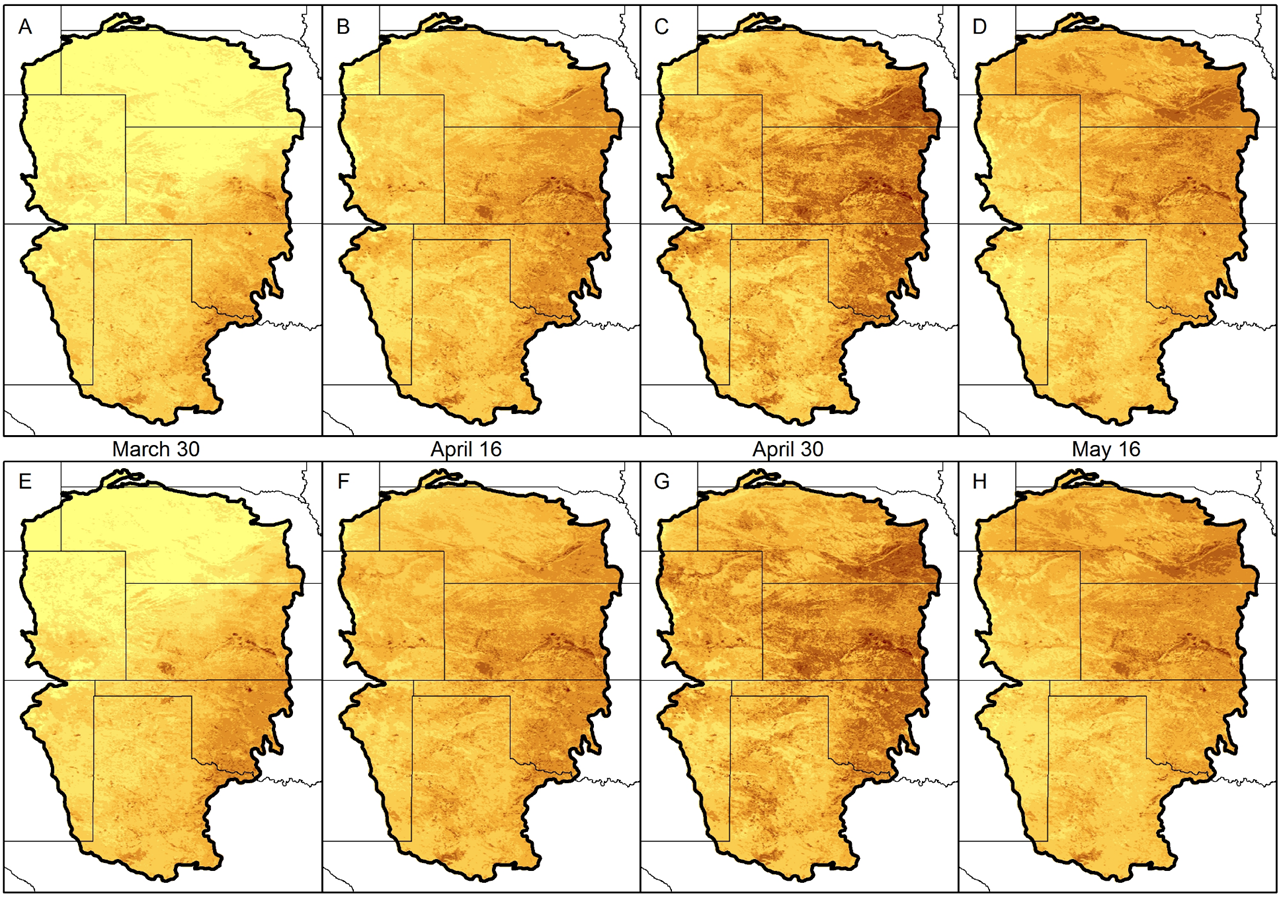


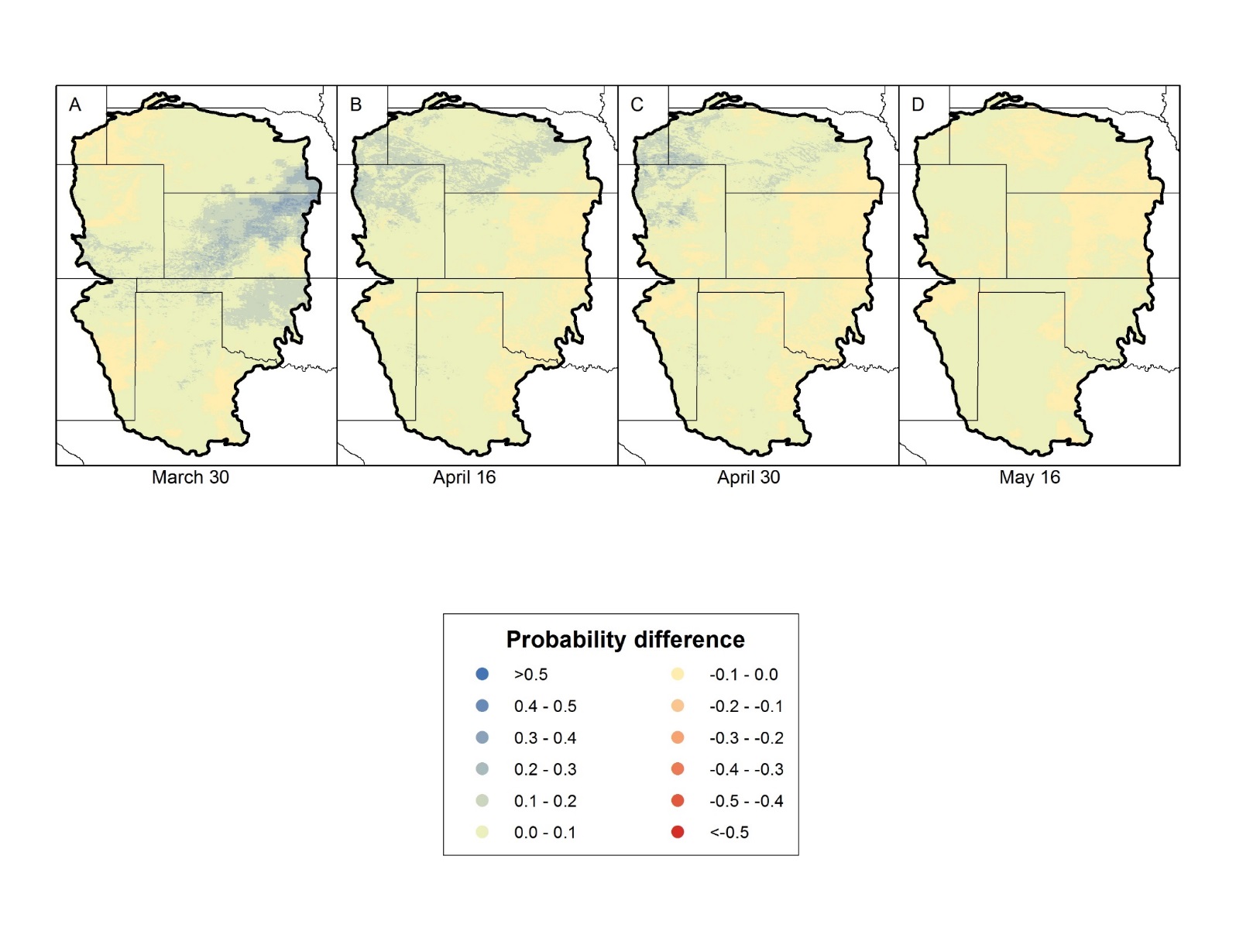

**
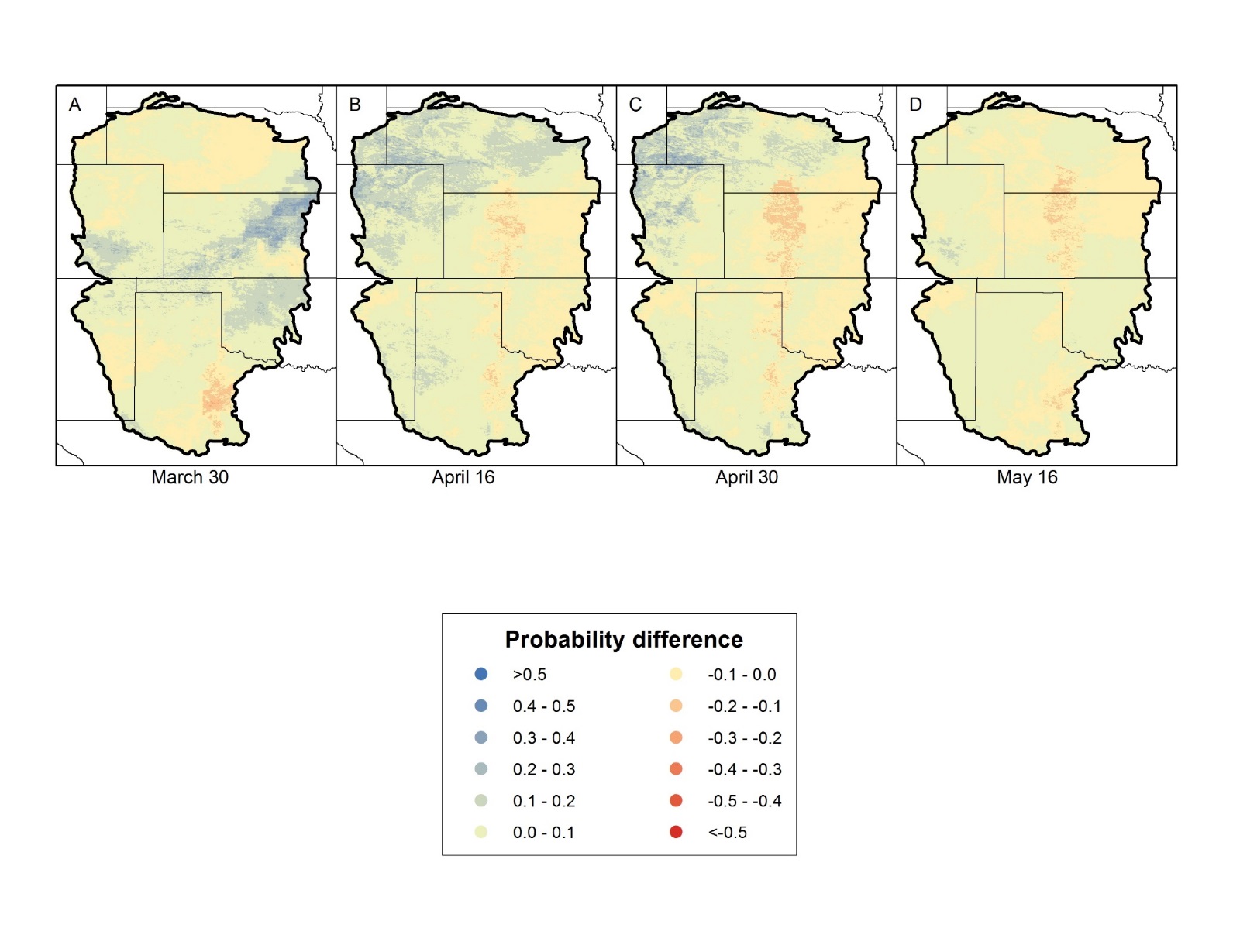
**

**Appendix 4j.** Probability of occurrence of Least Sandpiper, 1981-2010 (top panel) and projected changes based on the ensemble (middle panel) and hot dry ACCESS1-0 GCM (bottom panel).


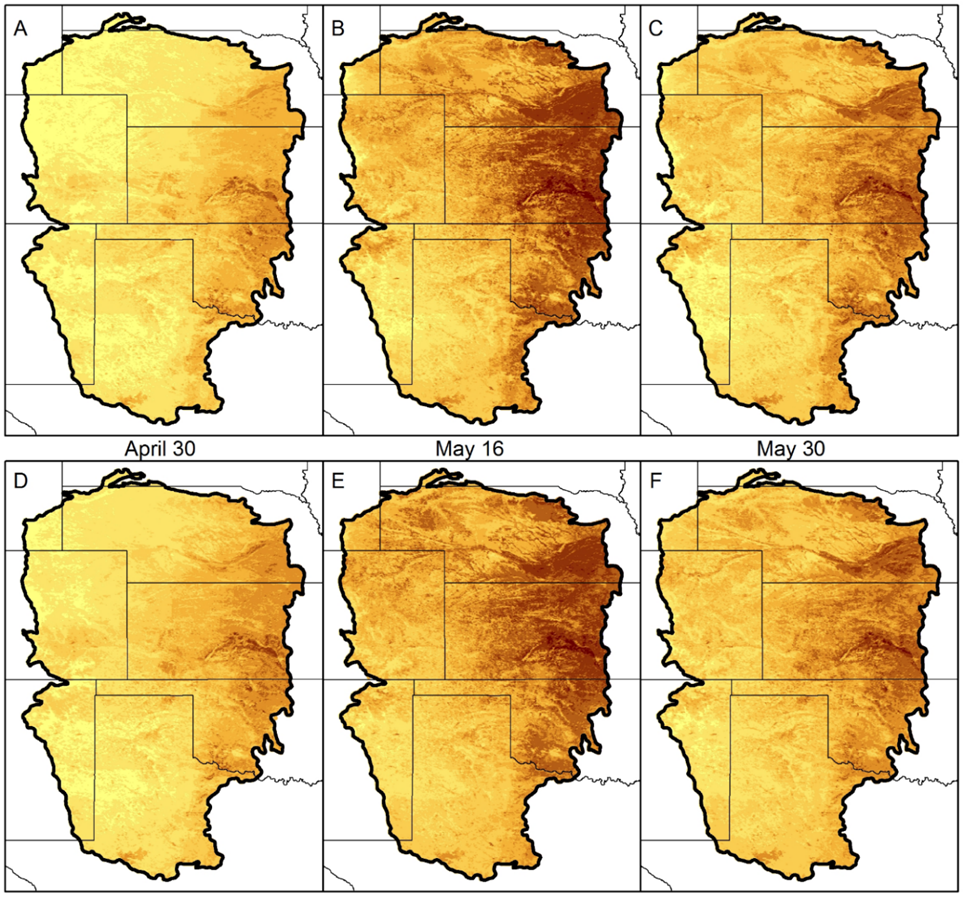


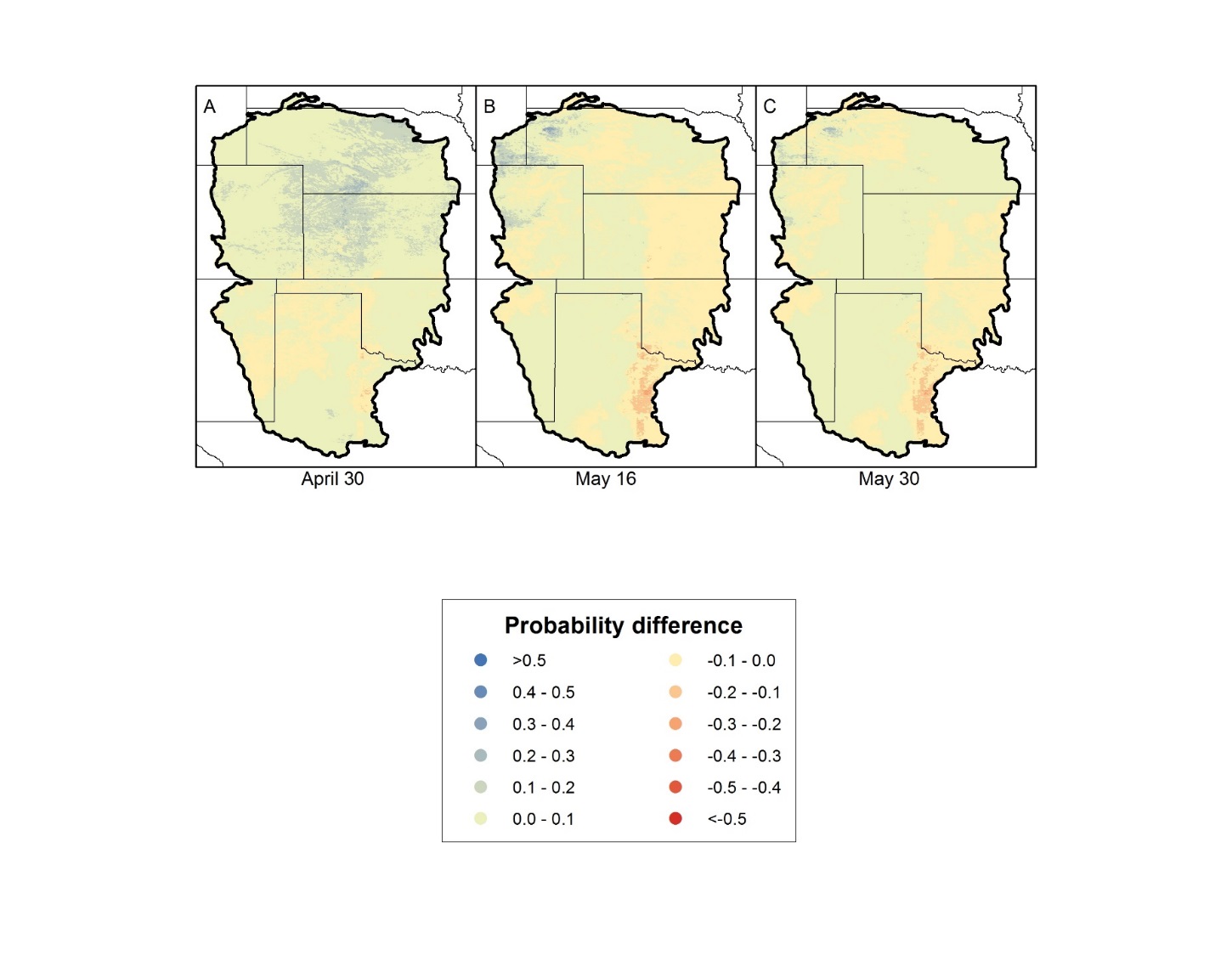

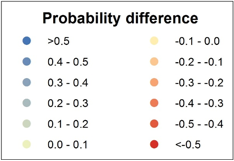

**
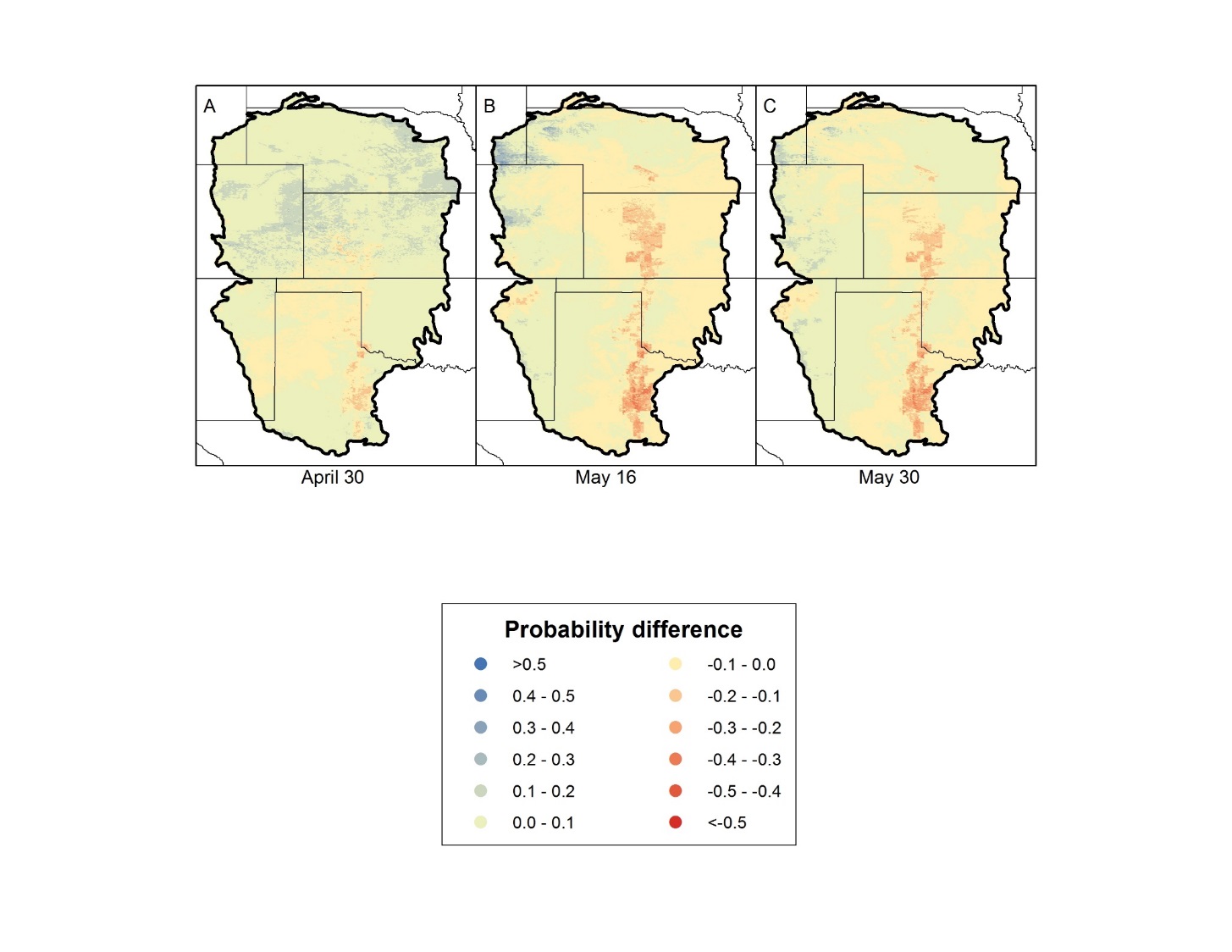
**

**Appendix 4k.** Probability of occurrence of White-rumped Sandpiper, 1981-2010 (top panel) and projected changes based on the ensemble (middle panel) and hot dry ACCESS1-0 GCM (bottom panel).


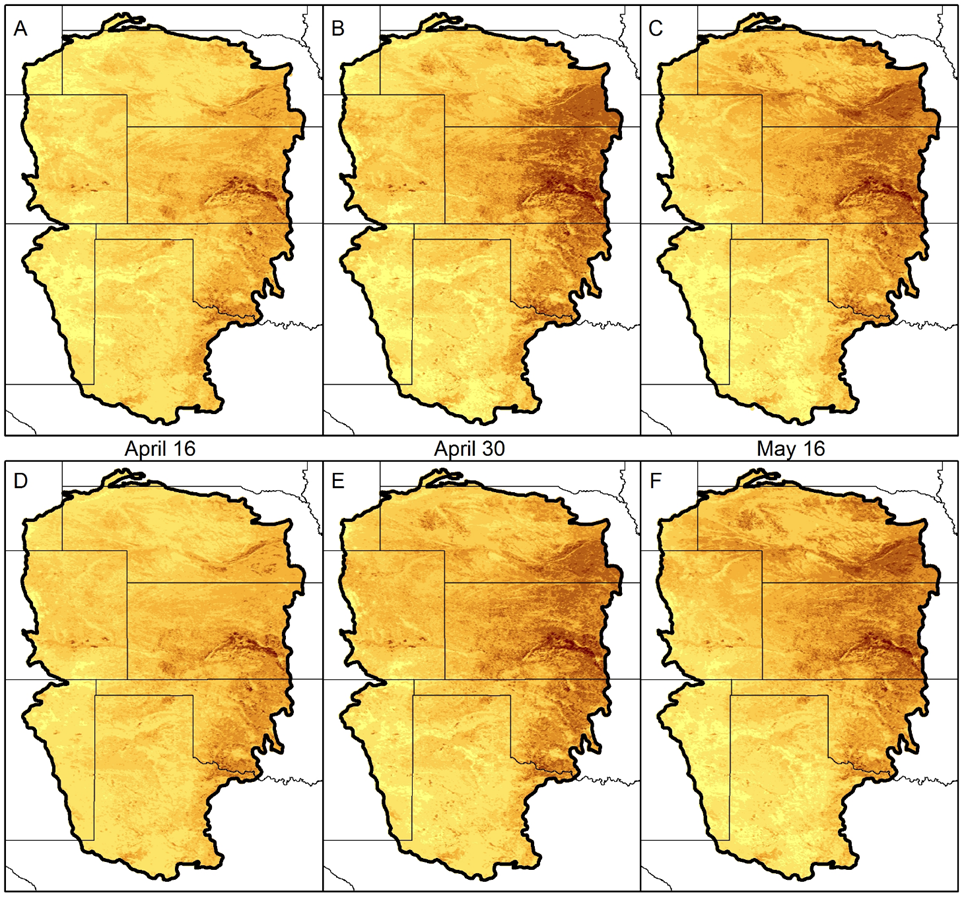


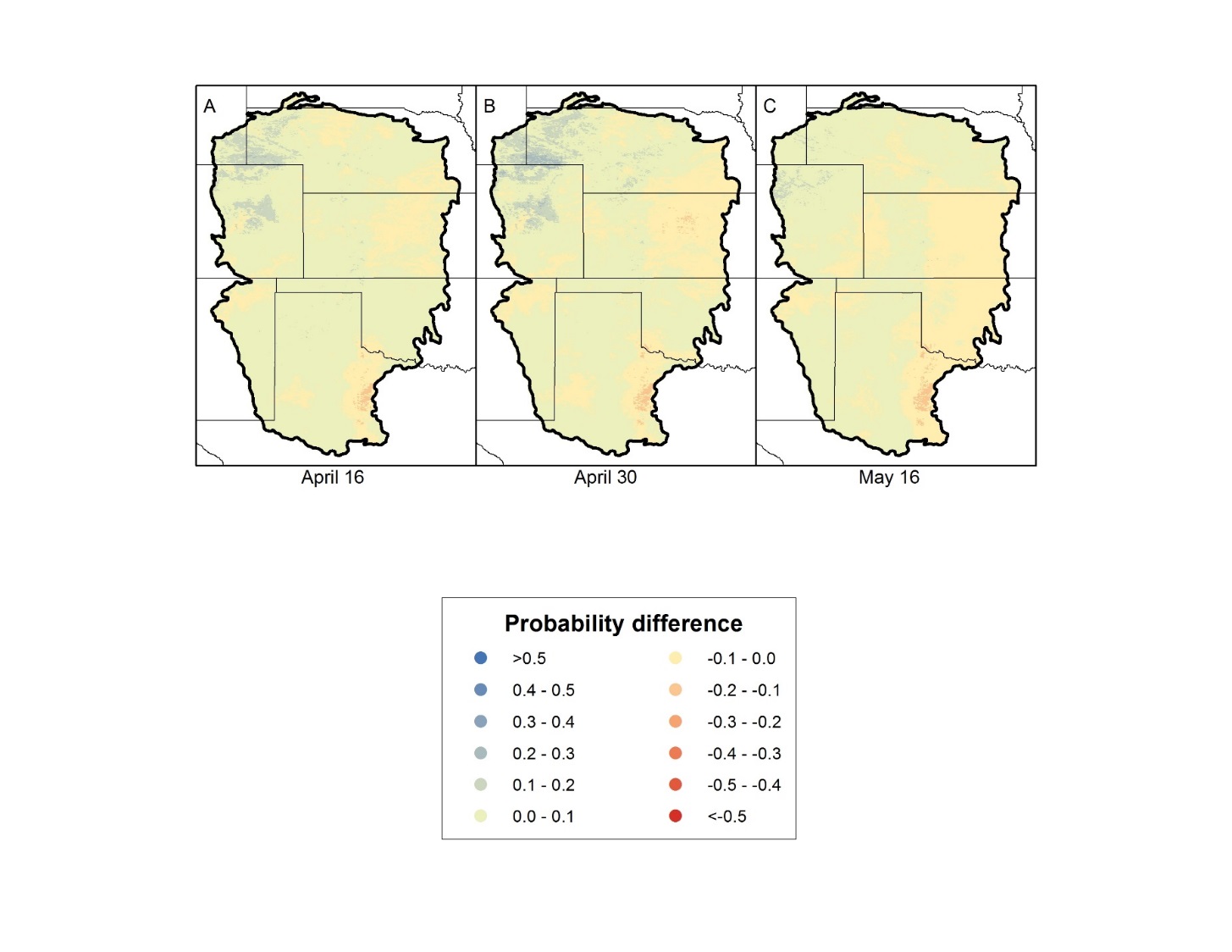

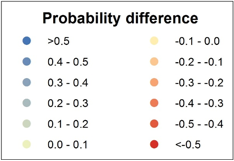

**
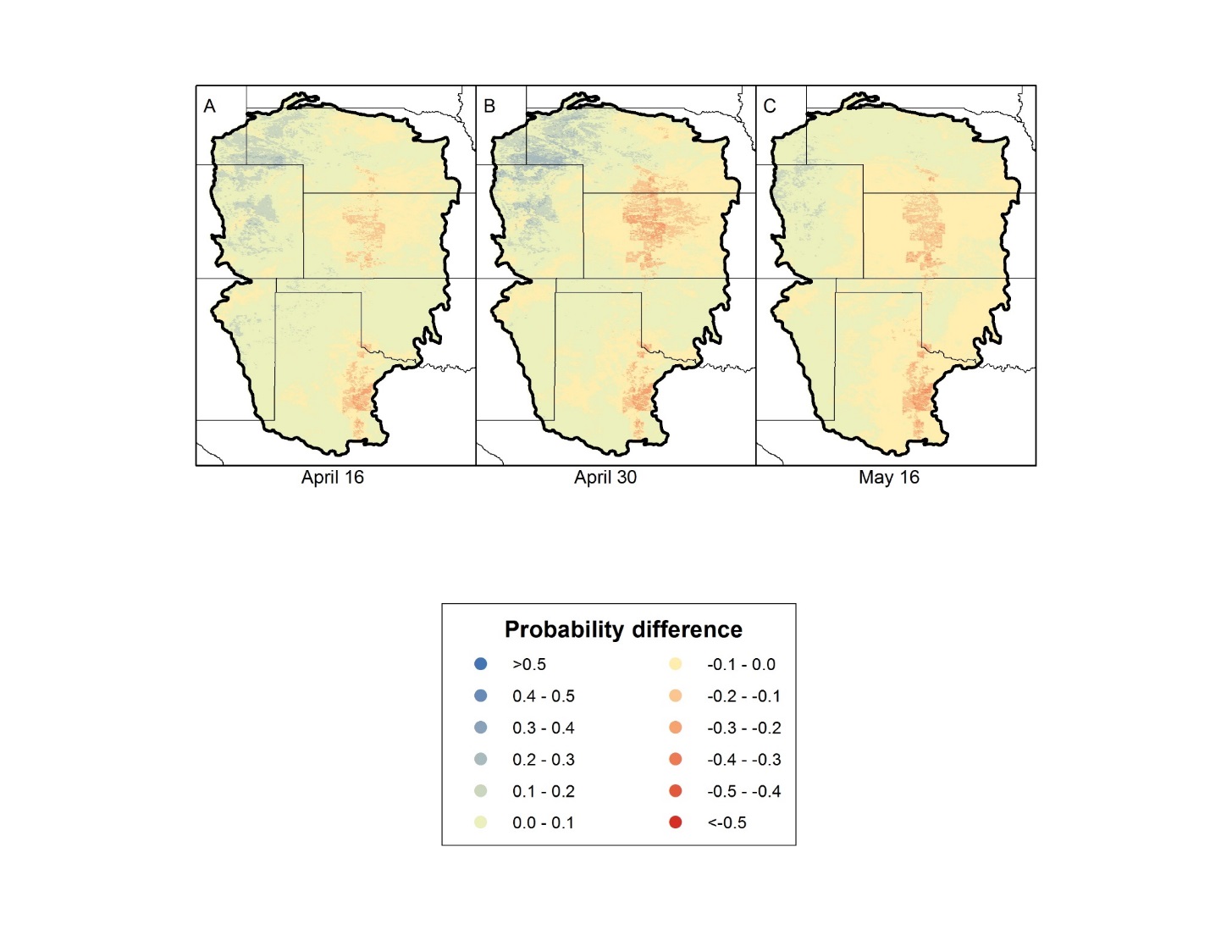
**

**Appendix 4l.** Probability of occurrence of Semipalmated Sandpiper, 1981-2010 (top panel) and projected changes based on the ensemble (middle panel) and hot dry ACCESS1-0 GCM (bottom panel).


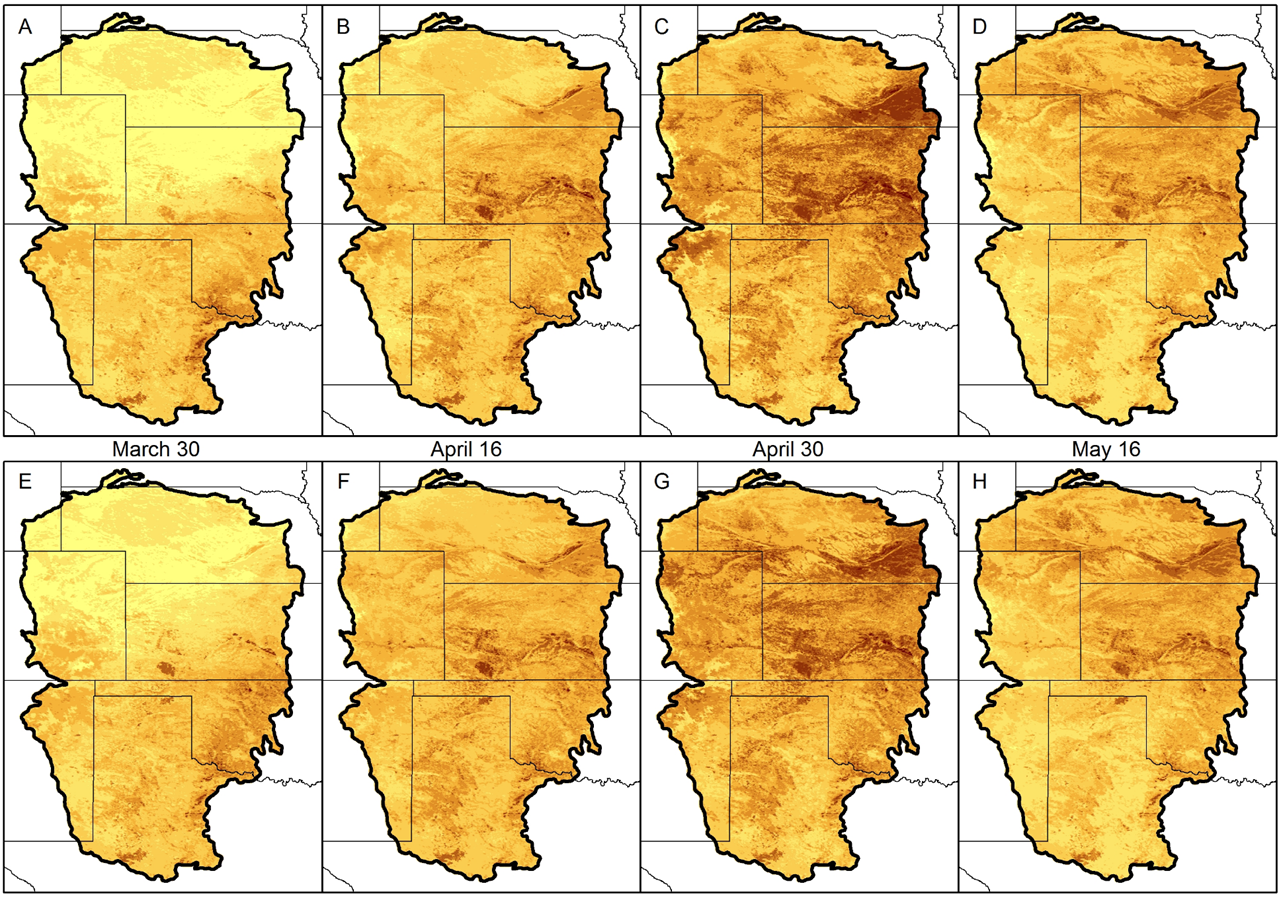


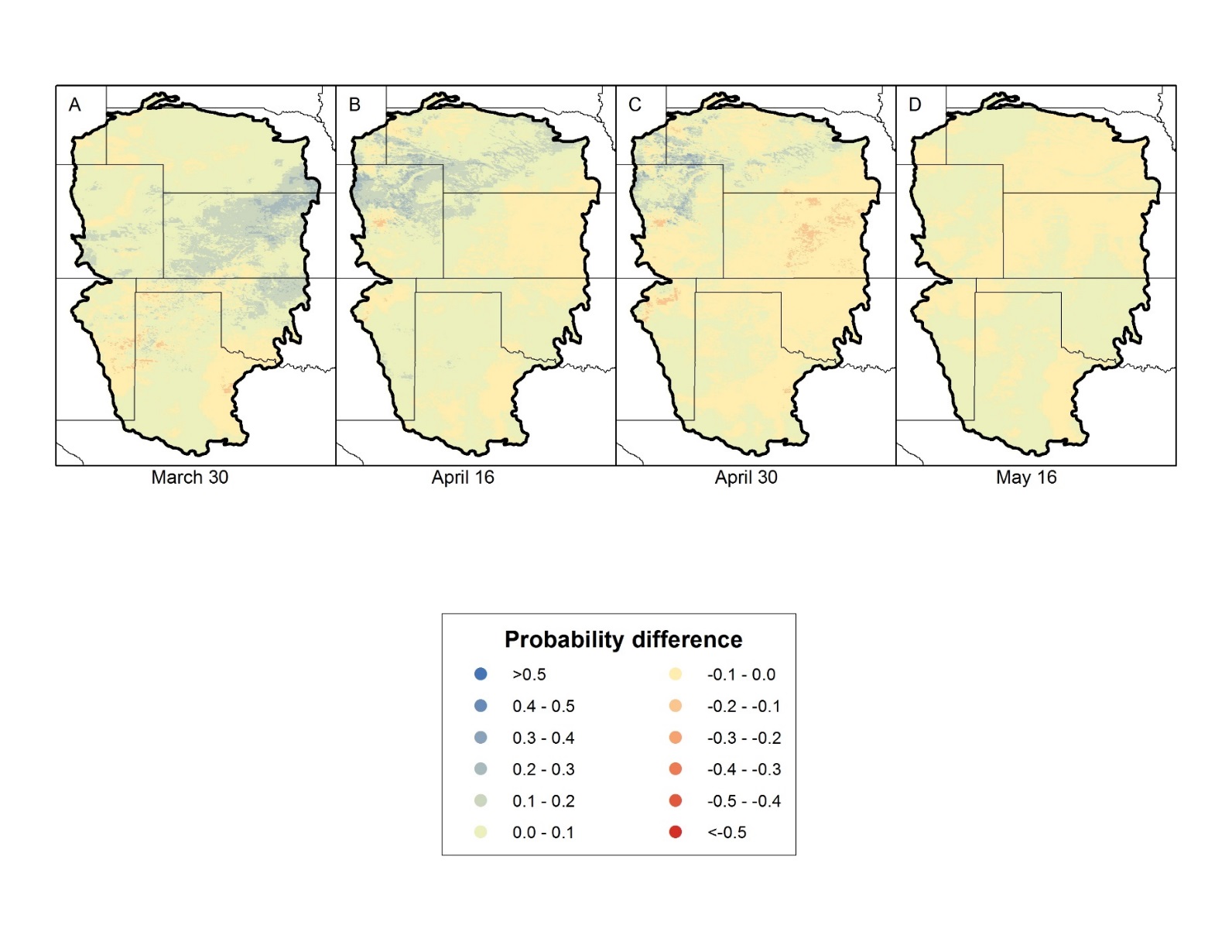

**
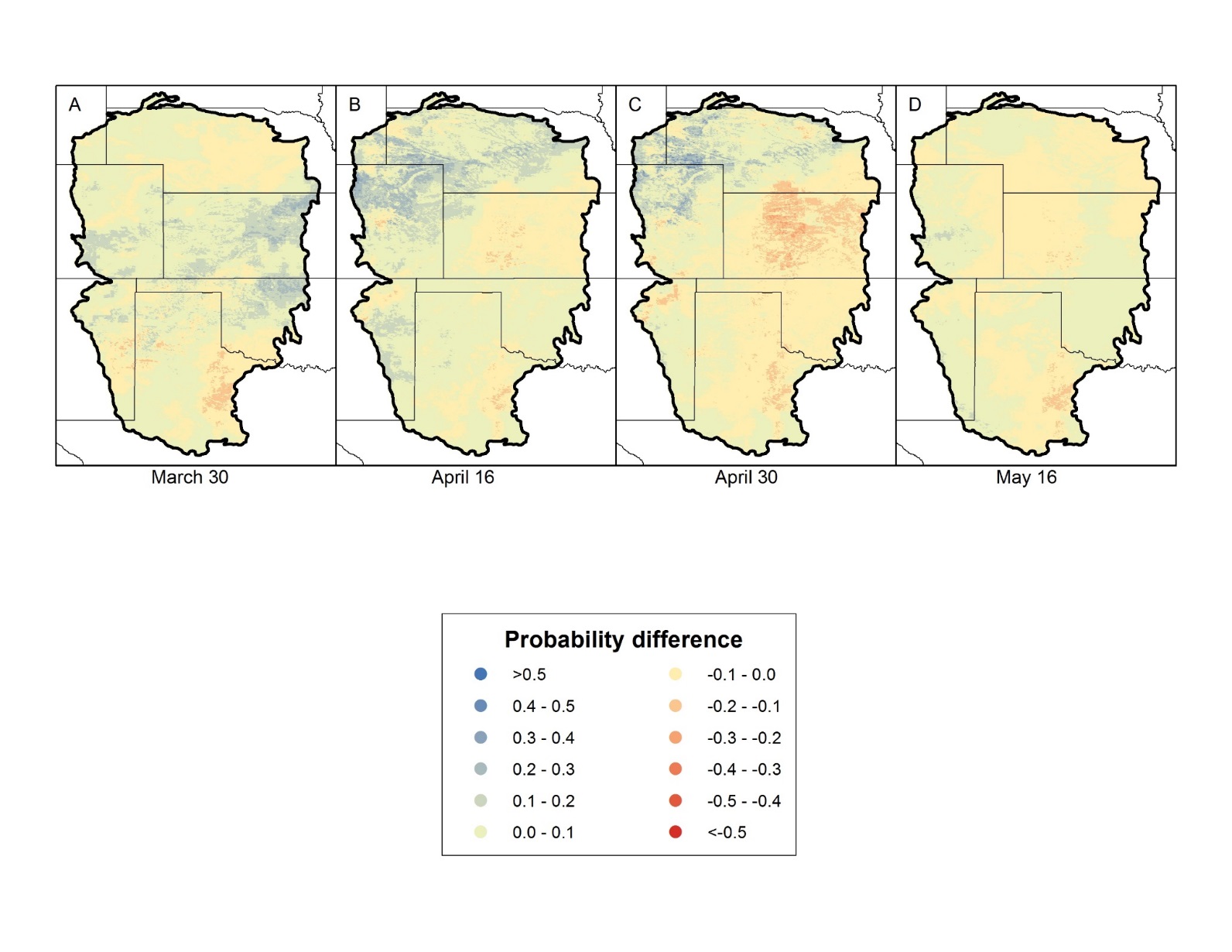
**

**Appendix 4m.** Probability of occurrence of Long-billed Dowitcher, 1981-2010 (top panel) and projected changes based on the ensemble (middle panel) and hot dry ACCESS1-0 GCM (bottom panel).


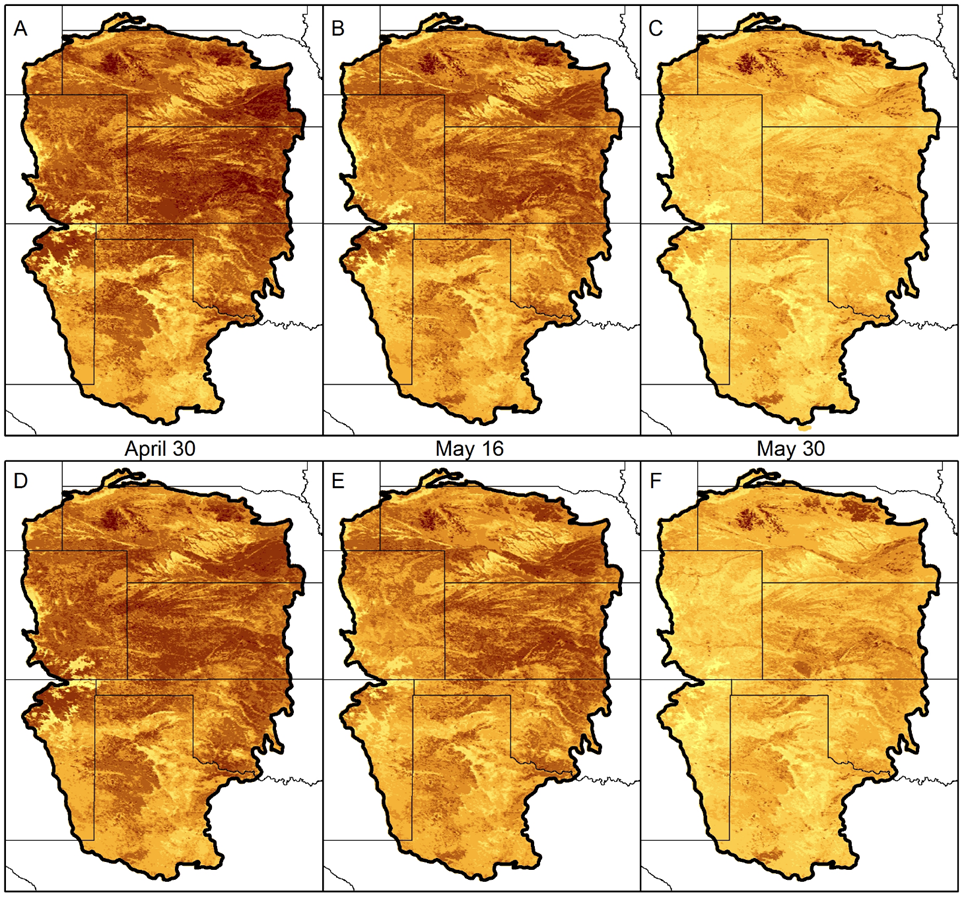


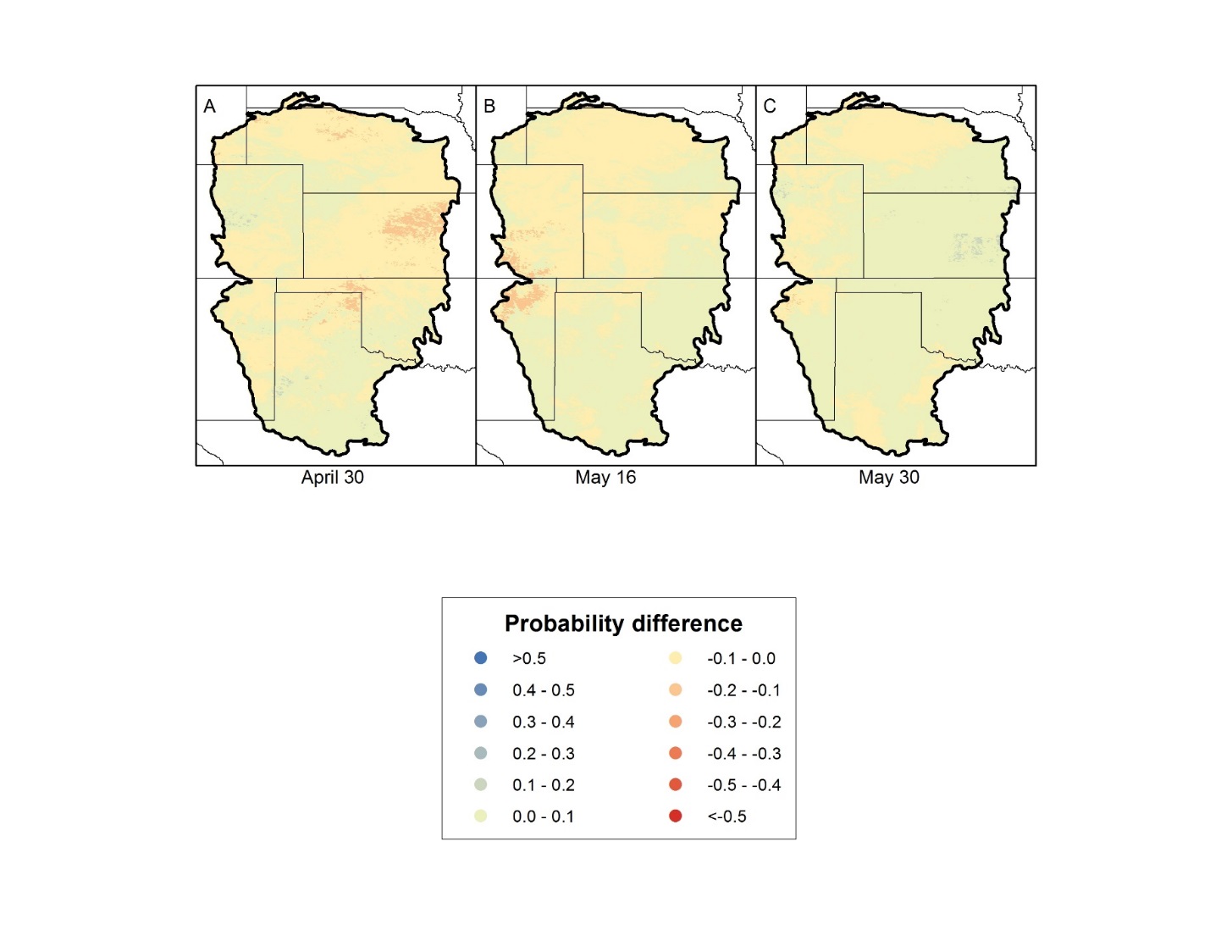

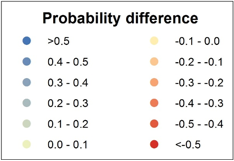

**
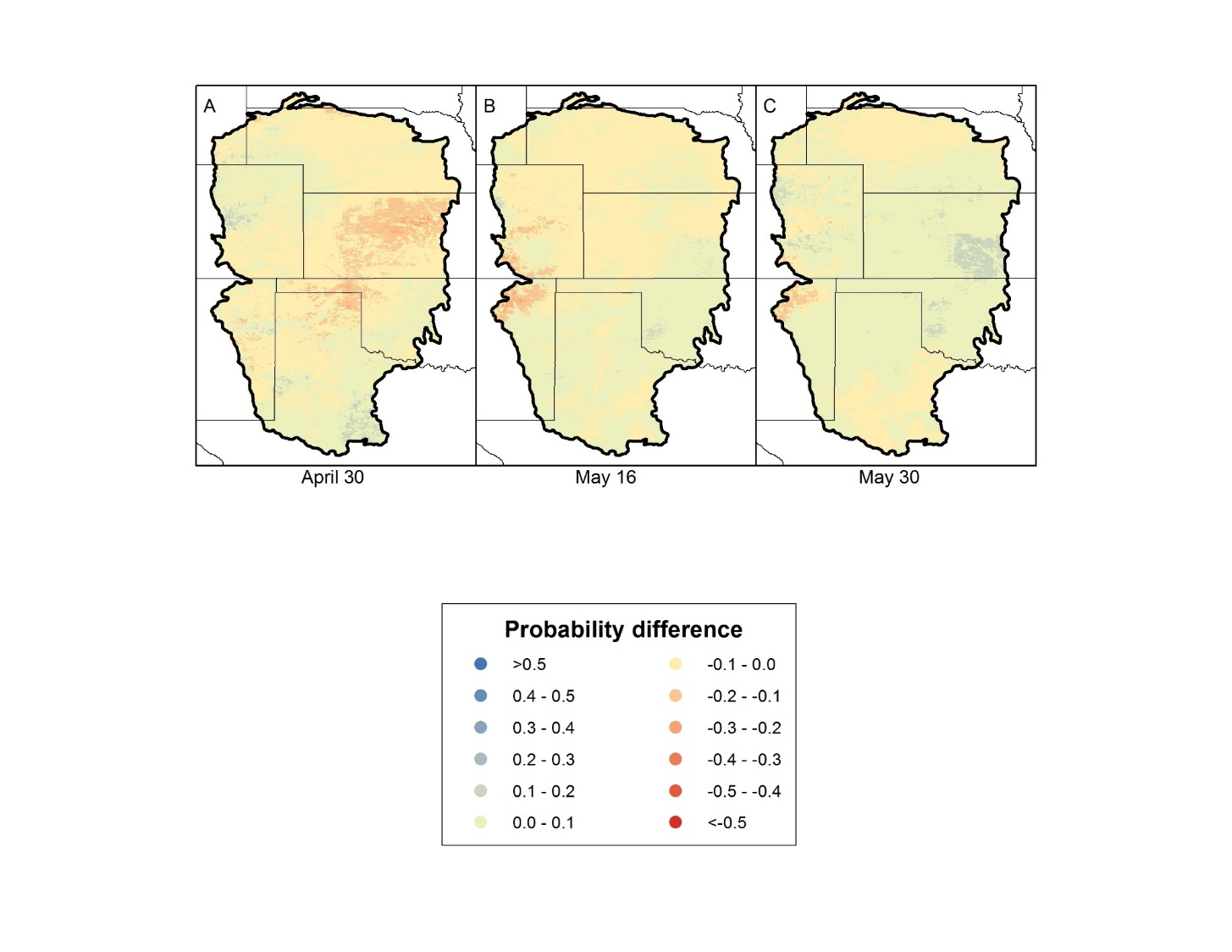
**

**Appendix 4n.** Probability of occurrence of Wilson’s Phalarope, 1981-2010 (top panel) and projected changes based on the ensemble (middle panel) and hot dry ACCESS1-0 GCM (bottom panel).
